# Single-cell transcriptomics in colorectal cancer uncover the potential of metastasis and immune dysregulation of a cell cluster overexpressed PRSS22

**DOI:** 10.3389/fimmu.2025.1586428

**Published:** 2025-05-20

**Authors:** Chengyuan Xu, Ziheng Zhou, Dongfei Zhu, Qingyun Zhang, Shoubin Zhong, Zhenhua Li

**Affiliations:** ^1^ School of Medicine, Tongji University, Shanghai, China; ^2^ The Department of Cardiology, Shanghai Sixth People’s Hospital Affiliated to Shanghai Jiao Tong University School of Medicine, Shanghai, China; ^3^ Department of Oncology, WeiFang People’s Hospital, Shandong Second Medical University, Weifang, Shandong, China

**Keywords:** colorectal cancer, single-cell sequencing, *PRSS22*, prognosis, metastasis, immune dysregulation

## Abstract

**Background:**

Colorectal cancer (CRC) is one of the most common malignancies worldwide, and its complex pathogenesis and significant tumor cell heterogeneity remain major challenges. With the rapid development of single-cell sequencing technology, we can now delve deeper into the cellular composition and dynamic changes within the tumor microenvironment, revealing cellular interactions and their potential roles in tumorigenesis.

**Method:**

In this study, we systematically analyzed comprehensive single-cell RNA sequencing data from 25 colorectal cancer and 10 adjacent normal tissue samples. We explored the characteristics and biological significance of tumor cell subpopulations, performed quality control, dimensionality reduction, and cell type identification, and further investigated epithelial cell copy number variations, cell communication, and pseudotime analysis. Subsequently, Boruta feature selection algorithm was combined to identify prognosis related genes. The expression patterns, clinical significance and biological effects of *PRSS22* were validated *in vitro*.

**Results:**

Our analysis found an epithelial cell subcluster with high expression of *PRSS22* exhibited high proliferation and migration abilities, and it was also associated with the dysregulated immune microenvironment. After further experimental verification, we proved the high expression patterns and clinical significance of *PRSS22*. Downregulation of *PRSS22* in CRC cells resulted in a reduction of proliferation, migration and invasion.

**Conclusion:**

Our study has identified a cell subcluster that is closely linked to progression, immune dysregulation and prognosis in CRC, and we have also identified *PRSS22* as its hub gene that has great potential to become a new immunotherapeutic targets target for CRC.

## 1 Introduction

Colorectal cancer (CRC) is acknowledged as the third most prevalent cancer worldwide and significantly contributes to cancer-associated deaths. Although the conventional treatment strategy involves surgical excision along with chemotherapy, nearly one-third of individuals undergoing this regimen face a recurrence of the disease (1, 2). Although immune checkpoint inhibitors have demonstrated substantial effectiveness in tumors characterized by high microsatellite instability (MSI), and the combination of EGFR/BRAF inhibitors has yielded positive outcomes in CRC) with BRAF V600E mutations, it is important to note that these therapeutic strategies are limited to particular subsets of patients (3–5). Large-scale gene expression studies have established molecular classification systems for CRC, most notably the Consensus Molecular Subtypes (CMS), which categorizes CRC into four subtypes: CMS1**–**4 (6). However, these classifications, which are primarily based on bulk sequencing data, cannot precisely resolve the complex cellular heterogeneity within the tumor microenvironment.

The development of CRC involves the accumulation of mutations in multiple oncogenes and tumor suppressor genes (such as APC, KRAS, and PIK3CA) and microsatellite instability caused by DNA mismatch repair gene dysfunction (3, 7). Although high tumor mutational burden (TMB) and MSI status can predict the response to immune checkpoint inhibitor therapy, only a minority of patients respond to PD-1 inhibitor treatment (8, 9). The complex molecular heterogeneity and microenvironmental characteristics of CRC not only influence disease progression but also present significant challenges for precision medicine, highlighting the importance of understanding the CRC microenvironment in detail. To date, there has not been a comprehensive and systematic characterization of how tumor and TME cells shape the tumoral, stromal, and immune landscapes to form specific CRC subtypes.

Recent single-cell studies have revealed cellular heterogeneity in the CRC microenvironment and identified multiple functionally important specific cell subgroups (10–13). While these studies have provided new perspectives for understanding tumor progression mechanisms and immune evasion, their geographical limitations and sample sizes make fully characterizing the shared mechanisms within the CRC microenvironment difficult. Cross-study comparisons are also challenging due to varying cell annotation methods across different studies. *PRSS22*, also known as *BSSP4*, has been the subject of very limited research to date. Regarding its association with cancer, several studies have reported its oncogenic role in liver cancer. However, in colorectal cancer and breast cancer, only a few isolated reports exist, and there is currently no research on its involvement in other types of cancer (14–17). This study further revealed the heterogeneity of CRC through single-cell sequencing (sc-seq) and explore the interactions between the TME from a precision medicine perspective, identifying *PRSS22* as new therapeutic targets, optimizing treatment strategies, and ultimately improving the prognosis of CRC patients.

## 2 Materials and methods

### 2.1 Data acquisition and quality control

Single-cell RNA sequencing data used in this study were obtained from GEO database (accession number: GSE132465), which includes 25 colorectal cancer samples and 10 adjacent non-tumor control samples, consisting of 33,694 genes and 63,689 cells in total. Basic quality control (QC) was performed to ensure accuracy and reliability in the analysis. The quality control criteria were as follows: nFeatureRNA was between 300 and 5000, mitochondrial gene expression percentage (percent_mito) was less than 20%, ribosomal gene expression percentage (percent_ribo) was greater than 1%, and each gene was expressed in at least three cells. After applying these standards, a total of 25,051 genes and 60,883 cells were retained for further analysis. The results are shown in **Supplementary Figure S1**.

### 2.2 Data integration, dimensionality reduction, and clustering

We integrated the filtered count matrices from 35 samples using the R package harmony (v1.0) approach to correct for batch effects and integration. After integration, principal component analysis (PCA) was performed on the integrated data followed by embedding into low-dimensional space with Uniform Manifold Approximation and Projection (UMAP) based on the top 15 dimensions. Clusters were generated by graph-based method using the FindClusters function from the Seurat package (v4.3.0) and assigned to cell types by consulting the expression of known marker genes and automatic annotation from the SingleR package (2.8.0).

### 2.3 Estimation of chromosomal copy number variations

Chromosomal copy number variations (CNVs) were estimated using R package “inferCNV” (v1.22.0). B cells, T cells, myeloid cells and benign epithelial cells were used as references. The CNV score was obtained by summing the CNV levels of cells within each subcluster. The threshold parameter for ‘inferCNV’ was set at 0.1, with all other parameters at their default values. We used a five-category classification method for CNV assessment in this study. We assigned one point for gain or loss of a single copy number and two points for gain or loss of two or more copy numbers. These points were then summed to obtain the total CNV score.

### 2.4 Differential expression analysis and Boruta feature selection process

Pseudo-bulk differential expression analysis between primary and metastatic samples was performed using R package ‘DESeq2’ (v1.46.0). Genes with absolute log2 fold changes >1 and adjusted p-values <0.01 were considered as differentially expressed genes (DEGs) (18). Additionally, Boruta is a feature selection algorithm that systematically introduces random perturbations to each actual feature, assesses their significance, and iteratively eliminates those with low correlation to identify the most relevant variables. In this study, the Boruta package (version 7.0.0) was employed for feature selection.

### 2.5 Gene set functional analysis

Gene set functional analyses were performed using R packages ‘clusterProfiler’ (v4.14.4) and ‘GSVA’ (v2.0.5). The GSVA analysis utilized Gene Ontology (GO), Kyoto Encyclopedia of Genes and Genomes (KEGG), and Reactome pathway databases. Hallmark gene sets and Reactome gene sets were sourced from the R package ‘msigdbr’. Additionally, we performed Gene Set Enrichment Analysis (GSEA) to further understand the pathways in the high and low PRSS22 expression group by GSEA 4.1 software.

### 2.6 Cell–cell communication analysis

Cell–cell communication analysis was conducted using R package ‘CellChat’ (v1.1.3). For the analysis, 500 cells from each cell subcluster were randomly selected using the ‘subset’ function. The ligand-receptor interaction database including ‘Secreted Signaling’, ‘ECM-Receptor’ and ‘Cell–Cell Contact’ pathways, were used for the analysis. A minimum cell count of 10 was set as the filtering threshold (19).

### 2.7 Pseudotime analysis

Pseudotime analysis was performed using R package ‘SlingShot’ (v2.14.0) to construct a single-cell pseudotime trajectory. Dimensionality reduction was achieved using the UMAP method, and the ‘plot_cells’ function was used for visualization. Differentially expressed genes (DEGs) were identified using the ‘associationTest’ function and decreasingly sorted by their q-value.

### 2.8 Immunological correlation evaluation

CIBERSORT, a deconvolution algorithm, was utilized to estimate the proportions of tumor-infiltrating immune cells in tumor tissues with mixed cell types. ESTIMATE was used to estimate stromal and immune cells in malignant tumors, as well as calculate tumor purity, stromal score, immune score, and ESTIMATE score. Additionally, the ssGSEA algorithm was employed to evaluate immune cell infiltration and immune-related functions.

### 2.9 Patients and clinical samples

Fifty-five pairs of cancer tissues were collected from patients who underwent colorectal cancer surgery at Yangpu Hospital (35 pairs of rectal cancer and 20 pairs of colon cancer) at Tongji University between November 2018 and November 2020. This study was approved by the Ethics Committee of Yangpu Hospital (LL-2023-LW-012). Colorectal cancer (CRC) tissues and adjacent non-cancerous tissues were obtained during surgery and immediately frozen in liquid nitrogen for subsequent analysis of specific gene and protein expression.

### 2.10 Quantitative real-time PCR and western blotting

Total RNA was extracted from paired colorectal cancer tissues. The RNA was then reverse transcribed into cDNA using a kit (Takara, Dalian, China) and amplified. The primer sequences are as follows:

*PRSS22*:5’-TGTCTCGGCACCTTCACCT-3’ and 5’-GAATACACAGGGTGGGGCT C-3’.
*GAPDH*:5’-ACACCCACTCCTCCACCTTT-3’and 5’-TTACTCCTTGGAGGCCATG T-3’.

Total cellular protein from clinical samples was extracted using RIPA lysis buffer (Solarbio, China), with the addition of a protease inhibitor at a 1:100 ratio (Thermo Scientific, USA).

The primary antibodies used were:

*PRSS22* (1:1,000, HUABIO, ER60535); β-actin (1:4,000, Santa, sc-47778).

Human colorectal cancer (CRC) cell lines (HCT15, RKO) were obtained from the Shanghai Institute of Biochemistry and Cell Biology. All cell lines were cultured in DMEM medium (Gibco, Carlsbad, CA, USA) supplemented with 10% fetal bovine serum (FBS; Gibco) at 37°C with 5% CO_2_.

To investigate the role of *PRSS22*, small interfering RNA (siRNA) targeting *PRSS22* was designed and synthesized by Shanghai Ruimian Biotechnology Co, Ltd. The siRNA was transfected into HCT15 and RKO cells using Lipofectamine 3000 reagent (Invitrogen, Carlsbad, CA, USA) for *PRSS22* knockdown. The siRNA sequences were:

siPRSS22-1: GGAUCGUGAGCAUCCAGAATT.
siPRSS22-2: UCUAUCCACCUCCCUCCAATT.

### 2.11 Immunohistochemistry

For immunohistochemistry assay, 25 pairs of clinical specimens were fixed in paraffin and cut into 4 µm tissue slices. After being dewaxed and dehydrated, the tissue sections were subjected to antigen retrieval using the thermal method for 30 minutes. The samples were then incubated with 3% hydrogen peroxide for 20 minutes, followed by 5% BSA for 40 minutes. Afterward, they were incubated with the appropriate antibodies. The information of primary antibody was as follows: *PRSS22* (1:100, HUABIO, ER60535).

### 2.12 Cell proliferation assay

Cell proliferation was assessed using the Cell Counting Kit-8 (CCK-8) assay. Transfected cells were seeded into a 96-well plate at a density of 3,000 cells per well. Cell viability was measured using the CCK-8 system (Beyotime Institute of Biotechnology, China), and absorbance at 450 nm was recorded using a microplate reader (SpectraMax i5x, Molecular Devices).

### 2.13 Colony formation assay

Approximately 1,000 cells were seeded in each well of a 6-well plate and cultured for 7–14 days until visible colonies formed. The cells were fixed with 4% paraformaldehyde for 15 minutes and stained with 0.1% crystal violet (Sangon Biotech) for 30 minutes at room temperature. Colonies were imaged using a light microscope at ×100 magnification (Nikon Corporation, Japan). The number of colonies in five random, non-overlapping fields of view was counted and averaged.

### 2.14 Transwell assays and wound healing assay

Cells were suspended in 250 μL of serum-free medium and seeded into the upper chamber of a 24-well Transwell plate (Nest, China). The lower chamber was filled with culture medium containing 10% FBS. For invasion assays, the Transwell chambers were coated with Matrigel (2 mg/mL) and DMEM, whereas for migration assays, they were left uncoated. After 24 hours of incubation, the invaded cells were fixed with 4% paraformaldehyde for 30 minutes and stained with crystal violet for 10 additional minutes, both at room temperature. Cells were counted in five random optical fields of view under a light microscope (Nikon Corporation, Japan).

For the wound healing assay, cells were cultured without FBS in 6-well plates for 24 hours. Linear wounds were created by scratching with a 10 μL pipette tip. Wound closure was monitored and photographed at 0 and 24 hours using a microscope (Nikon Corporation, Japan).

### 2.15 Statistical analysis

All statistical analyses and data visualizations were performed using R software (version 4.2.1). For quantitative data, a two-tailed unpaired Student’s t-test or one-way analysis of variance (ANOVA) with Tukey’s multiple comparison test was performed to compare values between subgroups. When multiple comparisons were conducted, p-values were adjusted using the Benjamini-Hochberg (BH) method to control the false discovery rate (FDR). A p-value or adjusted p-value < 0.05 was considered statistically significant.

## 3 Result

### 3.1 Preliminary annotation and identification of cancer cell subtypes

**Figure 1:**
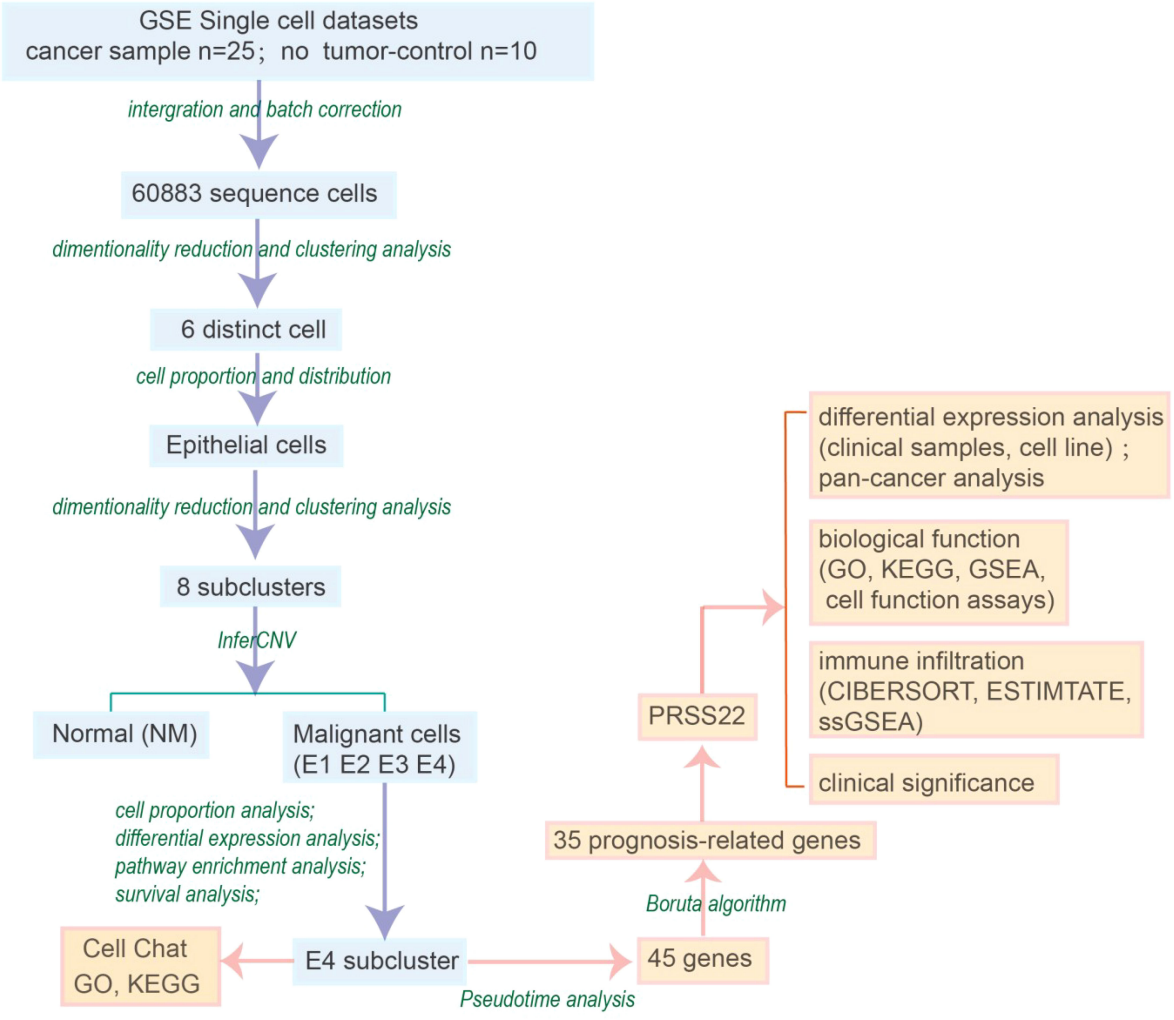
Flowchart of the manuscript.

**Figure 2:**
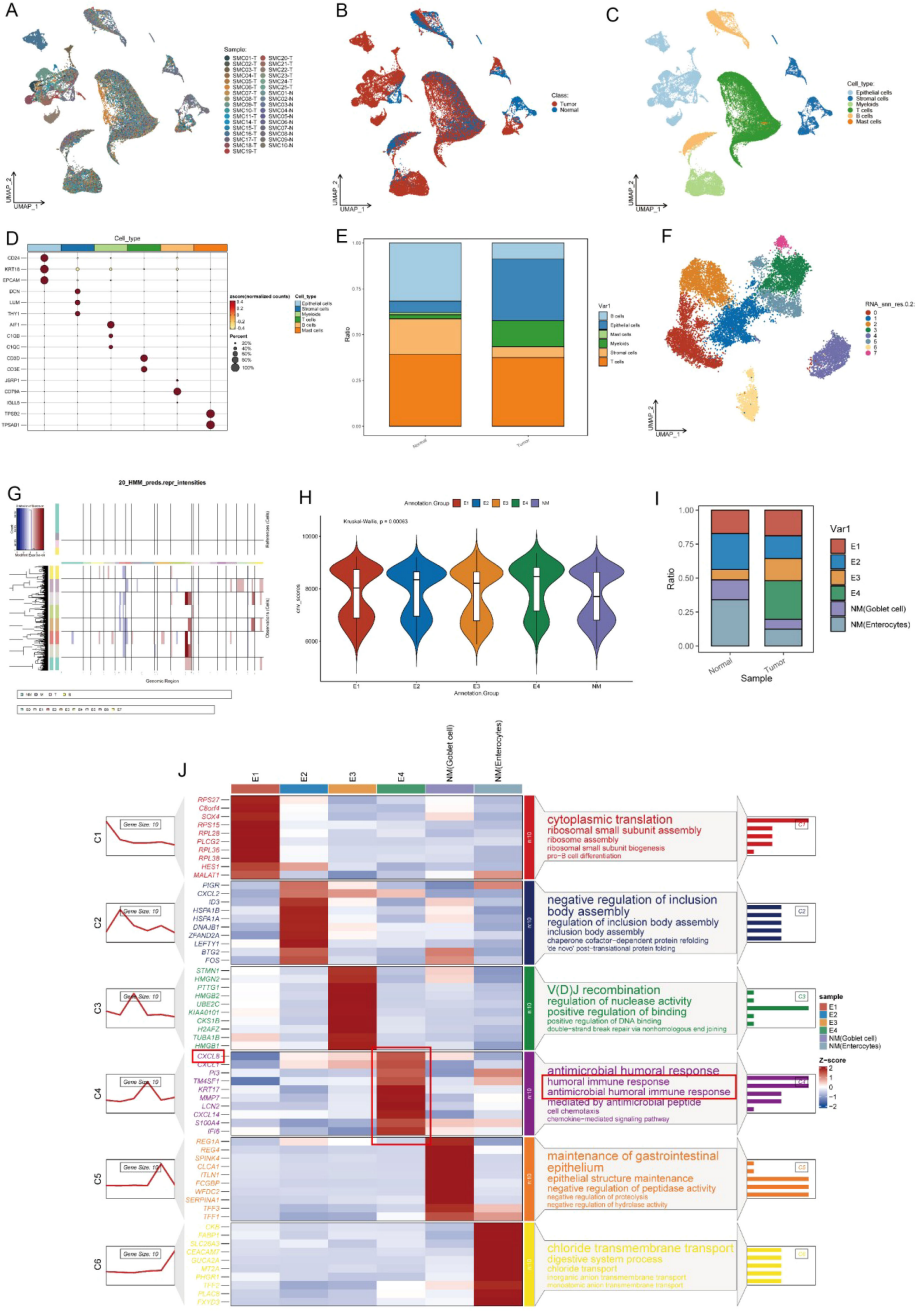
Preliminary annotation and identification of subtypes of cancer cells. **(A-C)** UMAP visualization of colorectal cancer (n=25) and paired normal mucosa (n=10). **(D)** Expression of marker genes used for the identification of each cluster. **(E)** Bar plot showed the cell proportion among tissue types. **(F)** UMAP showing subtypes of epithelial cells. **(G)** Chromosomal landscape of inferred CNVs among cancer cell subclusters. **(H)** Violin plot demonstrated the difference in CNV scores among benign and malignant cell subclusters. **(I)** Stacked bar plot represented the proportional distribution of cell types across different groups. **(J)** Heatmap shows markers and enriched gene ontology pathways among subtypes of epithelial cells.

By analyzing the single-cell sequencing results of 25 tumor patients and 10 healthy individuals, and after integration and batch correction, we have obtained a total of 60,883 sequenced cells that met quality control metrics (**Supplementary Figure S1**). The flowchart of the analysis was shown in **Figure 1**. Firstly, we found that there was a strong heterogeneity within different tumor patients (**Figures 2A, B**), which may also partly explain why different cancer patients have different treatment methods, strategies, and outcomes. To accurately classify the cell types obtained from UMAP embedding analysis, we examined the differentially expressed gene signatures of each cluster and cross-referenced them with known markers. Based on classical cell markers, 6 distinct cell types were identified and classified as follows: epithelial cells, stromal cells, myeloid cells, T cells, B cells, and mast cells (**Figures 2C, D**). Further distribution of cell proportions (**Figure 2E**) revealed a significant increase in the proportion of epithelial cells in tumor samples, indicating a correlation between tumor development and increased heterogeneity of epithelial cells. After extracting the epithelial cells, the R package Harmony was used to correct for batch effects, and secondary dimensionality reduction and clustering analysis were performed. The epithelial cells were divided into 8 clusters (**Figure 2F**). Copy number variation (CNV) analysis was then performed on the epithelial cells using R package ‘inferCNV’ to assess CNV levels and heterogeneity among different epithelial cells. Based on the CNV levels, the 8 subpopulations were identified as normal epithelial cells (including goblet cells and intestinal epithelial cells, marked as NM) and 4 categories of cancerous epithelial cells (E1-E4) (**Figure 2G**). Cells with high copy-variation level were defined as malignant cells. The copy number variation levels of each subgroup are shown in (**Figure 2H**). The results indicate that the copy number variation levels of the tumor subgroups E1 to E4 are higher than those of the NM group, with subgroup E4 showing the highest level of copy number variation. It is therefore hypothesized that tumor subgroup E4 may possess higher individualized potential. The cell proportion analysis (**Figure 2I**) showed that E2 was more abundant in adjacent non-tumor tissues, while E1, E3, and E4 were enriched in tumor tissues. Notably, E4 was a tumor-specific subgroup. On the one hand, this reflects the high heterogeneity of the tumor; on the other hand, we speculate that E2 may represent an intermediate state in the transition from normal to malignant cells and include early-stage tumor cells, whereas E4 likely corresponds to highly malignant, late-stage tumor cells.

We further analyzed the gene expression differences between tumor cells and normal epithelial cells using a heatmap (**Figure 2J**) and a volcano plot (**Supplementary Figure S2**). In the C1 cluster (ribosomal small subunit assembly), genes such as *RPS27* and *RPL36* were significantly upregulated, indicating enhanced protein synthesis in tumor cells, which supports rapid proliferation. The C2 cluster (heat shock proteins and molecular chaperones) showed high expression of *HSPA1B* and *DNAJB1*, suggesting that tumor cells experience high biological stress and rely on these proteins to maintain protein folding homeostasis (20). The C3 cluster was associated with immune gene rearrangement and DNA repair. The C4 cluster was linked to humoral immune response and chemokine-mediated immune responses. In the C4 cluster, E4 cells showed increased expression of immune evasion genes such as *CXCL8*, which may help contribute to tumor growth by escaping from evade immune surveillance, enhancing immune resistance and angiogenesis ability (**Figure 2J**) (21–23).

In the C5 cluster (intestinal barrier maintenance), *REG1A* and *TFF1* were highly expressed in normal epithelial cells, indicating their role in maintaining epithelial integrity. However, these genes were significantly downregulated in tumor cells, which may lead to barrier dysfunction and promote tumor invasion and metastasis. The C6 cluster (metabolic reprogramming) included chloride transport-related genes such as *SLC26A3*, which were specifically expressed in normal epithelial cells. The volcano plot (**Supplementary Figure S2**) showed high expression of tumor markers such as *SLC2A1* and *BCL2L1* in E4, suggesting a strong proliferative advantage and anti-apoptotic ability in tumor progression (24, 25).

### 3.2 Survival analysis and cell chat analysis of the subclusters

**Figure 3:**
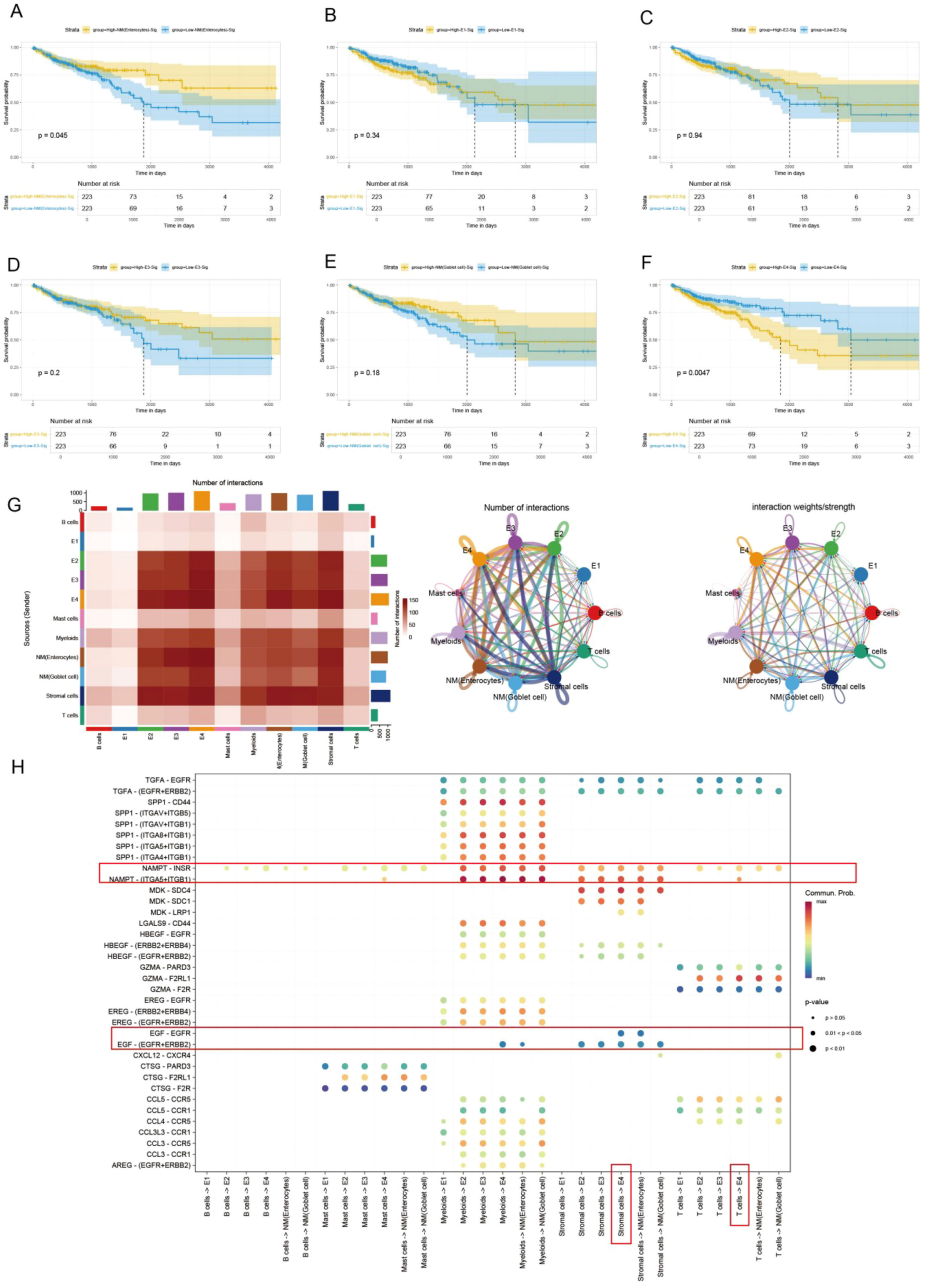
Kaplan-Meier curves and CellChat analysis reveals the prognosis and diverse crosstalk between different subtypes of cancer and tumor microenvironment cells. **(A-F)** K-M curves of different subclusters. **(G)** Heatmap plots showing the incoming and outgoing signal strength of cell subtypes in tumor and normal groups, illustrating the roles of each subtype in the signaling network. **(H)** Bubble plots showing the details of ligands and receptors expression.

Based on the above analysis, the characteristic marker genes of each subgroup were obtained, and the set of these characteristic genes was used as the signature. Each subgroup corresponds to its own signature, and the GSVA algorithm was used to calculate the level of signature in the TCGA cohort. Finally, each patient obtained the signature value of E1-E4 and NM cells and was divided into high expression group and low expression group for survival analysis according to **Figures 3A-F**.

The Kaplan-Meier survival curves have shown that high expression of NM (enterocytes) was associated with good prognosis (P=0.045) (**Figure 3A**), while E4 was associated with poor prognosis (P=0.0047) (**Figure 3F**) in the TCGA dataset. This also indicates the clinical significance of the E4 subtype.

Since cell−cell interactions always influenced cellular behaviors and fates, the potential interactions of the heterogeneity of different subpopulations were investigated (**Figure 3**). The different characteristics of signal input and output across various cell types are shown in **Figure 3G**. Tumor cells (E2-E4) exhibit strong input and output signals, especially E4, whose signal output and input intensities are most pronounced, indicating that these tumor cells are in an actively regulated state, which is crucial for tumor growth and immune escape. Next, we analyzed the interactions between different ligands and receptors in various cell types (**Figure 3H**), E4 subpopulation exhibited relatively unique interactions between receptors and ligands compared to other cells. For example, E4 group interacts with the NAMPT signal from immune cells. Previous studies have shown that NAMPT plays a crucial role in promoting the progression of colorectal cancer and is associated with patient prognosis (26–28). Additionally, as a rate-limiting enzyme in NADPH metabolism, it can directly activate PD1, thereby evading immune surveillance (29). Besides, the EGFR signaling pathway is a highly classical pathway in tumor immunity, closely associated with various forms of immune dysregulation in multiple types of cancer (30–34). This strongly suggests that immune dysfunction in the CRC microenvironment may be related to the interactions between the E4 subgroup and stromal cells.

### 3.4 Pseudotime analysis using slingshot reveals the lineage of epithelial cell transition

**Figure 4:**
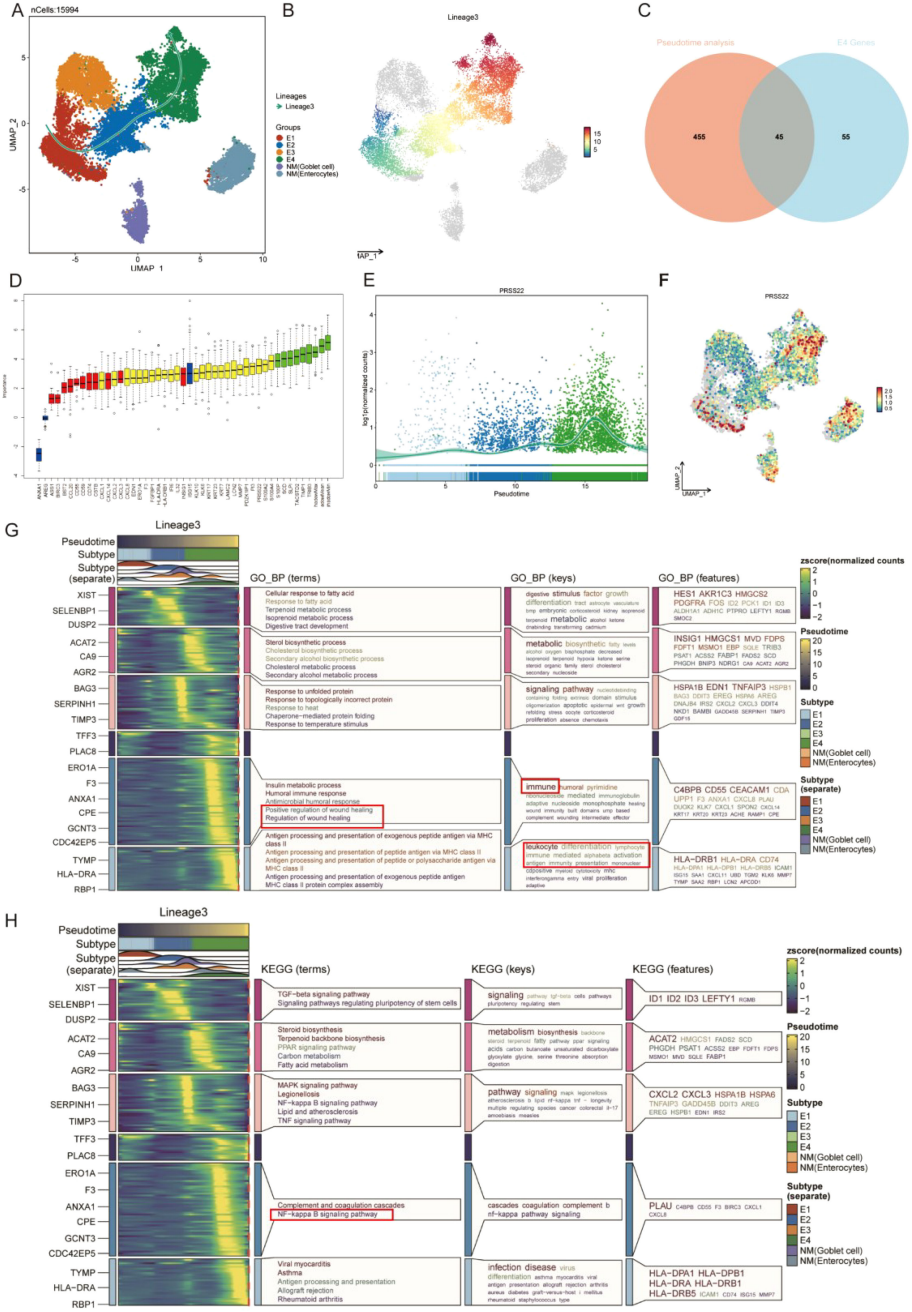
Pseudotime analysis based on Slingshot reveals the lineage of epithelial cell transition. **(A)** Trajectory of epithelial cells transition from E1 to E4. **(B)** UMAP visualization colored by pseudotime in lineage 3. **(C)** Venn Diagram obtains the intersected genes. **(D)** 35 genes were accepted as prognosis genes by Boruta algorithm **(E)** PRSS22 expression is consistent with the transition trajectory. **(F)** UMAP visualization of PRSS22 expression level in all epithelial cells. **(G, H)** Heatmap reveals the functional and pathway changes during the transition process.

In the malignant transformation process of colorectal cancer, the dynamic evolution of epithelial-derived cells plays a crucial biological role. This UMAP plot illustrates the gene expression patterns of Lineage3 lineage cells, with cell populations distributed according to their gene expression levels (**Figure 4A**). The colors range from blue to red, representing a gradient from low to high gene expression, reflecting the characteristics of cells at different stages of transformation (**Figure 4B**). The plot shows several cell subtypes, including E1, E2, E3, and E4, each of which plays a progressively changing role in the tumor progression. E1 cells represent the early stage of tumor development or normal cells, with low gene expression, typically in the early stages of development or differentiation. These cells might be tumor-initiating cells, exhibiting a more stable or less active state. E2 cells are in the middle phase of transformation, with an increase in gene expression and displaying some tumor characteristics. These cells could represent a key stage in the transition from normal to cancerous cells. E3 cells are in the later phase of tumor transformation, with higher gene expression, showing increased cell proliferation and invasive features, indicating further malignant progression of the tumor. These cells display strong adaptability, maintaining growth and expansion within the TME. E4 cells, located at the end of the transformation trajectory, have the highest gene expression levels, representing the late-stage tumor cells and the final phase of malignant transformation. While normal cells (NM) are not involved in cell trajectory differentiation. By analyzing these cell subtypes, we can observe that cells undergo different stages of differentiation and transformation during tumor progression, from early normal or early tumor cells to potentially late-stage malignant tumor cells, revealing the dynamic process of tumor progression from low to high malignancy.

Subsequently, we identified 45 high-variation genes by intersecting the results from differential analysis of E4 (100 genes) and pseudotime analysis (500 genes) (**Figure 4C**). These genes ranked highly in both analyses, demonstrating their consistency and significance across different methods. Next, we employed the Boruta algorithm of the 45 genes and identified 35 prognosis-related genes. Genes of the red box were rejected (**Figure 4D**, **Supplementary Table 1**). Notably, the *PRSS22* gene was among the top-ranked genes in all sets and its expression pattern was also consistent with the transition trajectory (**Figures 4E, F**), which led us to select it as the primary subject for further investigation. The detailed results of the differential expression analysis, pseudotime analysis were provided in Additional file 1-2.

The E4 cell population is primarily located in the C5 and C6 Clusters. GO function analysis revealed that its functions covered various biological processes particularly in tumor metastasis and immune regulation, such as wound healing, humoral immunity, MHC (**Figure 4G**). KEGG pathway analysis identified the coagulation response in the complement pathway and the NF-κB signaling pathway as key processes, especially playing an important role in the transformation trajectories of tumor progression (**Figure 4H**). The NF-κB signaling pathway, as one of the most famous immune related pathways, was closely related to tumor cell survival, proliferation, metastasis, and it also remodels the immunosuppressive microenvironment, promoting immune escape and resistance to immunotherapy (35–38), which also confirms our previous analysis. In the C5 Cluster, the gene expression pattern of E4 cells is enriched in key genes such as *C4BPB*, *CD55*, and *CEACAM1*. In the C6 Cluster, E4 cells exhibit significant antigen-presenting functions, especially with the high expression of MHC II-related genes (such as *HLA-DRB1* and *CD74*).

Based on these findings, we hypothesize that biological processes such as wound healing and the NF-κB signaling pathway play critical roles in promoting cancer cell progression, enhancing invasiveness, and modulating the immune microenvironment, especially during the malignant transformation of colorectal cancer. The impact of wound healing on tumor progression lies in the chronic involvement of various aspects of the healing process induced by inflammation and immune signals from tumor cells, disrupting this interaction and forming an uncontrolled positive feedback loop that profoundly impacts the spread of metastasis, immune surveillance, and proliferation, thereby promoting cancer progression. Equally important, cancer progression can occur through the loss of negative feedback control, such as in the apoptotic pathway and immune checkpoints, which means halting the regenerative pathways and resolving the wound healing process (39–41). Furthermore, the dysregulation of the NF-κB signaling pathway may provide conditions for immune escape which disrupted the immune microenvironment, thereby promoting further malignant transformation of the tumor (42, 43). Through these mechanisms, E4 cells may play a key role in the progression of tumors.

### 3.5 Biological function enrichment and immune infiltration analysis of *PRSS22*

**Figure 5:**
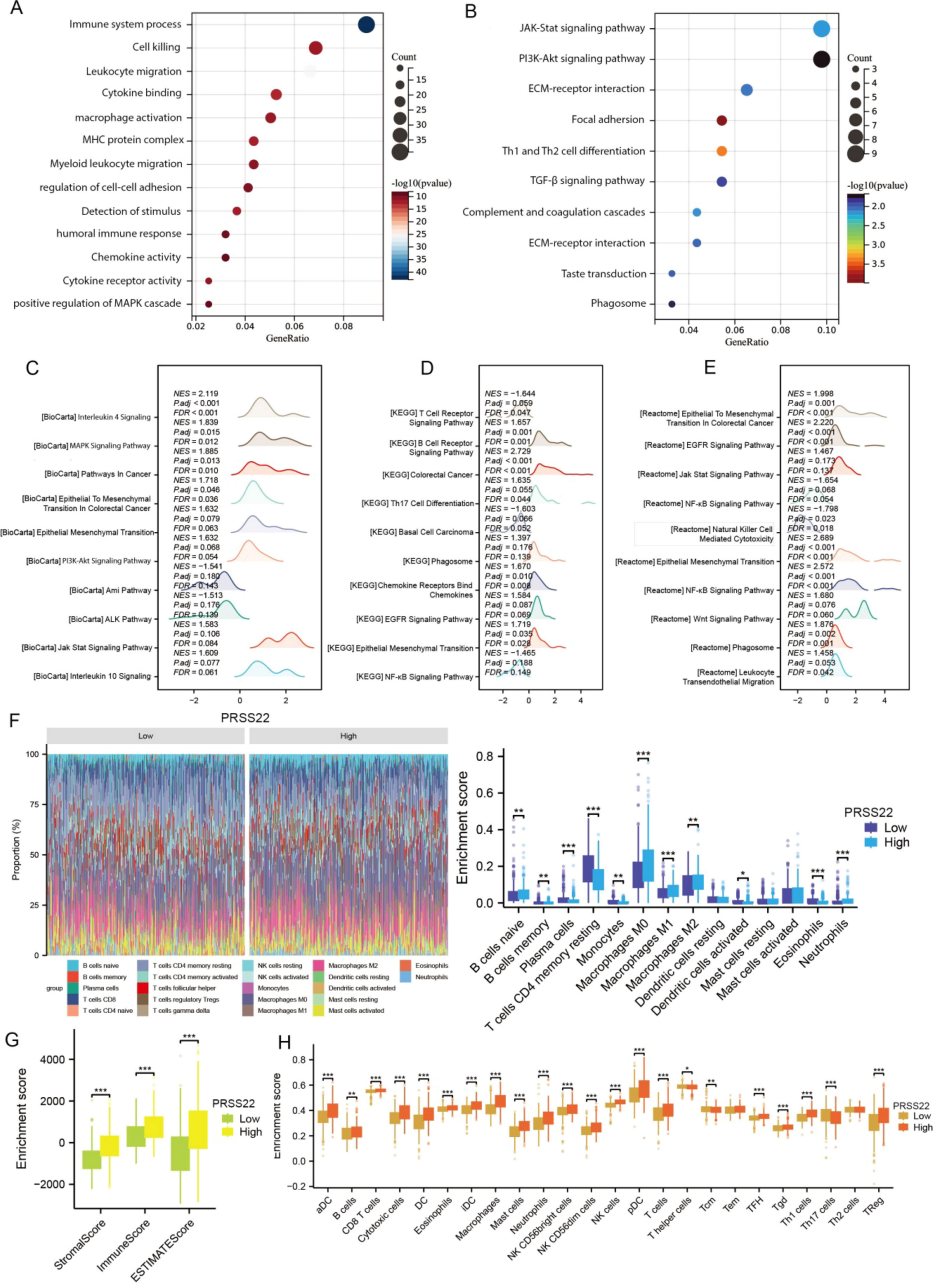
Functional enrichment analysis, immune status and tumor microenvironment of PRSS22. **(A, B)** GO and KEGG enrichment analyses of PRSS22. **(C-E)** GSEA analysis results of PRSS22 in Biocarat, KEGG, and Reactome databases. **(F-H)** Immune infiltration analysis of Cibersort, Estimate and ssGSEA of PRSS22. *, P<0.05, **, P<0.01, ***, P<0.001.

Based on the expression level of *PRSS22*, we divided the TCGA dataset into high-expression and low-expression groups. We then performed differential expression analysis between the two groups (log2FC > 1, adjusted p < 0.05). Subsequently, the top 100 upregulated and top 100 downregulated genes were combined for GO and KEGG enrichment analyses (**Figures 5A, B**). The results showed that the differentially expressed genes were primarily enriched in biological processes related to immune system processes, cell killing, macrophage activation, as well as pathways such as the JAK-STAT signaling pathway, PI3K-AKT signaling pathway, and focal adhesion. Furthermore, we performed GSEA enrichment analysis on the differential genes using the KEGG, Biocarta, and Reactome databases (**Figures 5C-E**). The analysis revealed that the genes were mainly enriched in interleukin-related pathways, epithelial–mesenchymal transition, PI3K-AKT signaling, and EGFR signaling pathways, with an overall upregulation trend in these pathways. In contrast, pathways such as NF-κB signaling showed a downregulation trend. This also suggests that PRSS22 may promote cell proliferation, migration, and immune microenvironment dysregulation through these pathways, which is consistent with our previous analysis. Finally, we analyzed immune infiltration in the tumor microenvironment using the CIBERSORT, ESTIMATE, and ssGSEA algorithms (**Figures 5F-H**). The results revealed significant differences in the infiltration of various immune cells, including B cells, macrophages, and dendritic cells, between the high and low *PRSS22* expression groups, suggesting distinct immune microenvironment profiles. Additionally, the high-expression group exhibited significantly higher stromal scores, immune scores, and ESTIMATE scores compared to the low-expression group, indicating lower tumor purity and a potentially poorer prognosis (17).

### 3.6 Upregulation of *PRSS22* and its correlation with clinical parameters

**Figure 6:**
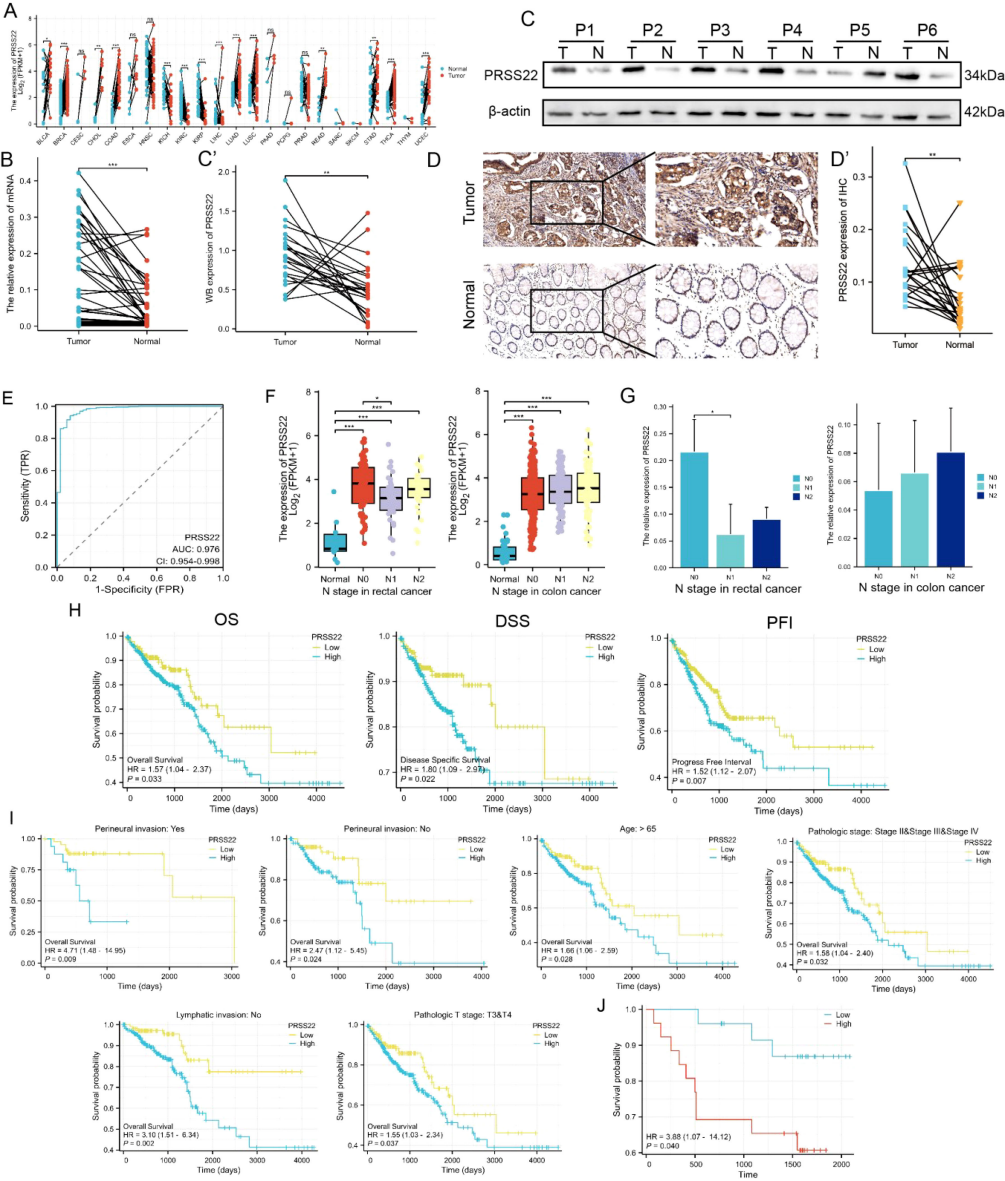
Upregulation of PRSS22 and its clinical significance. **(A)** The expression of PRSS22 in pan-cancer from TCGA database. **(B-D)** The expression of PRSS22 in CRC cancer tissue was detected by RT-qPCR (n=55), western blotting (n=24) and IHC (n=25). **(E)** The ROC curve of PRSS22. **(F)** The correlation between PRSS22 expression and the N stage of colon and rectal patients. **(G)** Expression levels of PRSS22 in colon (n=20) and rectal (n=35) patients of different N stages at Yangpu Central Hospital. **(H)** OS, DSS and PFI in TCGA cohort. **(I)** OS were worse in subgroups of CRC with higher PRSS22 expression. **(J)** Survival analysis of PRSS22 in 55 patients with CRC from Yangpu hospital. CRC, colorectal cancer; OS, overall survival; DSS, disease specific survival; PFI, progression free interval; IHC, immunohistochemistry. *, P<0.05, **, P<0.01, ***, P<0.001.

The expression of *PRSS22* in pan-cancer from TCGA database is shown in **Figure 6A**. The results showed that *PRSS22* was upregulated in breast invasive carcinoma (BRCA), colon adenocarcinoma (COAD), lung adenocarcinoma (LUAD), lung squamous cell carcinoma (LUSC), rectum adenocarcinoma (READ), uterine corpus endometrial carcinoma (UCEC), and thyroid carcinoma (THCA), while it was downregulated in kidney chromophobe (KICH), kidney renal clear cell carcinoma (KIRC), kidney renal papillary cell carcinoma (KIRP), and liver hepatocellular carcinoma (LIHC). This suggests the abnormal expression of *PRSS22* in various types of cancer. Subsequently, we selected 55 pairs of clinical samples for RT-qPCR (**Figure 6B**), Western blotting (**Figure 6C**, **Supplementary Figure 3**) and IHC (**Figure 6D**), which confirmed the upregulation of *PRSS22* expression in cancer tissues at both the mRNA and protein levels. *PRSS22* might serve as a potential diagnostic biomarker, as indicated by an AUC of 0.976 (**Figure 6E**). It was worth noting that there was a correlation between *PRSS22* expression and the N stage only in rectal cancer (**Figure 6F**), which has also been confirmed in the cohort of our Yangpu Hospital (**Figure 6G**). Then, Kaplan–Meier curve analysis showed that *PRSS22* expression was associated with OS (overall survival), DSS (Disease Specific Survival), PFI (Progression free interval) in CRC patients (**Figure 6H**). In subgroups with and without peripheral nerve invasion, age > 65 years, pathological grades II&III&IV, no lymph node metastasis, and T3&T4 staging, increased *PRSS22* expression was associated with poor overall survival (**Figure 6I**). Finally, Kaplan-Meier analysis showed that the patients in Yangpu hospital with high *PRSS22* expression had poorer overall survival than those with low *PRSS22* expression (n=55) (**Figure 6J**).

### 3.7 *PRSS22* Knockdown inhibits cell migration and proliferation in CRC

**Figure 7:**
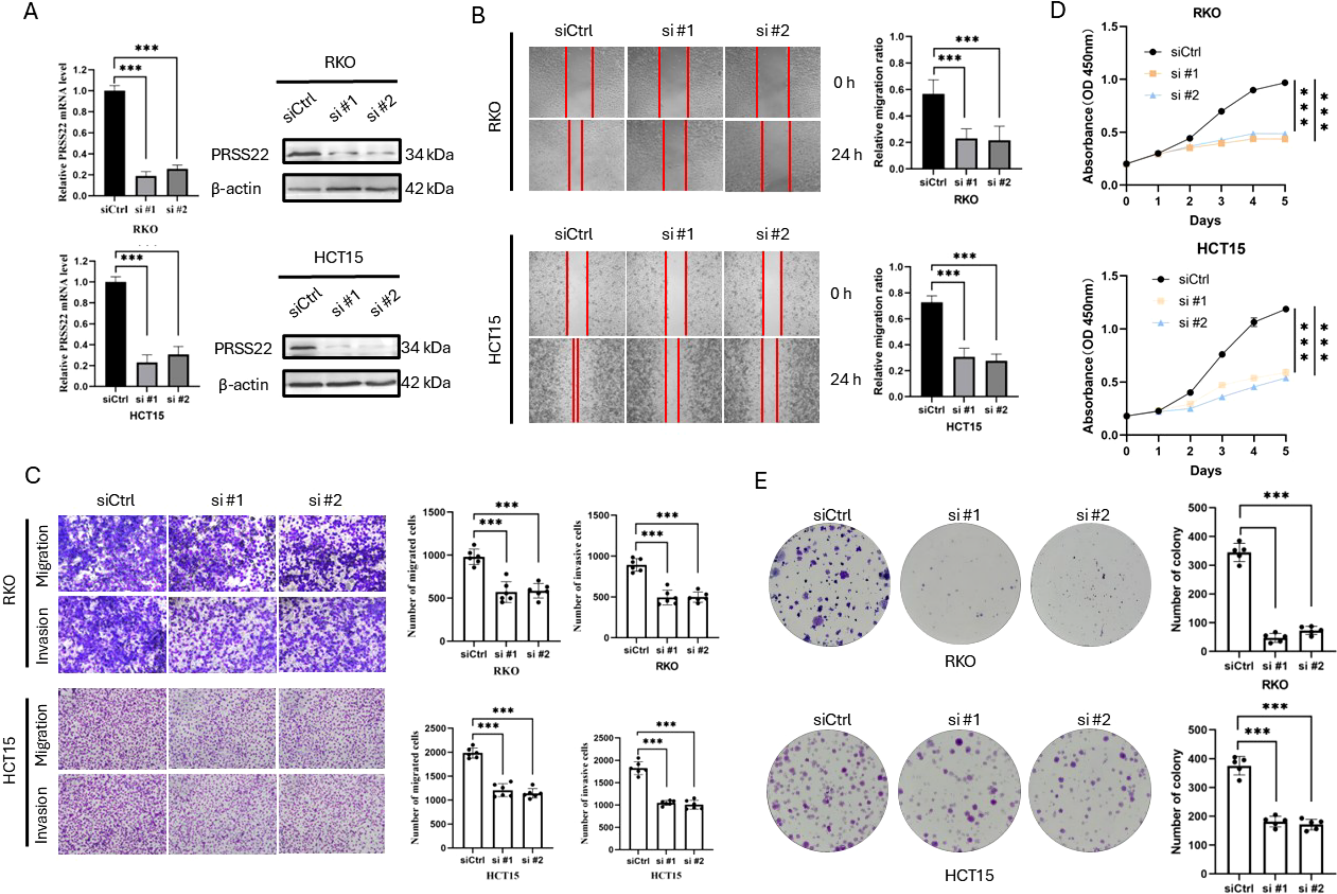
Knockdown of PRSS22 Inhibits Migration, Invasion, and Proliferation in CRC. **(A)** RT-qPCR and western blotting were used to determine the expression of PRSS22 in RKO and HCT15 transfected with siRNA. **(B)** Wound healing assay to evaluate the migration of RKO and HCT15 cells with or without PRSS22 knockdown. **(C)** Transwell assays assessing migration and invasion of RKO and HCT15 cells with or without PRSS22 knockdown. **(D, E)** CCK-8, Colony formation assays were used to assess cell viability of RKO and HCT15 cells with or without PRSS22 knockdown. ***, P<0.001.

In order to explore the functions of *PRSS22* in CRC, *PRSS22* was knocked down by siRNA in HCT15 and RKO, and the efficiency was verified by RT-qPCR and western blotting (**Figure 7A**). The wound healing assay showed a marked decrease in cell migration following *PRSS22* knockdown (**Figure 7B**). Consistent with the results, transwell assays verified that *PRSS22* knockdown inhibited RKO and HCT15 cells invasion and migration (**Figure 7C**). In addition, we detected the viability of RKO and HCT15 with *PRSS22* knockdown, the results showed that *PRSS22* knockdown significantly reduced the ability of proliferation and colony formation (**Figures 7D, E**).

## 4 Discussion

This study employs single-cell RNA sequencing (scRNA-seq) and bioinformatics analysis to reveal the heterogeneity of colorectal cancer (CRC) epithelial cells and their critical role in tumor progression. We identified multiple epithelial cell subpopulations and explored the expression pattern of *PRSS22*, a highly expressed gene in E4 cells, along with its potential role in CRC. The heterogeneity of colorectal cancer cells is a key feature of tumor progression.

The heterogeneity of CRC cells plays a crucial role in tumor progression. Through UMAP dimensionality reduction and clustering analysis, we classified epithelial cells into eight subpopulations, including four cancerous epithelial subpopulations (E1–E4) and normal epithelial cells (NM). CNV analysis revealed significant differences between the four cancerous epithelial subpopulations (E1–E4) and normal epithelial cells (NM). Among them, the CNV levels of E1–E4 were generally higher than those of NM, with the highest levels observed in E4, suggesting that E4 may represent a highly invasive cancer cell subpopulation. Our findings are consistent with the study by Soulafa Mamlouk et al., which demonstrated through three-dimensional morphological-molecular reconstruction that DNA copy number variation (CNV) is a major source of CRC tumor heterogeneity, with different clusters exhibiting distinct CNV aberrations along the proximal-distal axis (44). In our study, tumor cell gene expression analysis showed that different clusters were associated with protein synthesis (C1), stress response (C2), immune response (C4), and barrier function (C5), with the E4 subpopulation enriched for immune evasion genes and exhibiting strong invasiveness. Further cell proportion analysis revealed that E2 was more prevalent in adjacent non-tumor tissues, whereas E1, E3, and E4 were primarily enriched in tumor tissues, with E4 being a tumor-specific subpopulation.

Moreover, tumor heterogeneity is not only reflected in gene expression differences but is also closely related to prognosis and intercellular interactions. Survival analysis revealed cancerous epithelial marker genes, particularly those in E4, were linked to poorer survival outcomes. Cell communication analysis revealed that E2–E4 tumor cells exhibited enhanced interactions with other cells, with E4 demonstrating the strongest signal input-output capacity. At the same time, The E4 subpopulation exhibits unique ligand-receptor interactions compared to other subgroups, such as the NAMPT ligand-receptor interaction with T cells and the EGFR ligand-receptor interaction with stromal cells. NAMPT exhibits anti-apoptotic properties and is highly expressed across various tumor types. Moreover, in advanced and metastatic tumors, the proportion of tumors with high NAMPT levels is also significantly elevated. Additionally, elevated NAMPT expression is associated with poor patient prognosis but is independent of tumor staging (45, 46). Additionally, NAMPT has been shown to be associated with dysregulation of the tumor immune microenvironment, including immune surveillance evasion, immunosuppression, and immune tolerance (24, 25).

Cancer cells recruit supportive stromal cells from the surrounding endogenous tissue stroma to promote tumor formation. Consequently, stromal cells constitute a crucial component of the tumor microenvironment (47–49). Studies have shown that in colorectal cancer, subtypes with higher stromal infiltration exhibit poorer prognosis and distinct immune escape mechanisms (50). Our results suggest that the E4 subpopulation may promote tumor progression through EGFR ligand-receptor interactions with stromal cells, potentially facilitating tumor immune evasion. Our findings align with the study by Yihao Mao et al., which classified CRC samples into three immune phenotypes with distinct TME cell infiltration patterns and immune evasion mechanisms (50).

Additionally, CCL20 and CXCL8 secreted by tumor cells into the tumor microenvironment (TME) were found to promote the recruitment of Treg cells and reduce CD8+ T cell infiltration, thereby facilitating tumor immune evasion (51, 52). Our study further demonstrates that the E4 subpopulation promotes tumor progression and evades immune surveillance by upregulating immune-related genes such as CXCL8, consistent with previous research findings. These findings highlight the differential immune evasion mechanisms among subpopulations and their profound implications for tumor treatment response.

Our pseudotime analysis revealed E4 cells, located at the end of the transformation trajectory, had the highest gene expression levels, representing the late-stage tumor cells and the final phase of malignant transformation, which was consistent with our previous analysis. The gene expression pattern of E4 indicates its pivotal role in tumor progression, particularly through pathways associated with wound healing and NF-κB signaling. These findings reveal the dynamic evolution of colorectal cancer from early tumor cells (E1) to late-stage malignant cells (E4), emphasizing the critical role of metastasis and immune dysregulation in tumor progression (42, 43, 53).

Finally, we validated the high expression pattern of the key gene *PRSS22* in the E4 subpopulation and its association with poor prognosis using public databases and clinical samples. Cell experiments further confirmed that *PRSS22* promotes cell proliferation and metastasis, which is consistent with our previous analysis of the E4 subpopulation. Notably, both the TCGA cohort and our Yangpu District Hospital cohort showed differential *PRSS22* expression across different N stages in colon and rectal cancer, suggesting potential heterogeneity between these two similar cancers.

Previously, only a few studies have explored the association between *PRSS22* and cancer. In breast and liver cancer, *PRSS22* is reported to promote tumor progression through the ERK signaling pathway (15, 54). Recent literature has reported that *PRSS22* may serve as a biomarker for early diagnosis and dynamic monitoring in ulcerative colitis (55). In colorectal cancer, it is only known that *PRSS22* may exhibit differential mRNA expression levels in the blood of colorectal cancer patients compared to normal controls (56).

In conclusion, this study provides an in-depth characterization of the heterogeneity of colorectal cancer epithelial cells, highlighting the critical role of the E4 subpopulation in tumor progression, and immune evasion and prognosis. The high expression of *PRSS22* is not only associated with CRC prognosis but also presents a potential target for developing tumor biomarkers. By utilizing single-cell transcriptomic analysis, we have laid the foundation for further understanding the dynamic changes within the tumor microenvironment. PRSS22 enrichment in E1 and E4 cells, not all epithelial cancer cells, suggests it may not have good therapeutic effects as a single target. Future studies should continue exploring the mechanisms underlying CRC cell heterogeneity and its interactions with the tumor microenvironment, particularly focusing on immune evasion dysregulation mechanisms in the E4 subpopulation, with the goal of identifying novel therapeutic targets for tumor immunotherapy. Additionally, interventions targeting *PRSS22* and its downstream pathways may open new avenues for personalized treatment strategies, promoting advances in early diagnosis and precision therapy for colorectal cancer. Although we have explored the changes in the colorectal tumor microenvironment from computational information, clinical samples, and cellular experiments, there are still some limitations. For example, the sample size of the single-cell dataset needs to be expanded, and the number of tumor clinical samples also requires further increase.

In conclusion, this study provides new insights into cancer progression and metastasis. We identified specific cell clusters associated with high metastatic potential and immune heterogeneity, and we jointly confirmed their role in the tumor immune microenvironment at both the bulk transcriptomic and single-cell transcriptomic levels. Its marker, PRSS22, shows significant potential as a novel immunotherapeutic target for CRC.

## Data Availability

The original contributions presented in the study are included in the article/**Supplementary Material**. Further inquiries can be directed to the corresponding authors.
