# Supplementary material for: Single-cell transcriptomics in colorectal cancer uncover the potential of metastasis and immune dysregulation of a cell cluster overexpressed PRSS22
Source: Front Immunol. 2025 May 20;16:1586428. doi: 10.3389/fimmu.2025.1586428 (PMC12130013; doi:10.3389/fimmu.2025.1586428)
Supplement: Supplementary file 3 [file DataSheet3.zip › original data/Figure 5/method R package.docx]

**R (4.2.1) version；ggplot2[3.4.4] was used for visualization of all images**

Figure 5A-C：

R package: stats[4.2.1], car[3.1-0]

Figure 5D

R package: pROC[1.18.0],

Figure 5E-F

R package: stats[4.2.1], car[3.1-0]

Figure 5G-I

R package: survival[3.3.1], survminer[0.4.9]
