# Supplementary figures and images for: Single-cell transcriptomics in colorectal cancer uncover the potential of metastasis and immune dysregulation of a cell cluster overexpressed PRSS22
Source: Front Immunol. 2025 May 20;16:1586428. doi: 10.3389/fimmu.2025.1586428 (PMC12130013; doi:10.3389/fimmu.2025.1586428)

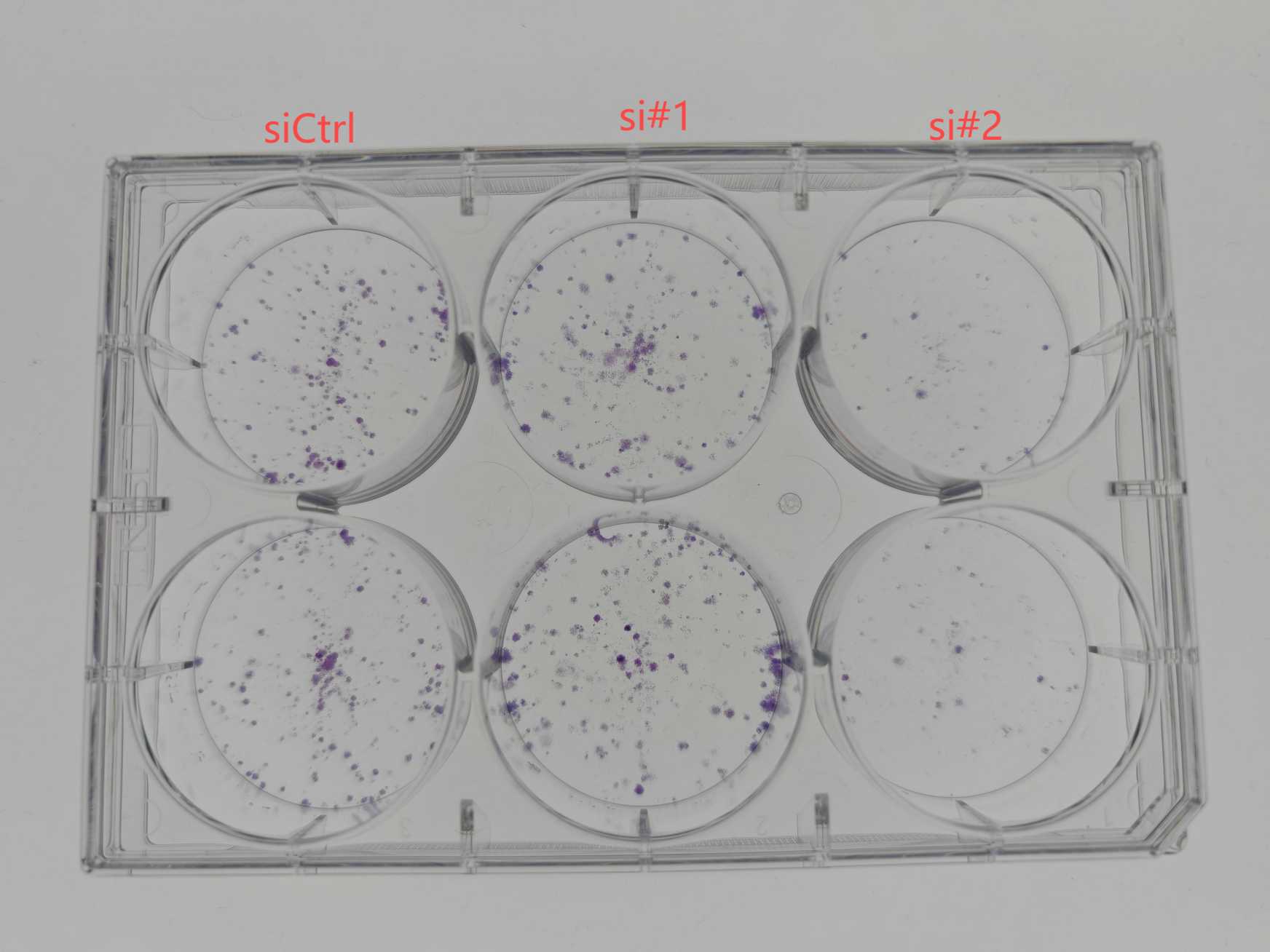

Supplement: Supplementary file 3 [file DataSheet3.zip › original data/Figure 6(Cell experiment)/HCT15 colony formation/1.jpg]

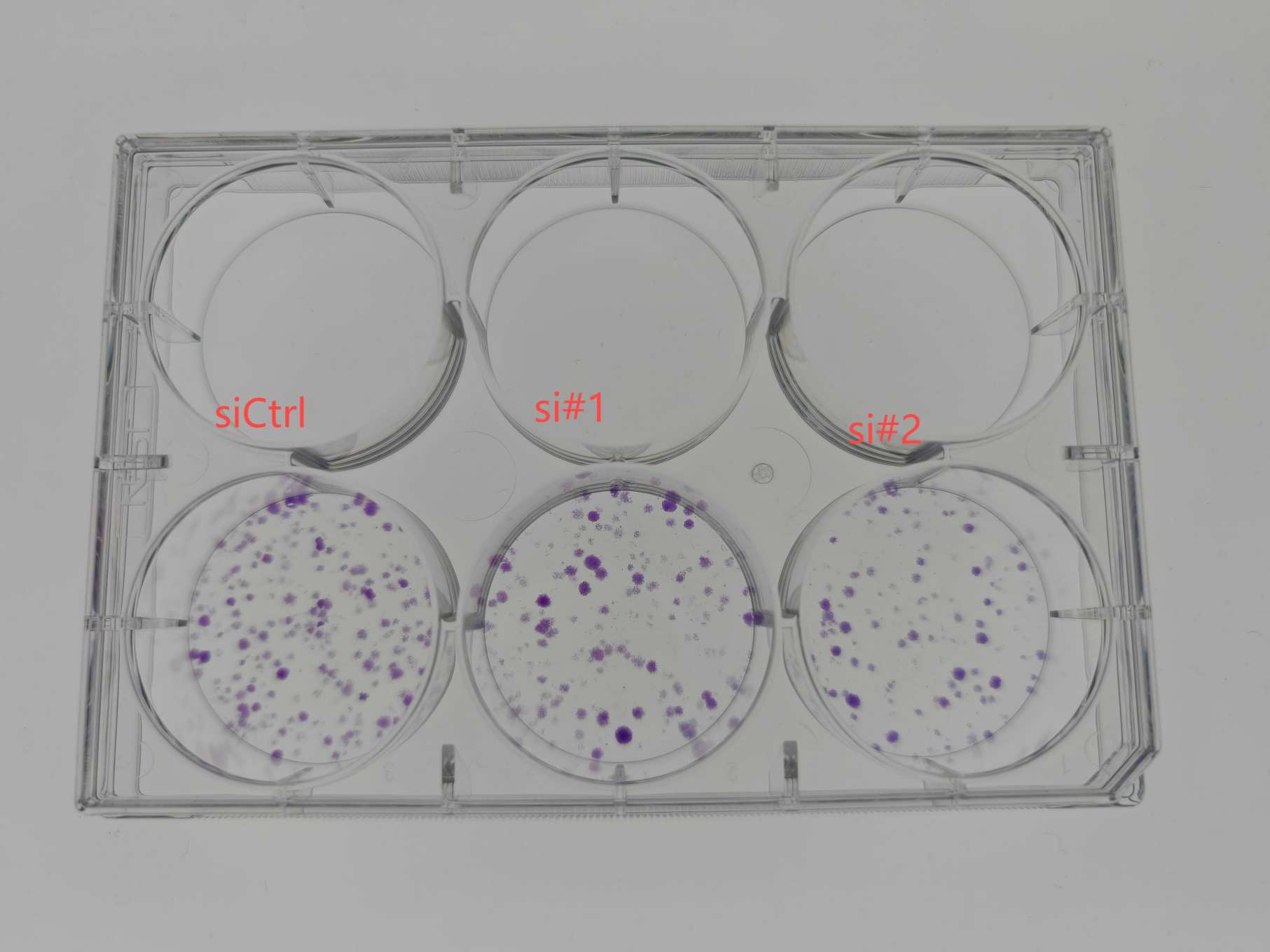

Supplement: Supplementary file 3 [file DataSheet3.zip › original data/Figure 6(Cell experiment)/HCT15 colony formation/2.jpg]

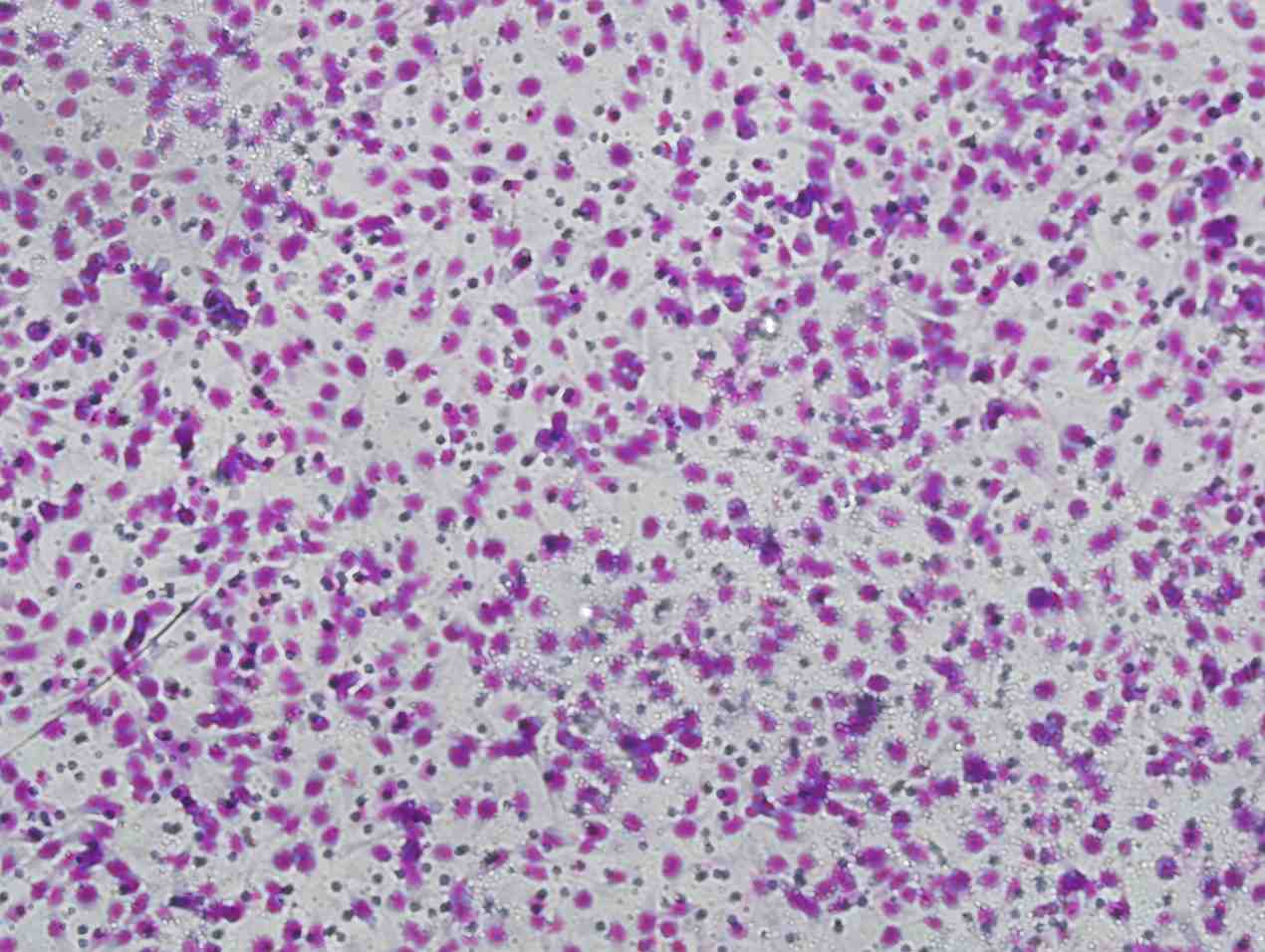

Supplement: Supplementary file 3 [file DataSheet3.zip › original data/Figure 6(Cell experiment)/HCT15 transwell/invasion/si#1 -1.jpg]

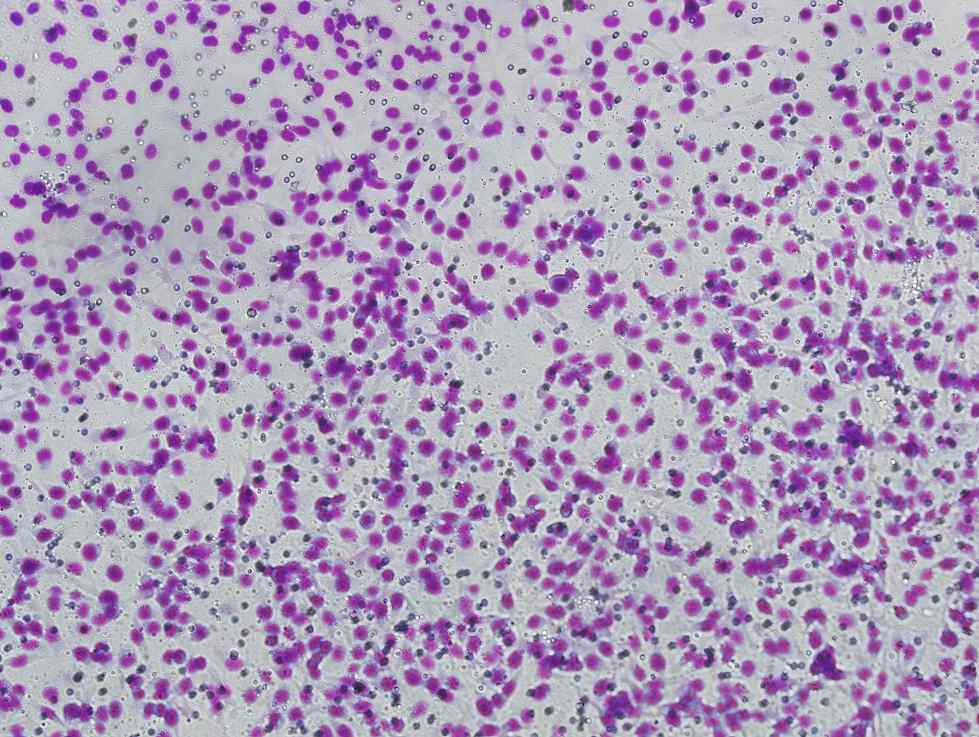

Supplement: Supplementary file 3 [file DataSheet3.zip › original data/Figure 6(Cell experiment)/HCT15 transwell/invasion/si#1 -2.jpg]

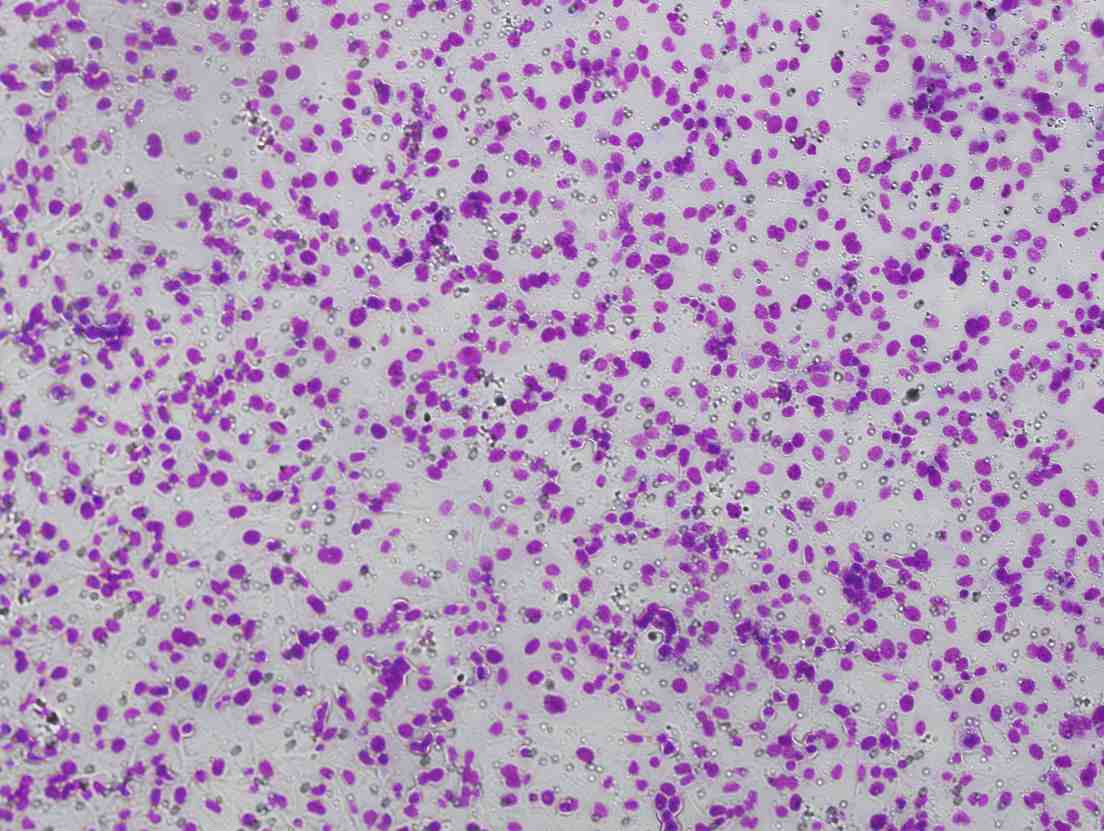

Supplement: Supplementary file 3 [file DataSheet3.zip › original data/Figure 6(Cell experiment)/HCT15 transwell/invasion/si#1 -3.jpg]

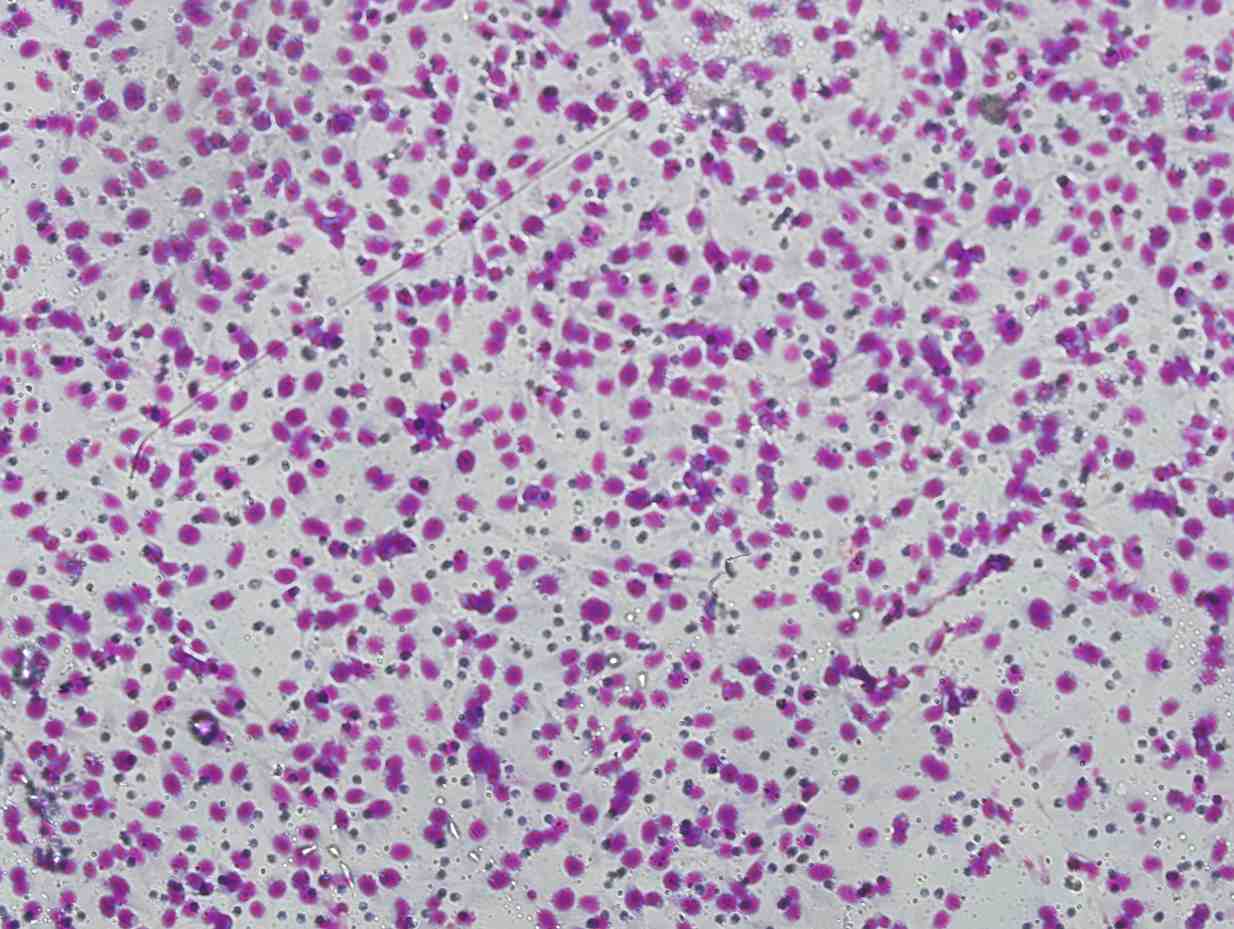

Supplement: Supplementary file 3 [file DataSheet3.zip › original data/Figure 6(Cell experiment)/HCT15 transwell/invasion/si#2 -1.jpg]

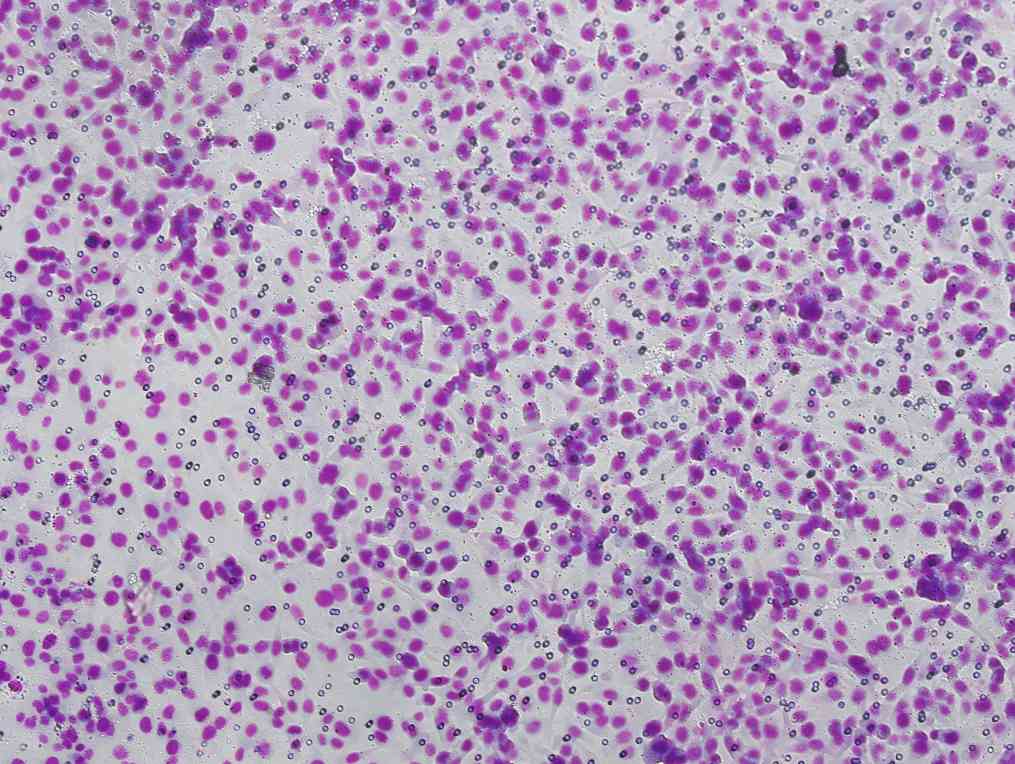

Supplement: Supplementary file 3 [file DataSheet3.zip › original data/Figure 6(Cell experiment)/HCT15 transwell/invasion/si#2 -2.jpg]

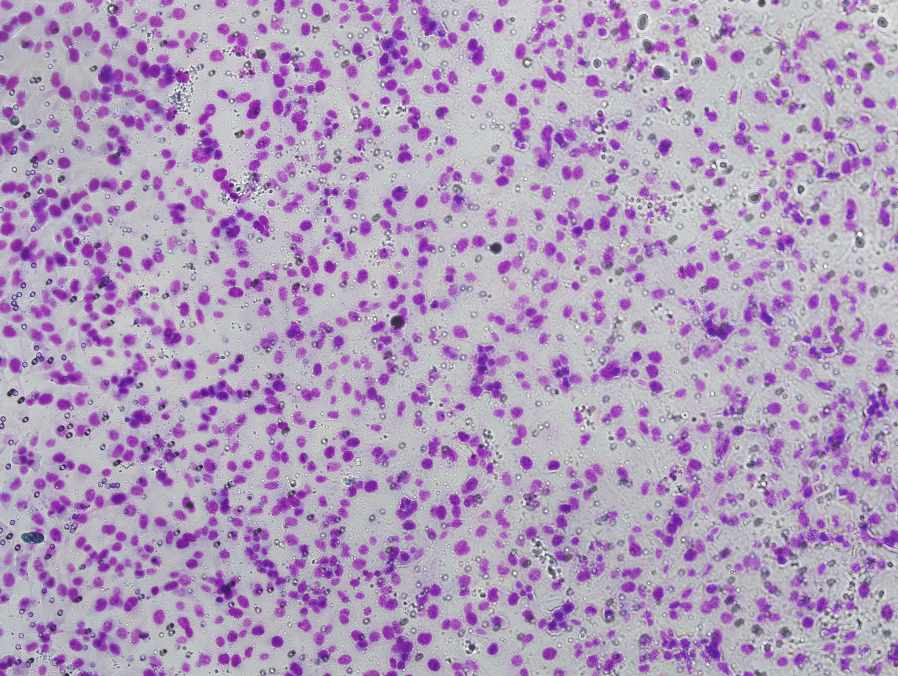

Supplement: Supplementary file 3 [file DataSheet3.zip › original data/Figure 6(Cell experiment)/HCT15 transwell/invasion/si#2 -3.jpg]

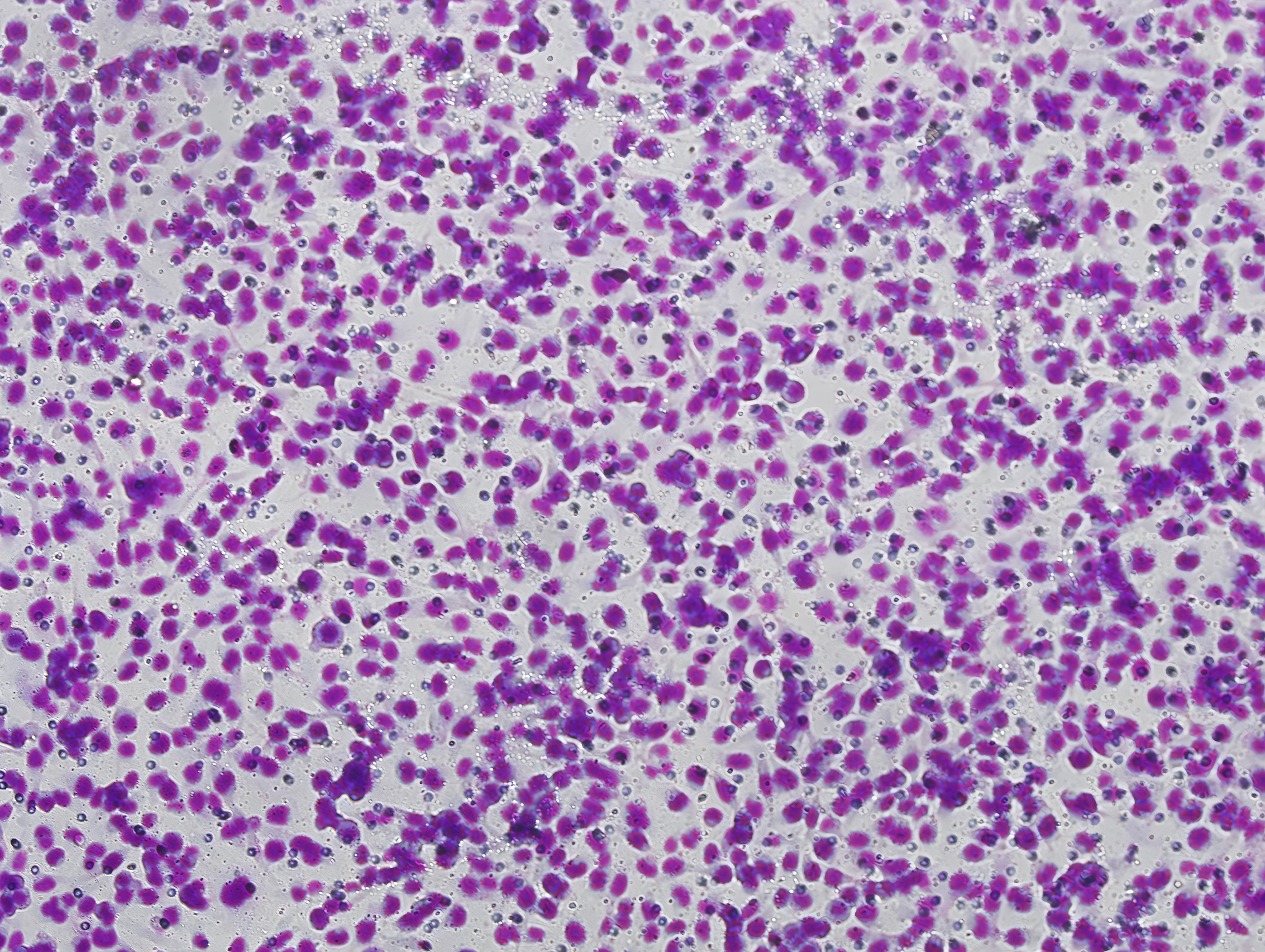

Supplement: Supplementary file 3 [file DataSheet3.zip › original data/Figure 6(Cell experiment)/HCT15 transwell/invasion/siCtrl -1.jpg]

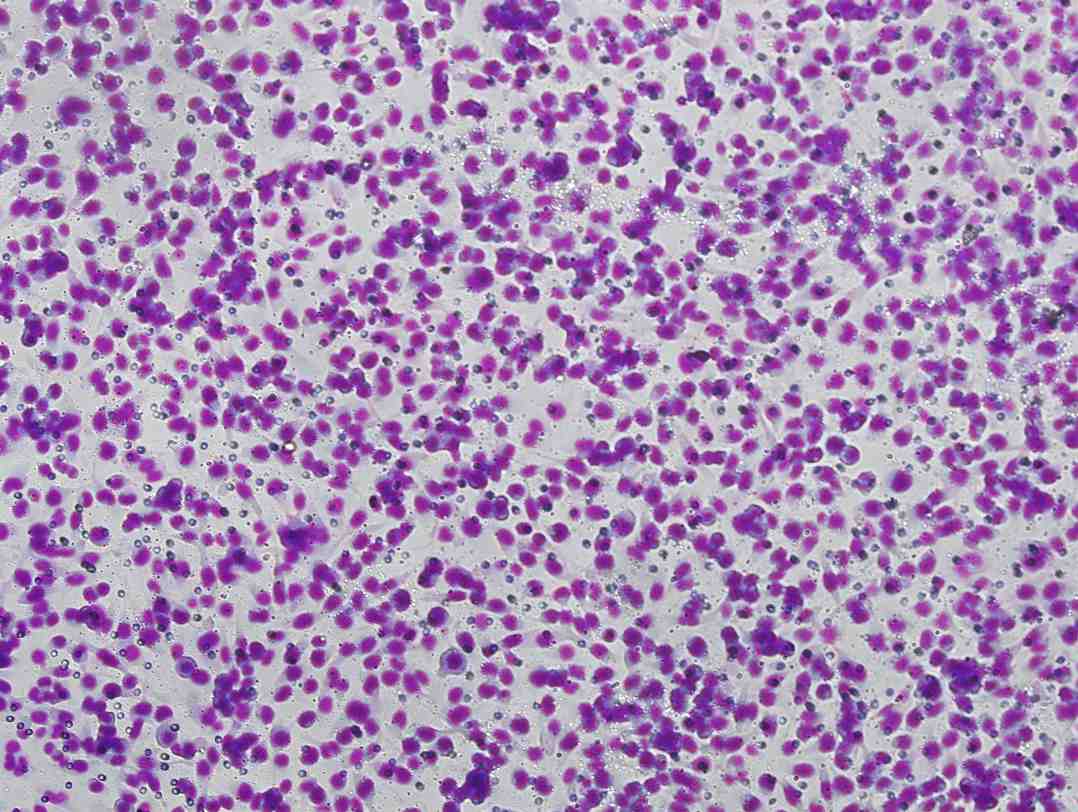

Supplement: Supplementary file 3 [file DataSheet3.zip › original data/Figure 6(Cell experiment)/HCT15 transwell/invasion/siCtrl -2.jpg]

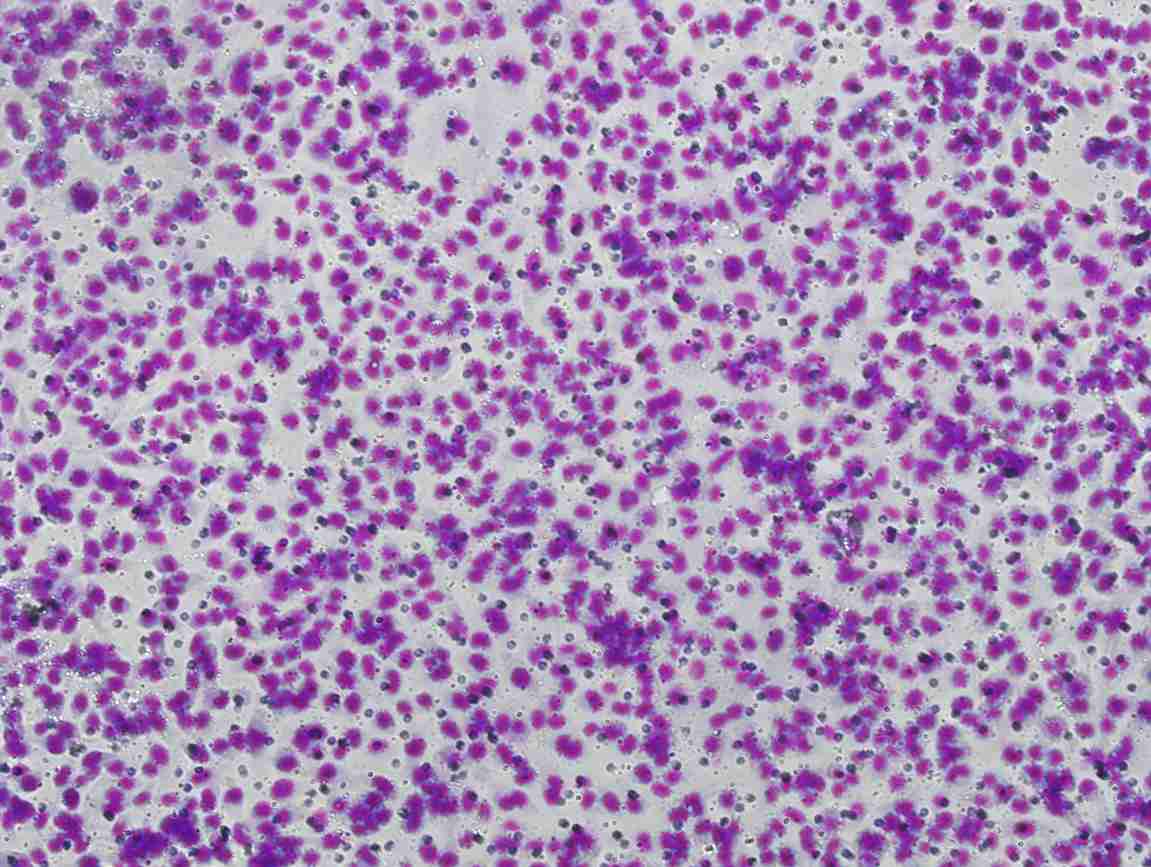

Supplement: Supplementary file 3 [file DataSheet3.zip › original data/Figure 6(Cell experiment)/HCT15 transwell/invasion/siCtrl -3.jpg]

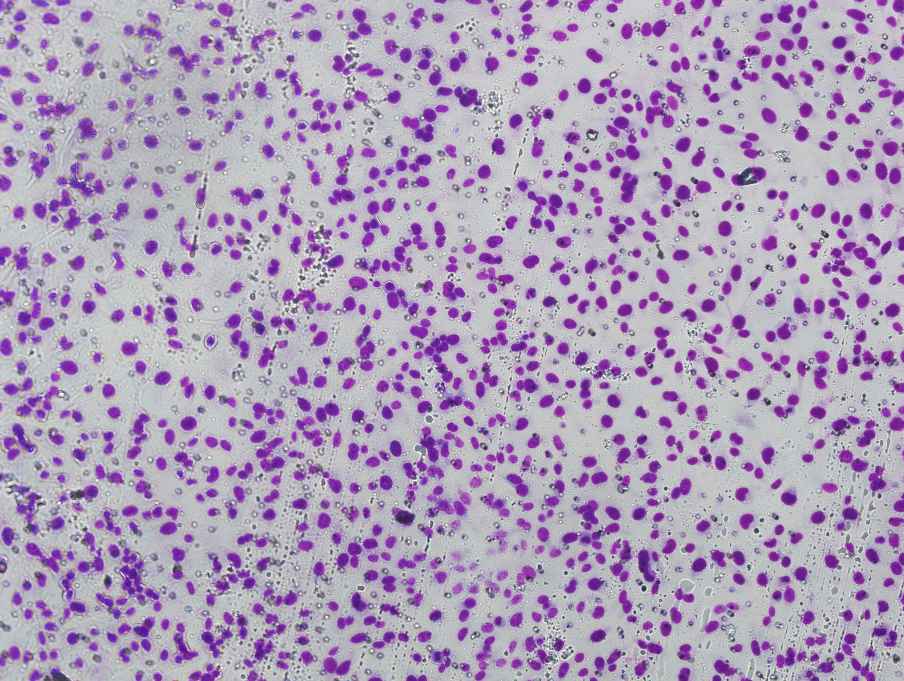

Supplement: Supplementary file 3 [file DataSheet3.zip › original data/Figure 6(Cell experiment)/HCT15 transwell/Migration/si#1 -1.jpg]

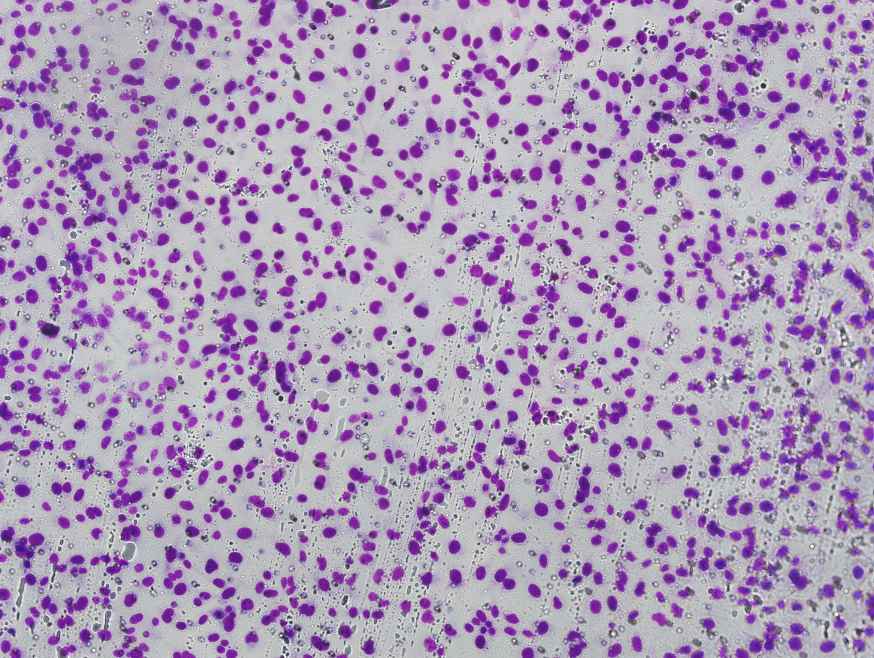

Supplement: Supplementary file 3 [file DataSheet3.zip › original data/Figure 6(Cell experiment)/HCT15 transwell/Migration/si#1 -2.jpg]

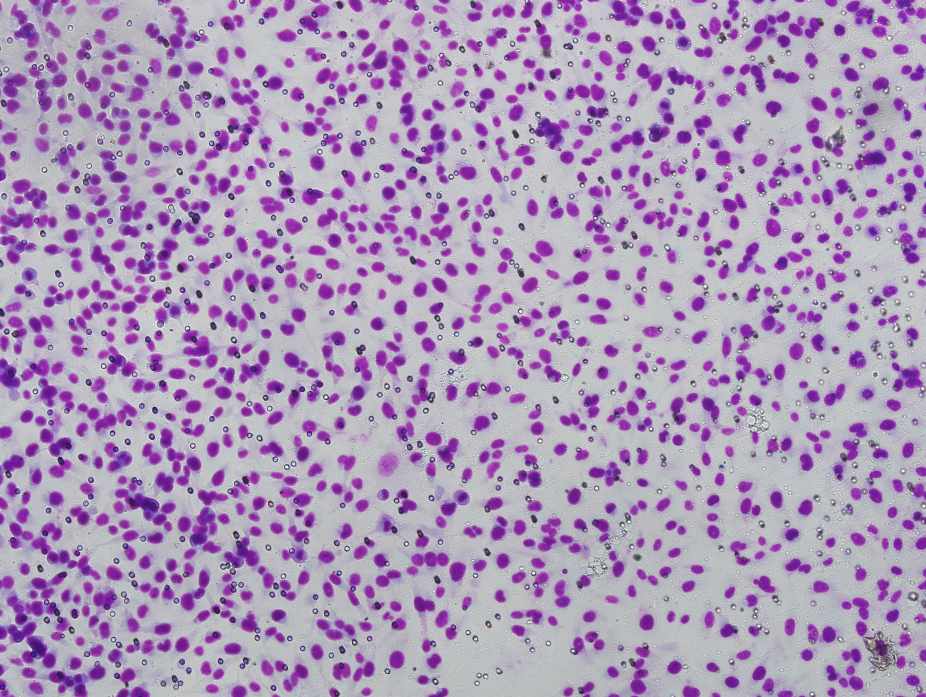

Supplement: Supplementary file 3 [file DataSheet3.zip › original data/Figure 6(Cell experiment)/HCT15 transwell/Migration/si#1 -3.jpg]

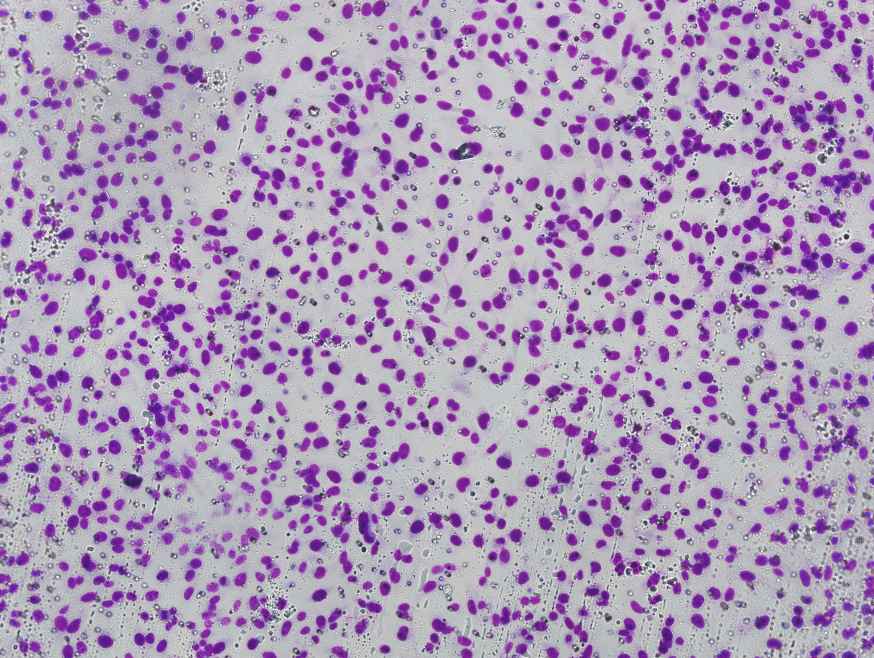

Supplement: Supplementary file 3 [file DataSheet3.zip › original data/Figure 6(Cell experiment)/HCT15 transwell/Migration/si#2 -1.jpg]

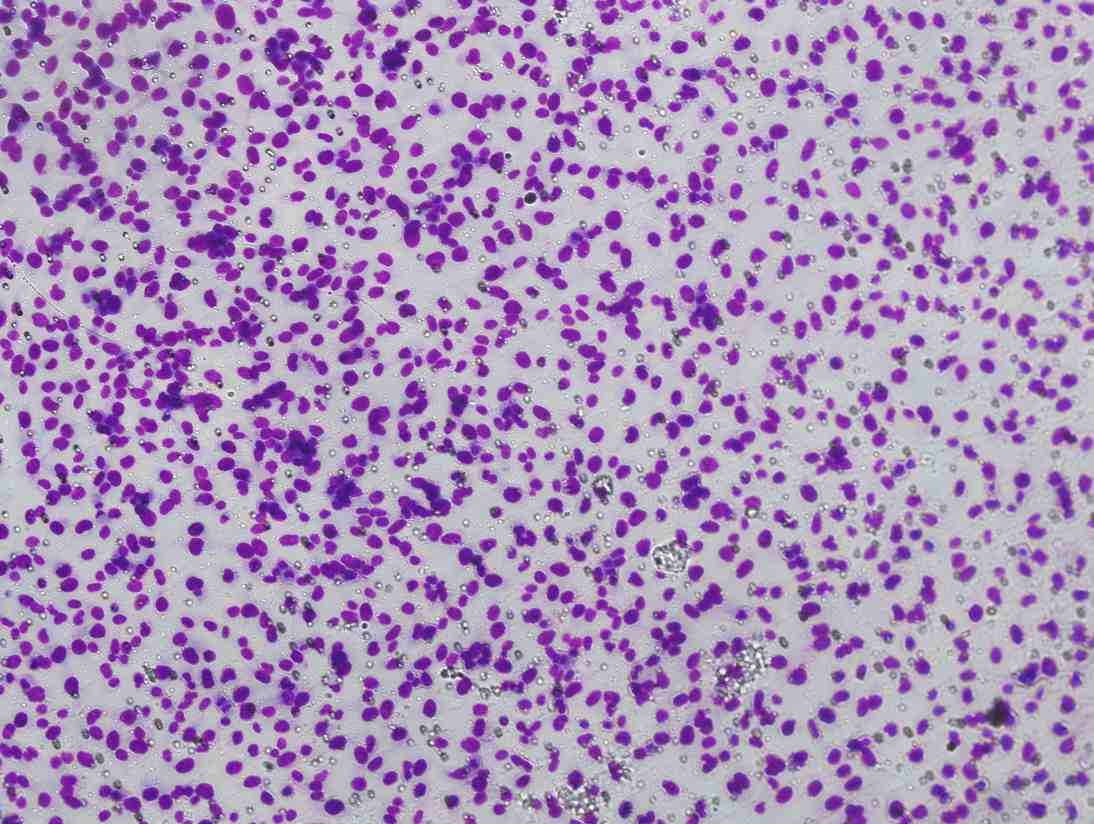

Supplement: Supplementary file 3 [file DataSheet3.zip › original data/Figure 6(Cell experiment)/HCT15 transwell/Migration/si#2 -2.jpg]

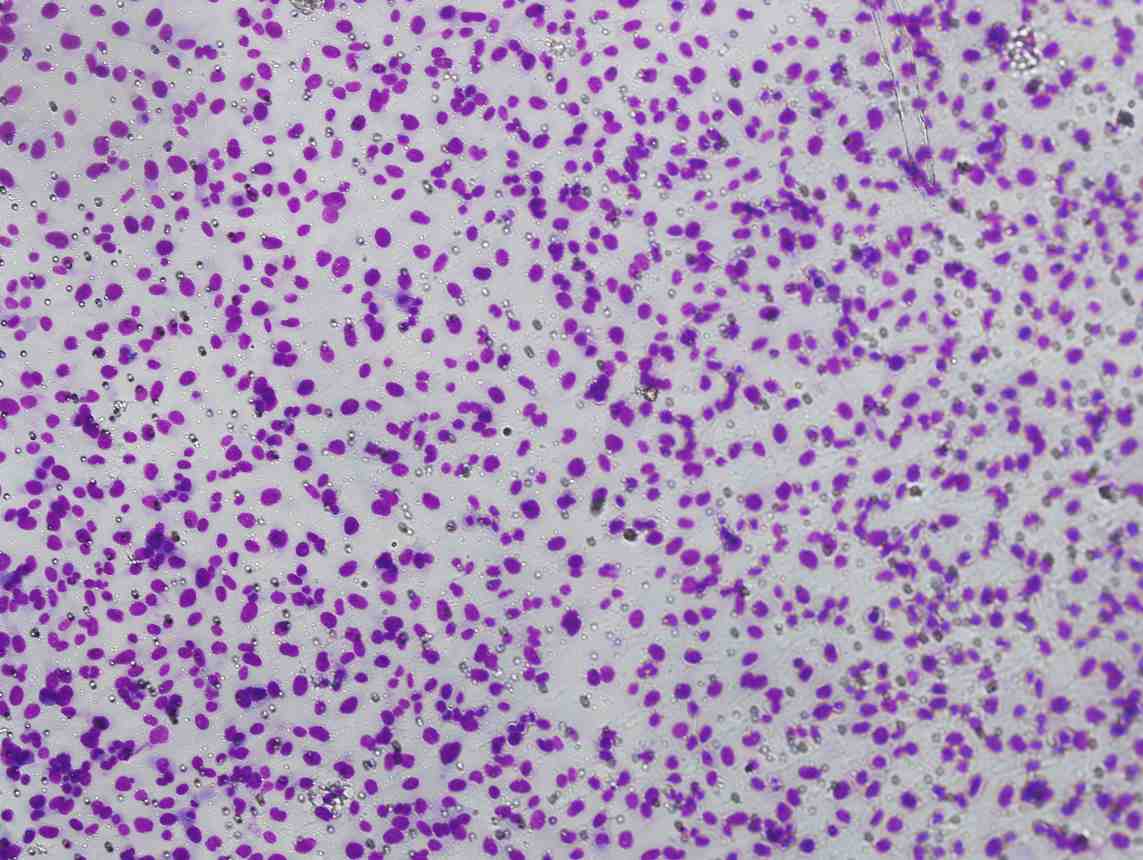

Supplement: Supplementary file 3 [file DataSheet3.zip › original data/Figure 6(Cell experiment)/HCT15 transwell/Migration/si#2 -3.jpg]

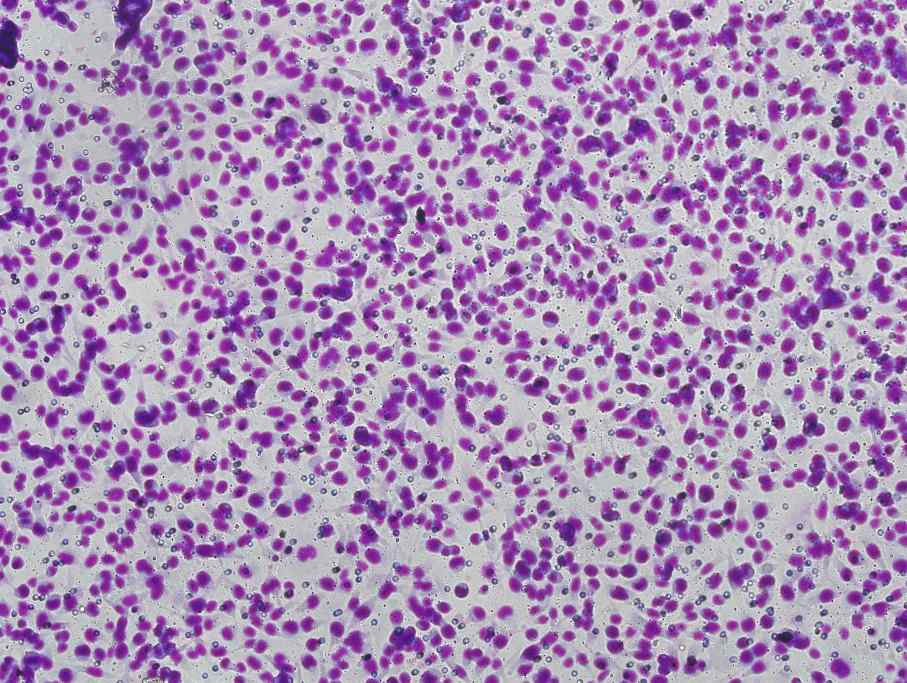

Supplement: Supplementary file 3 [file DataSheet3.zip › original data/Figure 6(Cell experiment)/HCT15 transwell/Migration/siCtrl -1.jpg]

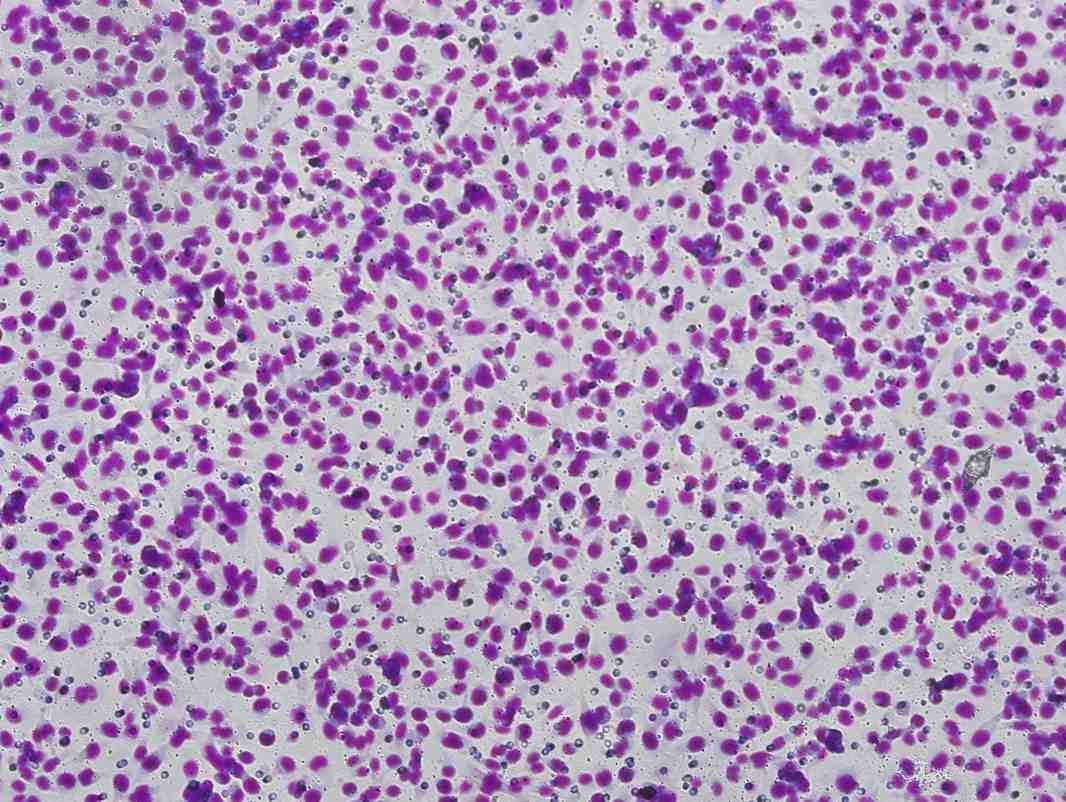

Supplement: Supplementary file 3 [file DataSheet3.zip › original data/Figure 6(Cell experiment)/HCT15 transwell/Migration/siCtrl -2.jpg]

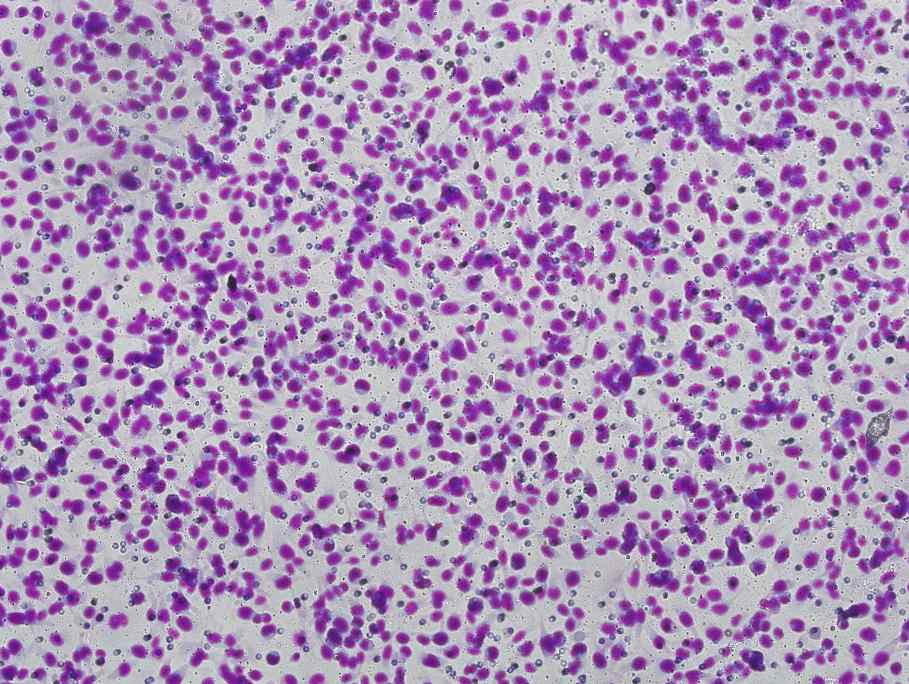

Supplement: Supplementary file 3 [file DataSheet3.zip › original data/Figure 6(Cell experiment)/HCT15 transwell/Migration/siCtrl -3.jpg]

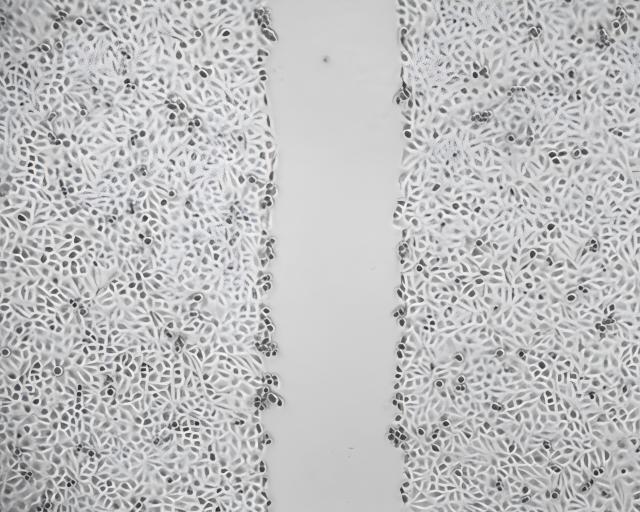

Supplement: Supplementary file 3 [file DataSheet3.zip › original data/Figure 6(Cell experiment)/HCT15 wound healing/0h/si#1 -1.jpg]

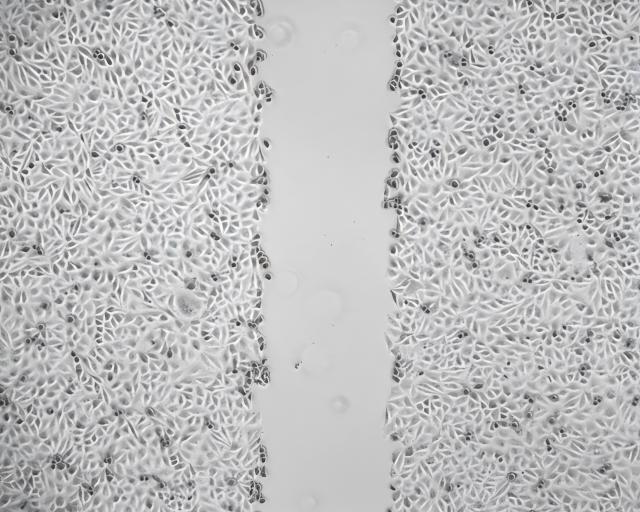

Supplement: Supplementary file 3 [file DataSheet3.zip › original data/Figure 6(Cell experiment)/HCT15 wound healing/0h/si#1 -2.jpg]

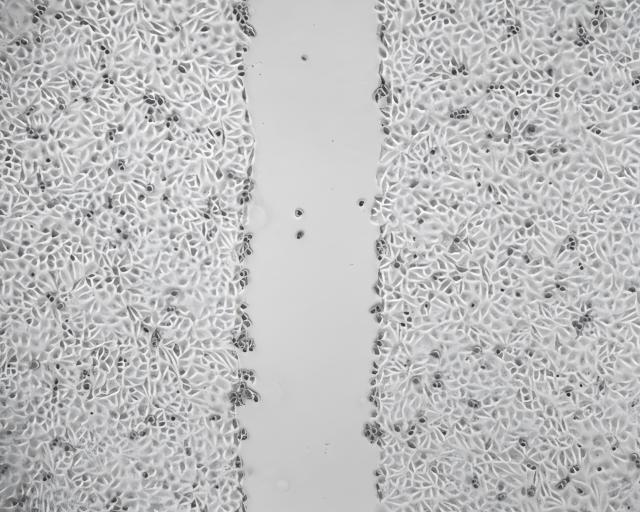

Supplement: Supplementary file 3 [file DataSheet3.zip › original data/Figure 6(Cell experiment)/HCT15 wound healing/0h/si#1 -3.jpg]

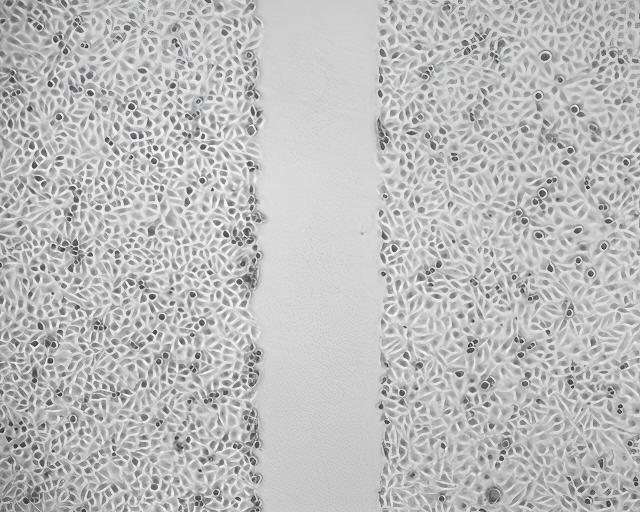

Supplement: Supplementary file 3 [file DataSheet3.zip › original data/Figure 6(Cell experiment)/HCT15 wound healing/0h/si#2 -1.jpg]

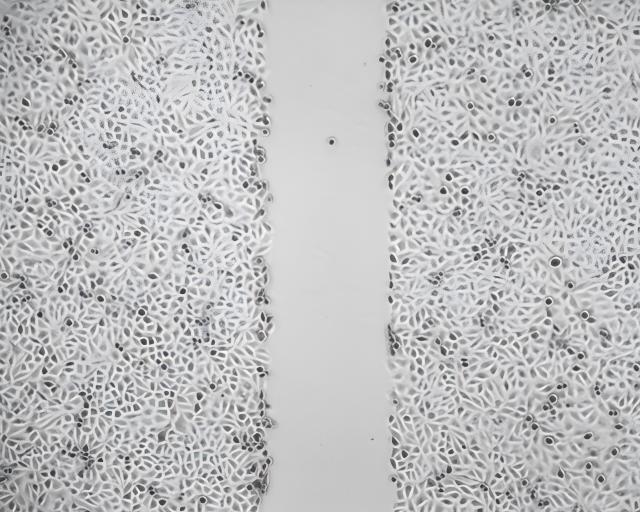

Supplement: Supplementary file 3 [file DataSheet3.zip › original data/Figure 6(Cell experiment)/HCT15 wound healing/0h/si#2 -2.jpg]

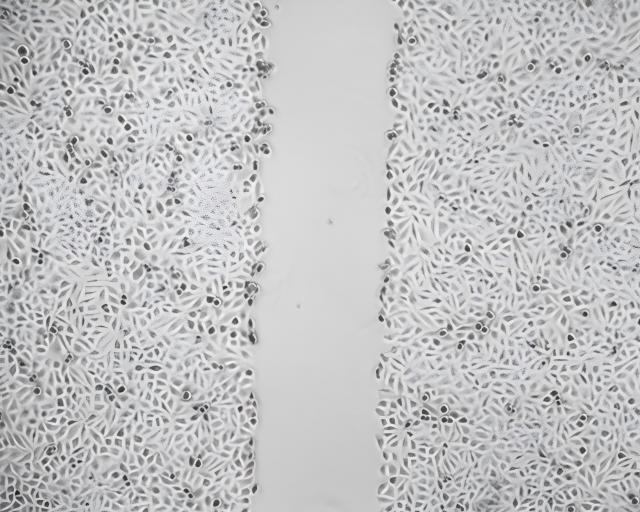

Supplement: Supplementary file 3 [file DataSheet3.zip › original data/Figure 6(Cell experiment)/HCT15 wound healing/0h/si#2 -3.jpg]

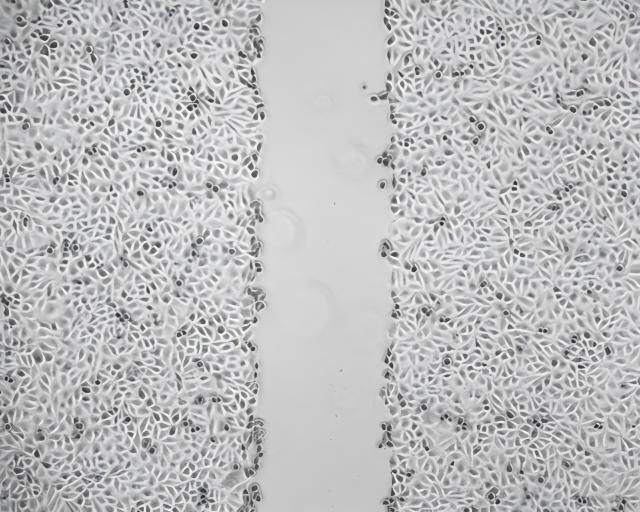

Supplement: Supplementary file 3 [file DataSheet3.zip › original data/Figure 6(Cell experiment)/HCT15 wound healing/0h/siCtrl -1.jpg]

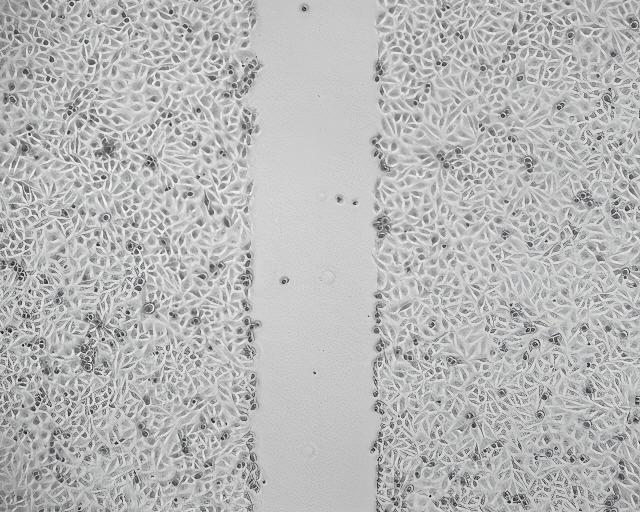

Supplement: Supplementary file 3 [file DataSheet3.zip › original data/Figure 6(Cell experiment)/HCT15 wound healing/0h/siCtrl -2.jpg]

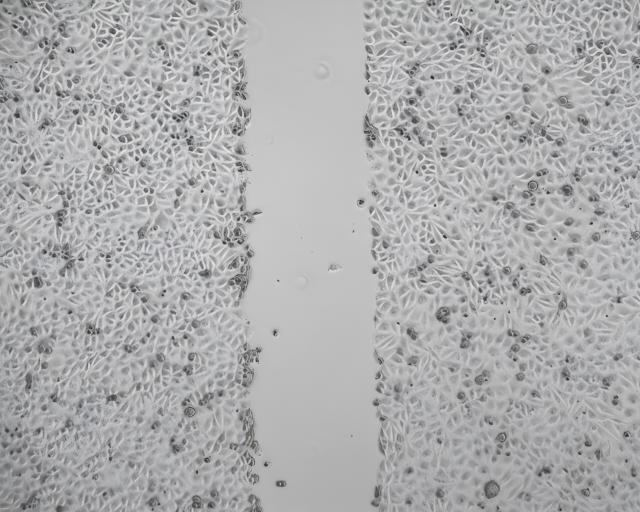

Supplement: Supplementary file 3 [file DataSheet3.zip › original data/Figure 6(Cell experiment)/HCT15 wound healing/0h/siCtrl -3.jpg]

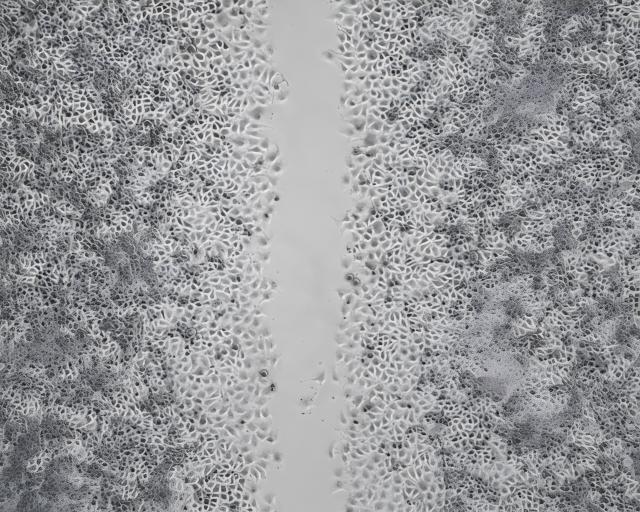

Supplement: Supplementary file 3 [file DataSheet3.zip › original data/Figure 6(Cell experiment)/HCT15 wound healing/24h/si#1 -1.jpg]

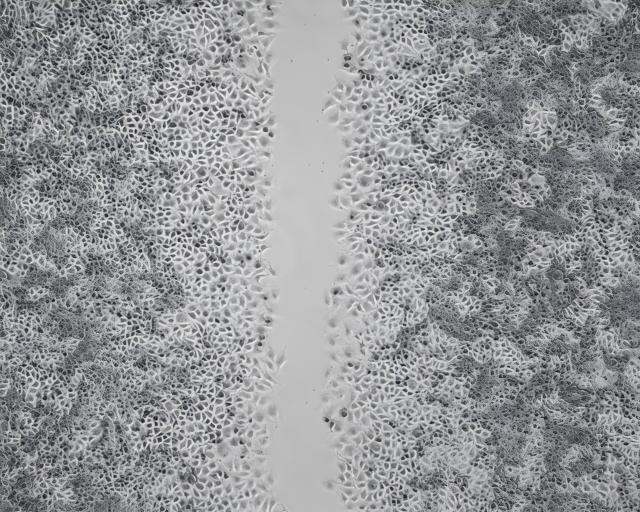

Supplement: Supplementary file 3 [file DataSheet3.zip › original data/Figure 6(Cell experiment)/HCT15 wound healing/24h/si#1 -2.jpg]

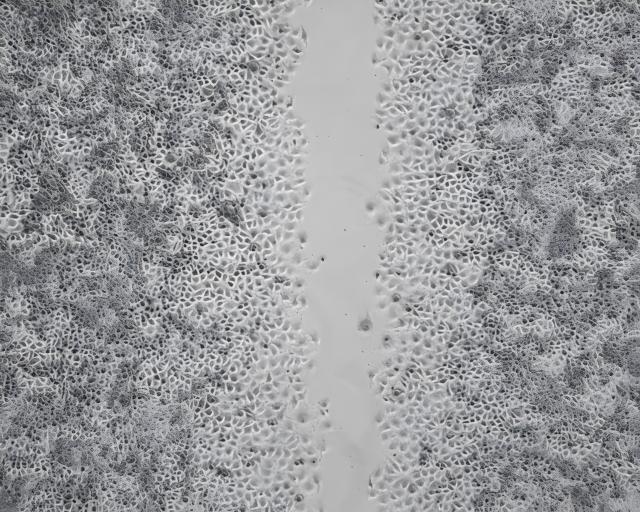

Supplement: Supplementary file 3 [file DataSheet3.zip › original data/Figure 6(Cell experiment)/HCT15 wound healing/24h/si#1 -3.jpg]

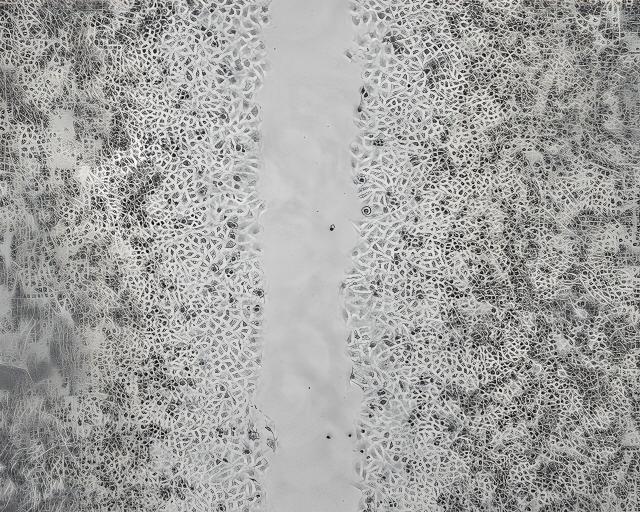

Supplement: Supplementary file 3 [file DataSheet3.zip › original data/Figure 6(Cell experiment)/HCT15 wound healing/24h/si#2 -1.jpg]

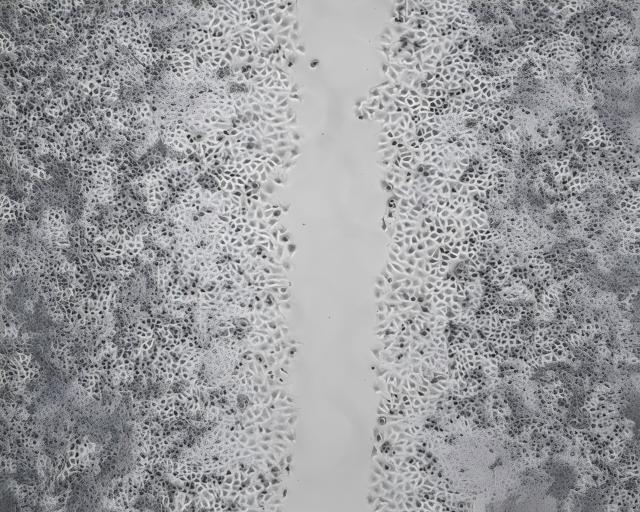

Supplement: Supplementary file 3 [file DataSheet3.zip › original data/Figure 6(Cell experiment)/HCT15 wound healing/24h/si#2 -2.jpg]

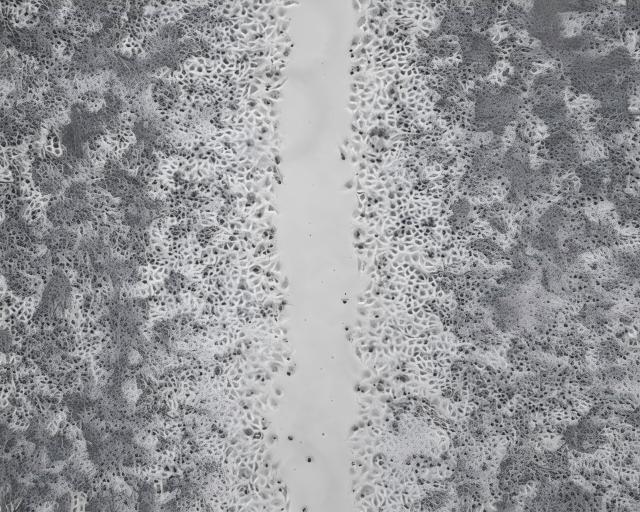

Supplement: Supplementary file 3 [file DataSheet3.zip › original data/Figure 6(Cell experiment)/HCT15 wound healing/24h/si#2 -3.jpg]

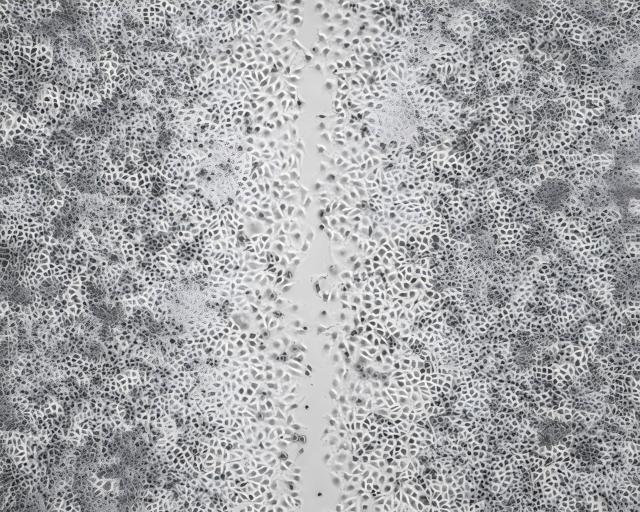

Supplement: Supplementary file 3 [file DataSheet3.zip › original data/Figure 6(Cell experiment)/HCT15 wound healing/24h/siCtrl -1.jpg]

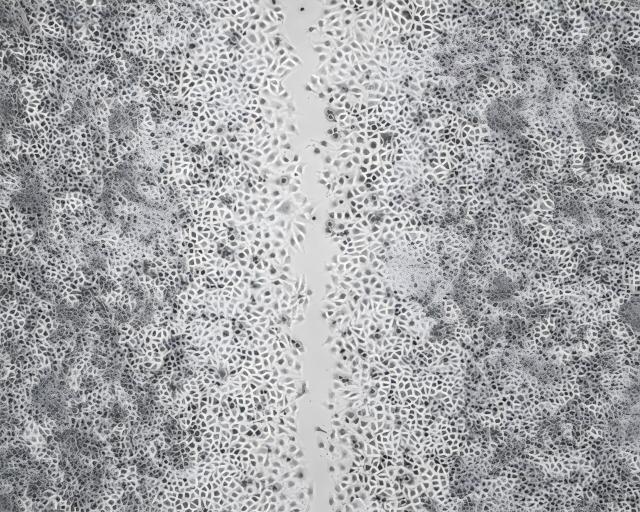

Supplement: Supplementary file 3 [file DataSheet3.zip › original data/Figure 6(Cell experiment)/HCT15 wound healing/24h/siCtrl -2.jpg]

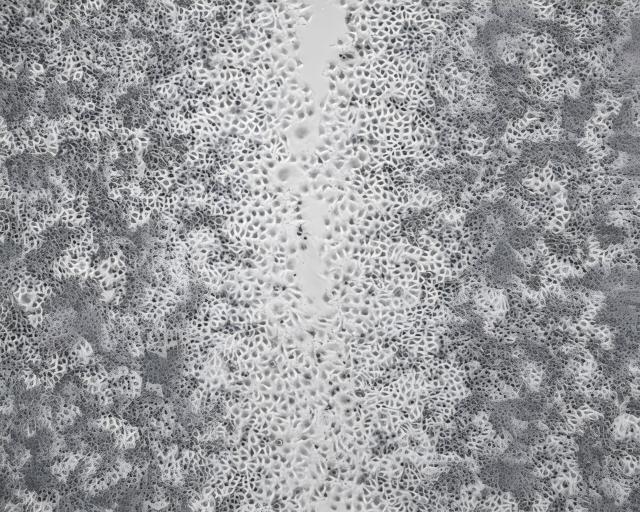

Supplement: Supplementary file 3 [file DataSheet3.zip › original data/Figure 6(Cell experiment)/HCT15 wound healing/24h/siCtrl -3.jpg]

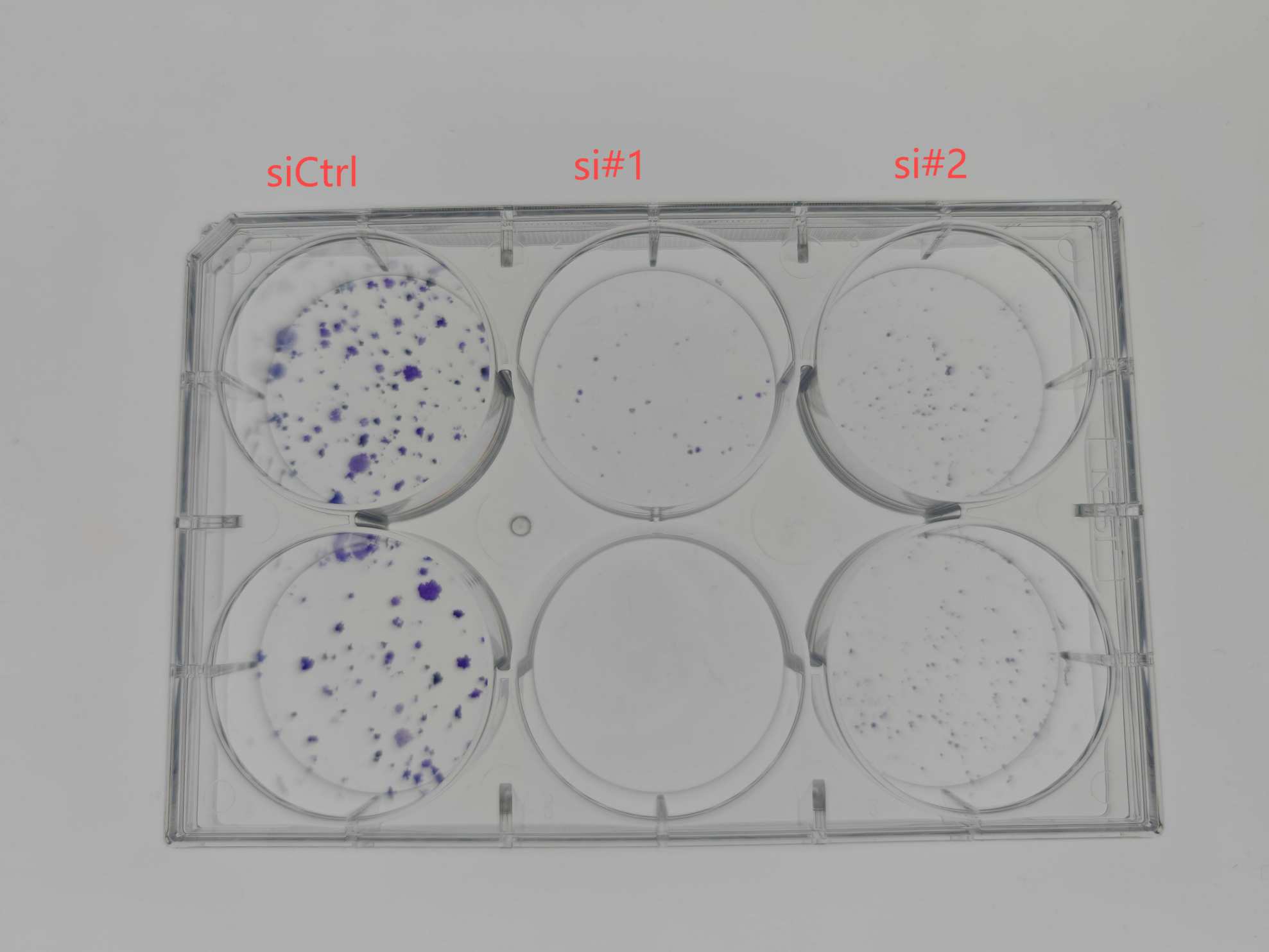

Supplement: Supplementary file 3 [file DataSheet3.zip › original data/Figure 6(Cell experiment)/RKO colony formation/1.jpg]

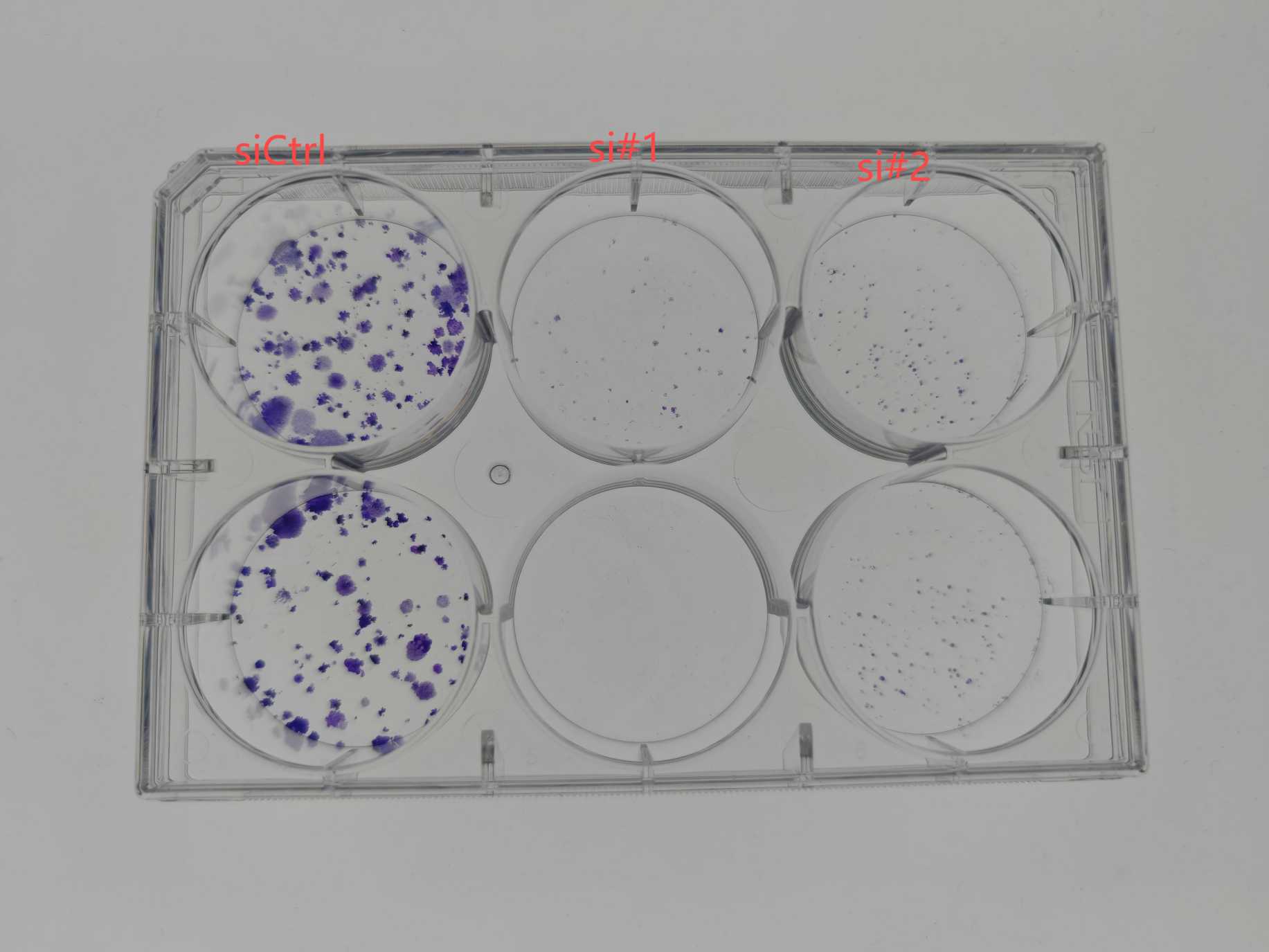

Supplement: Supplementary file 3 [file DataSheet3.zip › original data/Figure 6(Cell experiment)/RKO colony formation/2.jpg]

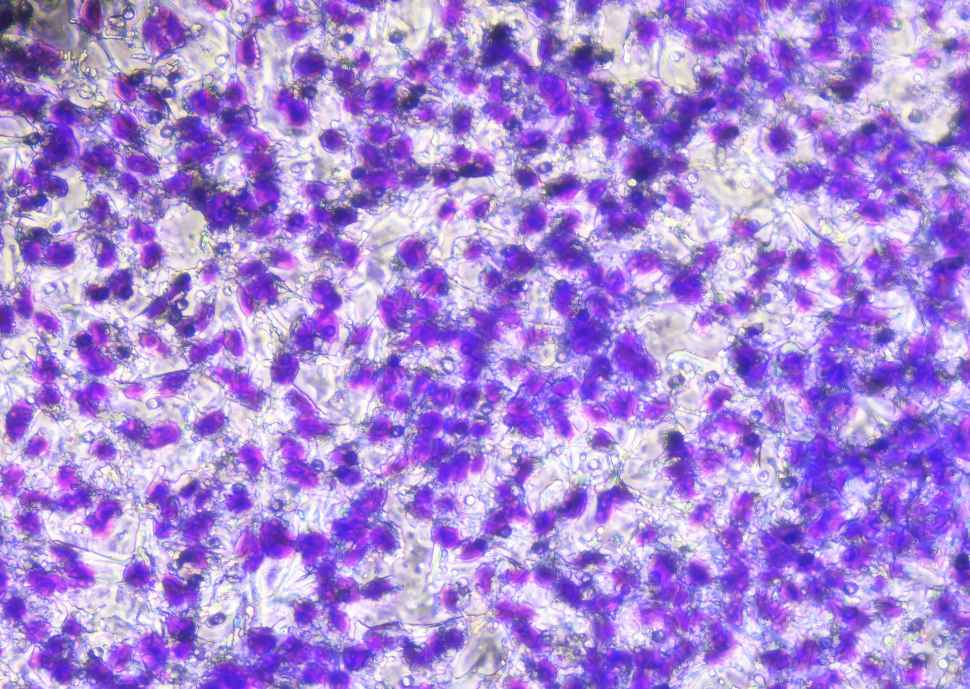

Supplement: Supplementary file 3 [file DataSheet3.zip › original data/Figure 6(Cell experiment)/RKO transwell/invasion/si#1 -1.jpg]

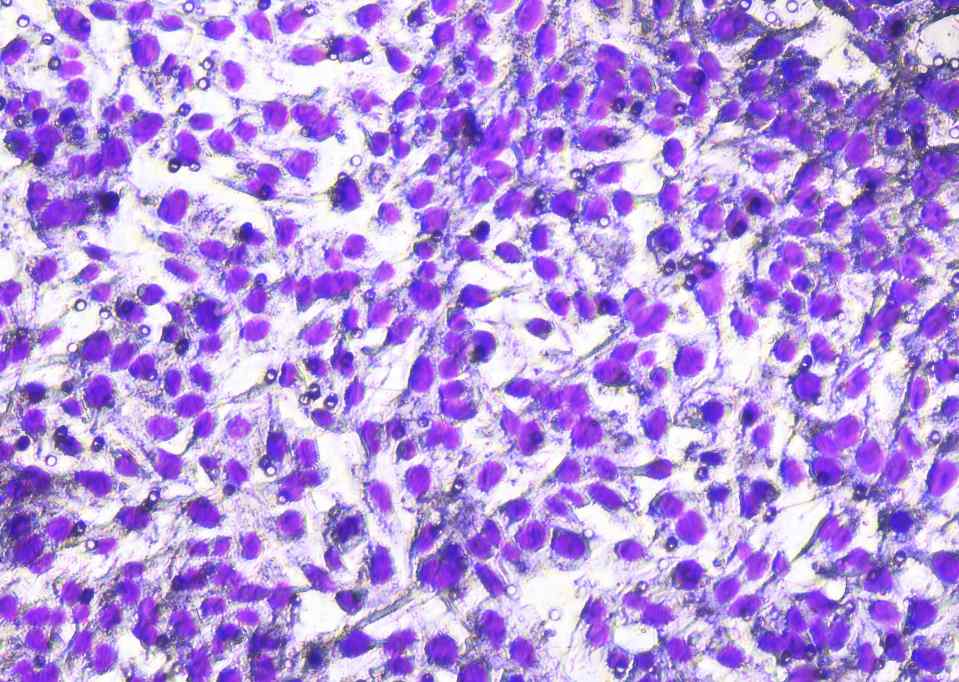

Supplement: Supplementary file 3 [file DataSheet3.zip › original data/Figure 6(Cell experiment)/RKO transwell/invasion/si#1 -2.jpg]

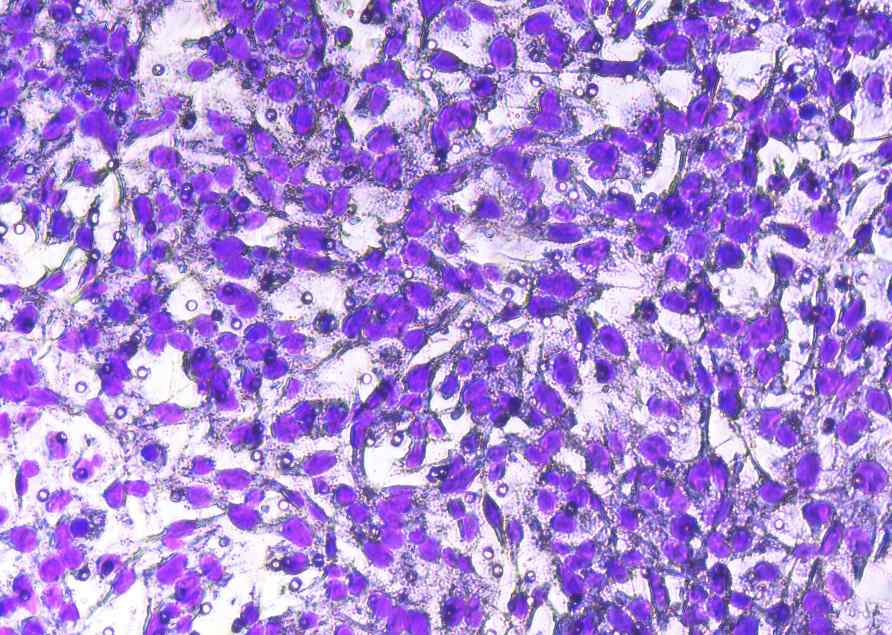

Supplement: Supplementary file 3 [file DataSheet3.zip › original data/Figure 6(Cell experiment)/RKO transwell/invasion/si#1 -3.jpg]

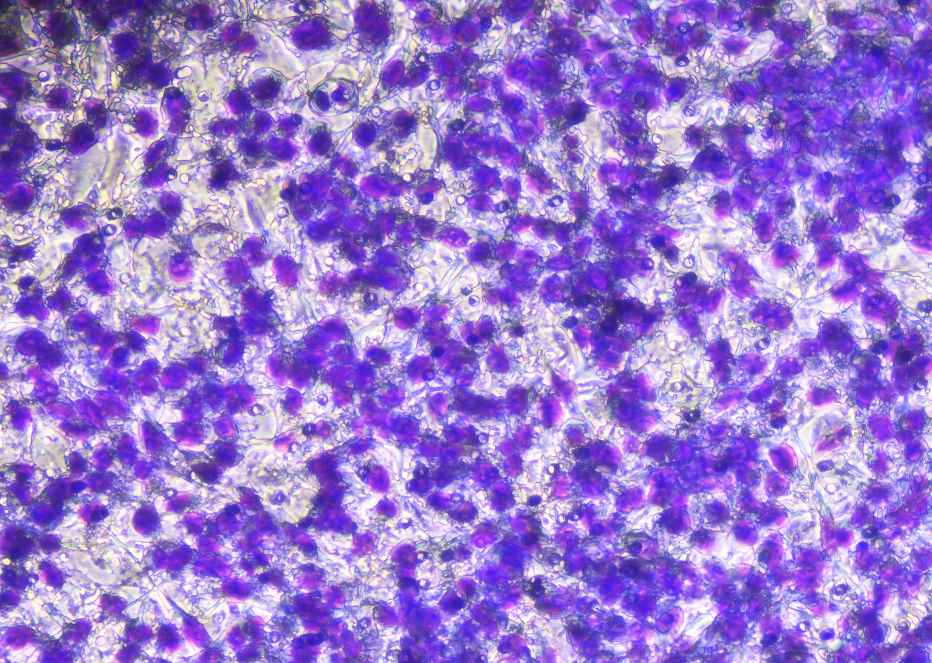

Supplement: Supplementary file 3 [file DataSheet3.zip › original data/Figure 6(Cell experiment)/RKO transwell/invasion/si#2 -1.jpg]

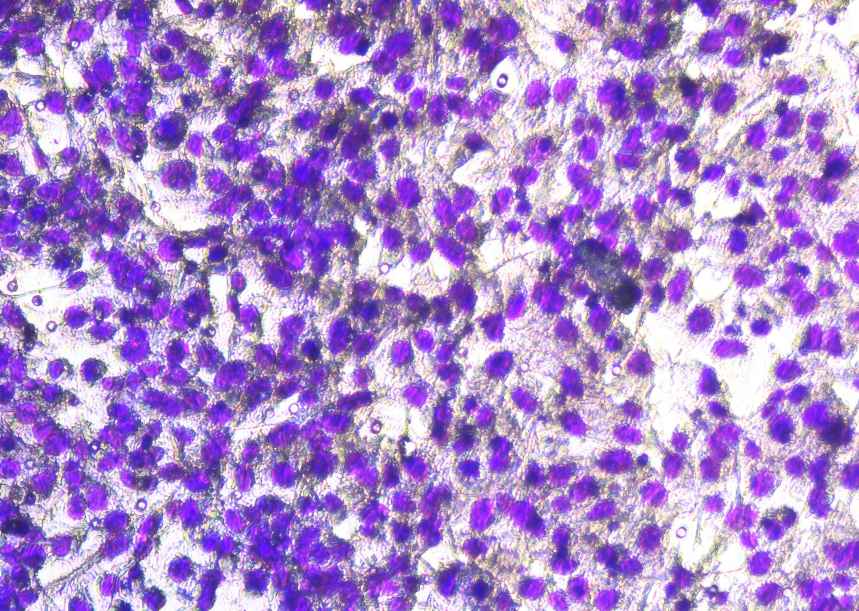

Supplement: Supplementary file 3 [file DataSheet3.zip › original data/Figure 6(Cell experiment)/RKO transwell/invasion/si#2 -2.jpg]

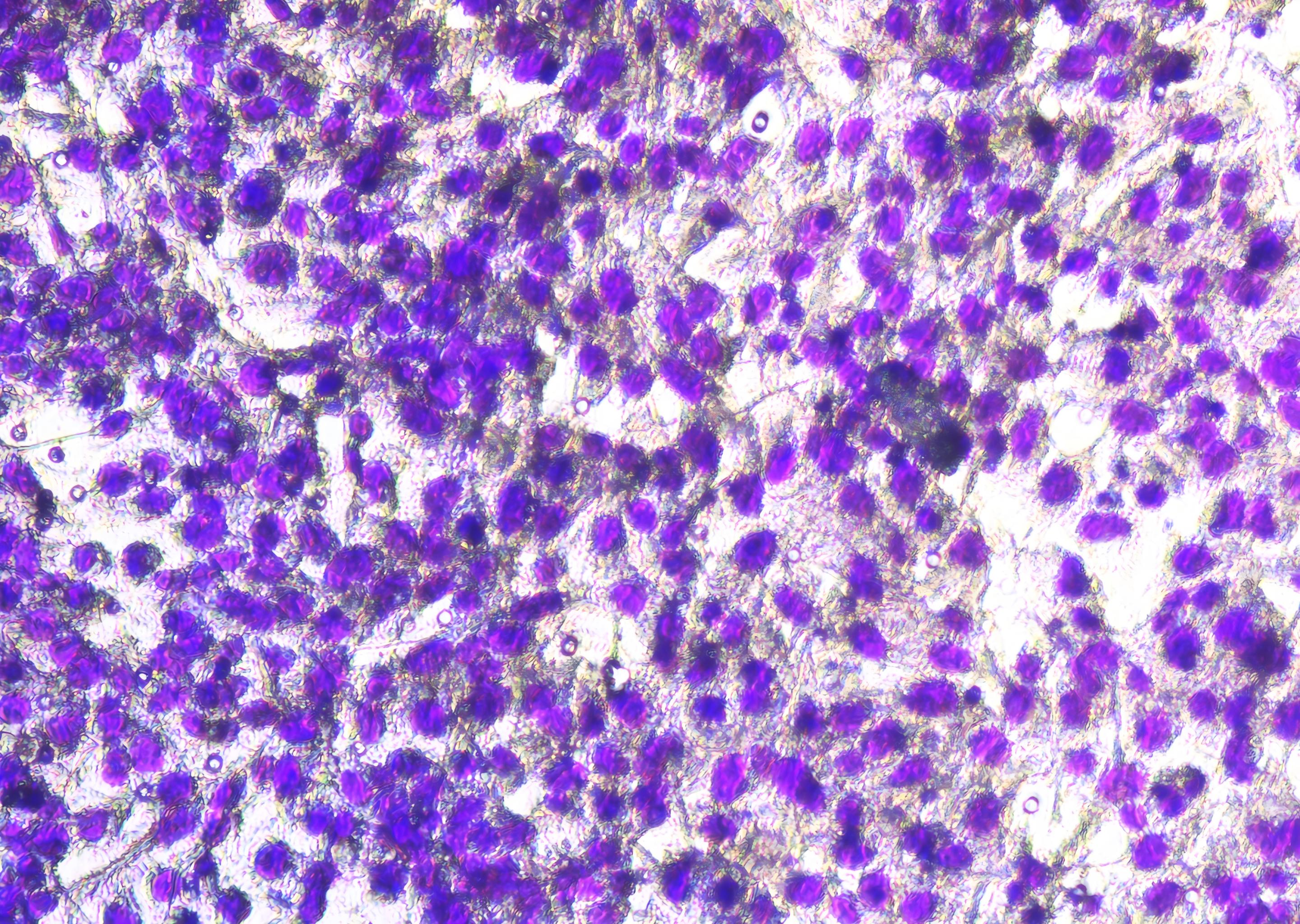

Supplement: Supplementary file 3 [file DataSheet3.zip › original data/Figure 6(Cell experiment)/RKO transwell/invasion/si#2 -3.jpg]

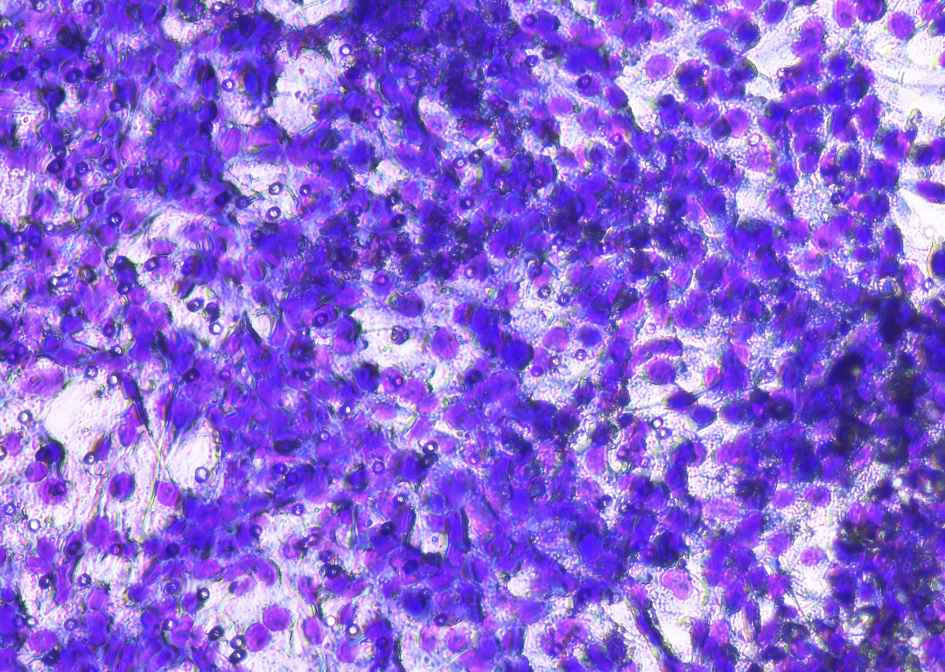

Supplement: Supplementary file 3 [file DataSheet3.zip › original data/Figure 6(Cell experiment)/RKO transwell/invasion/siCtrl -1.jpg]

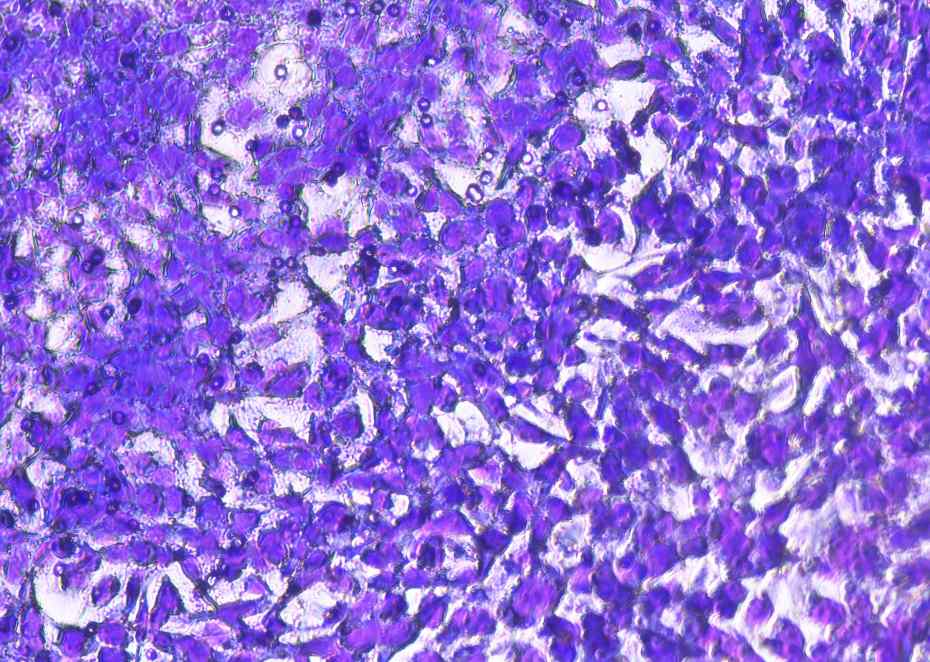

Supplement: Supplementary file 3 [file DataSheet3.zip › original data/Figure 6(Cell experiment)/RKO transwell/invasion/siCtrl -2.jpg]

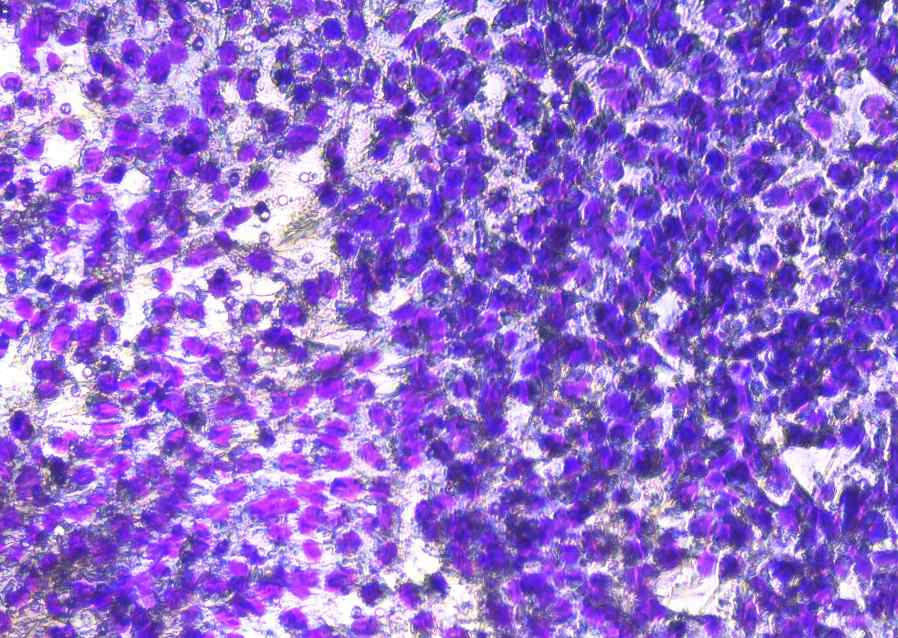

Supplement: Supplementary file 3 [file DataSheet3.zip › original data/Figure 6(Cell experiment)/RKO transwell/invasion/siCtrl -3.jpg]

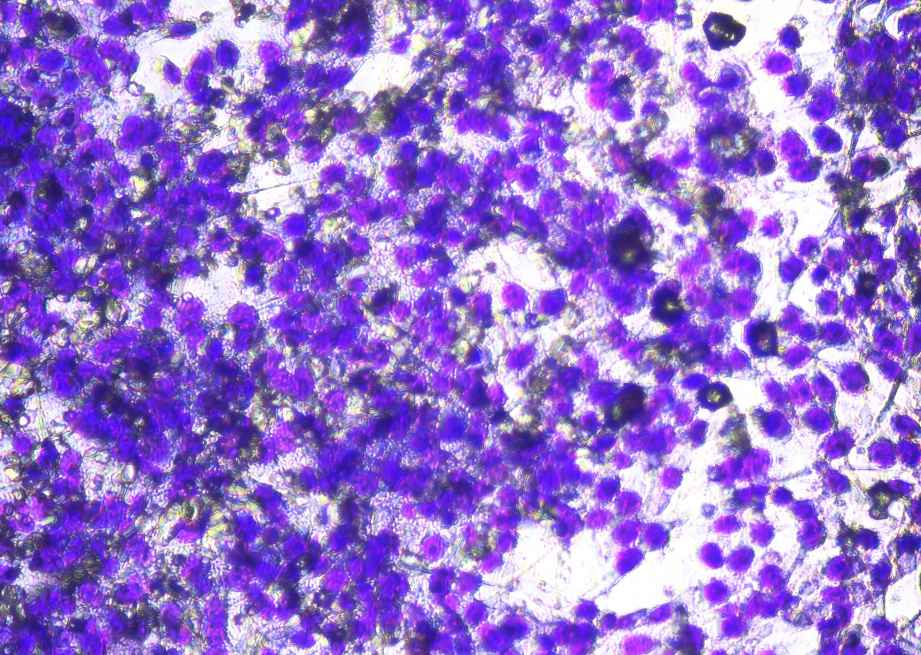

Supplement: Supplementary file 3 [file DataSheet3.zip › original data/Figure 6(Cell experiment)/RKO transwell/Migration/si#1 -1.jpg]

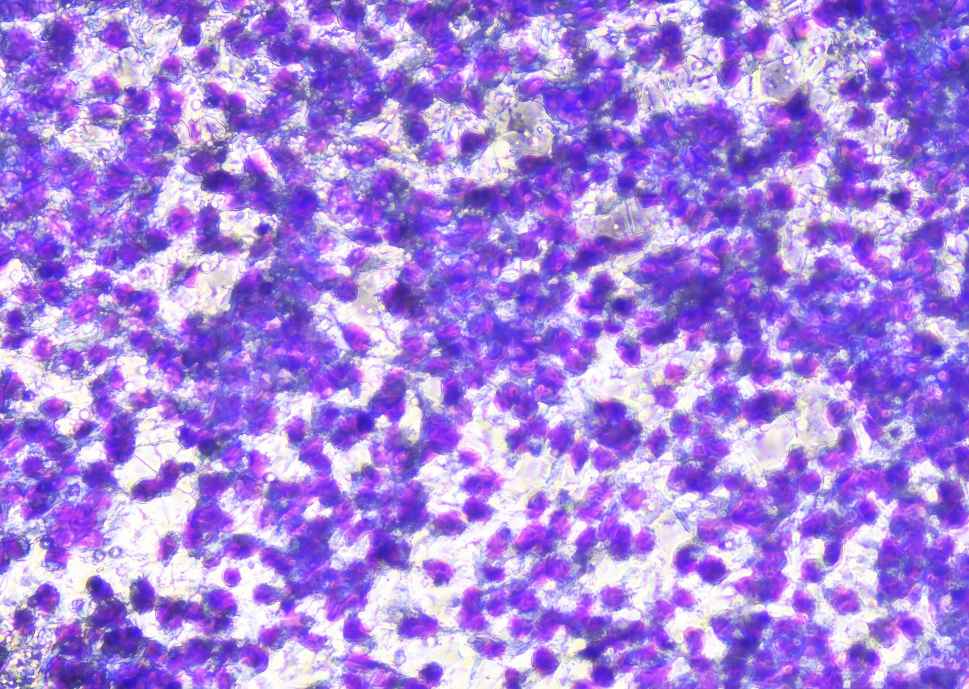

Supplement: Supplementary file 3 [file DataSheet3.zip › original data/Figure 6(Cell experiment)/RKO transwell/Migration/si#1 -2.jpg]

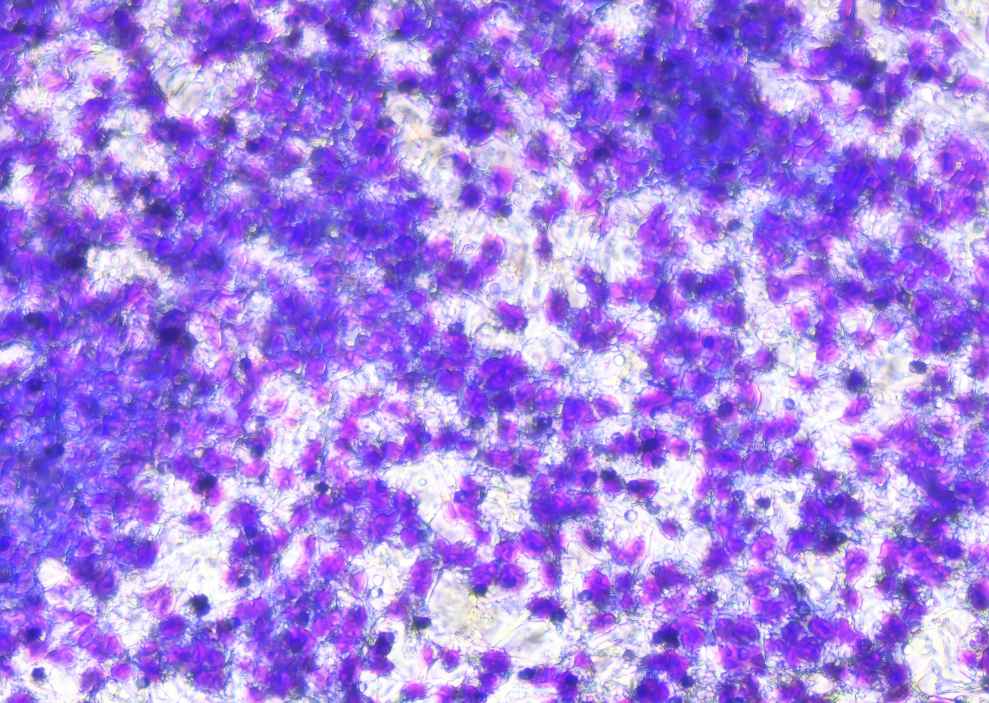

Supplement: Supplementary file 3 [file DataSheet3.zip › original data/Figure 6(Cell experiment)/RKO transwell/Migration/si#1 -3.jpg]

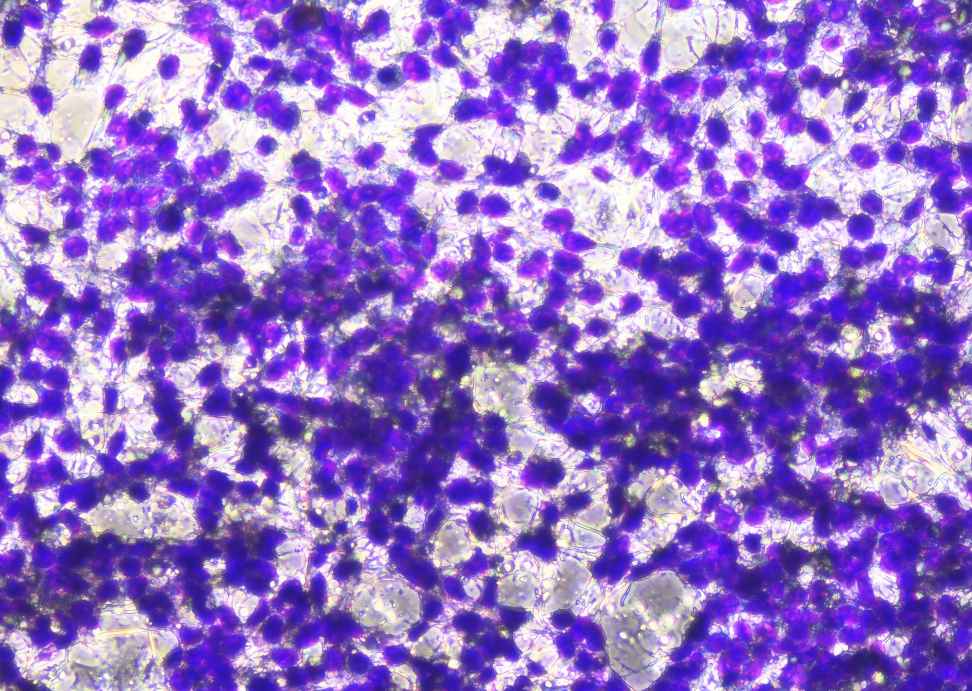

Supplement: Supplementary file 3 [file DataSheet3.zip › original data/Figure 6(Cell experiment)/RKO transwell/Migration/si#2 -1.jpg]

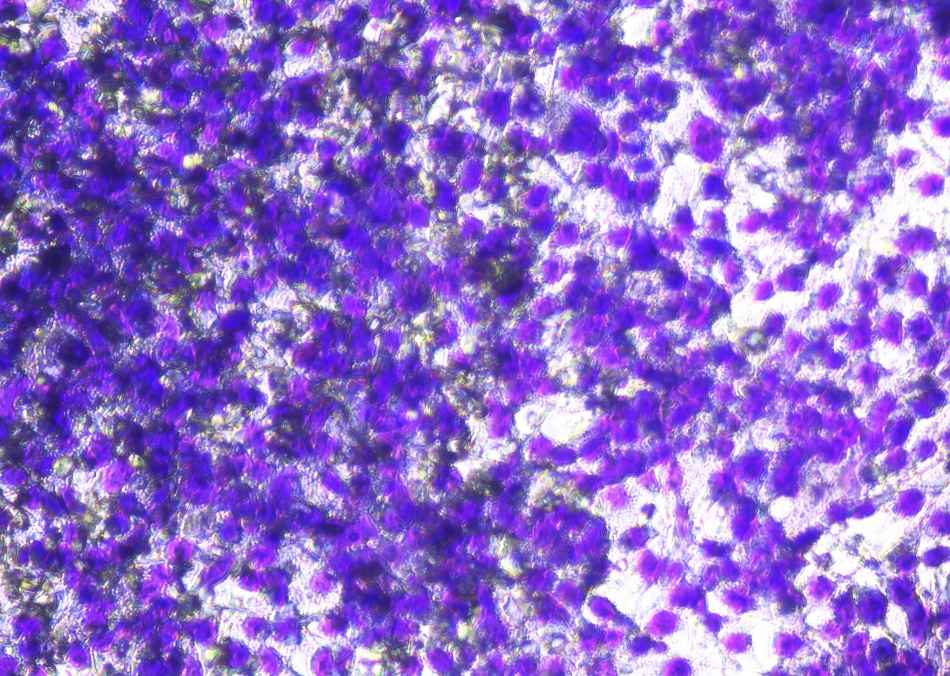

Supplement: Supplementary file 3 [file DataSheet3.zip › original data/Figure 6(Cell experiment)/RKO transwell/Migration/si#2 -2.jpg]

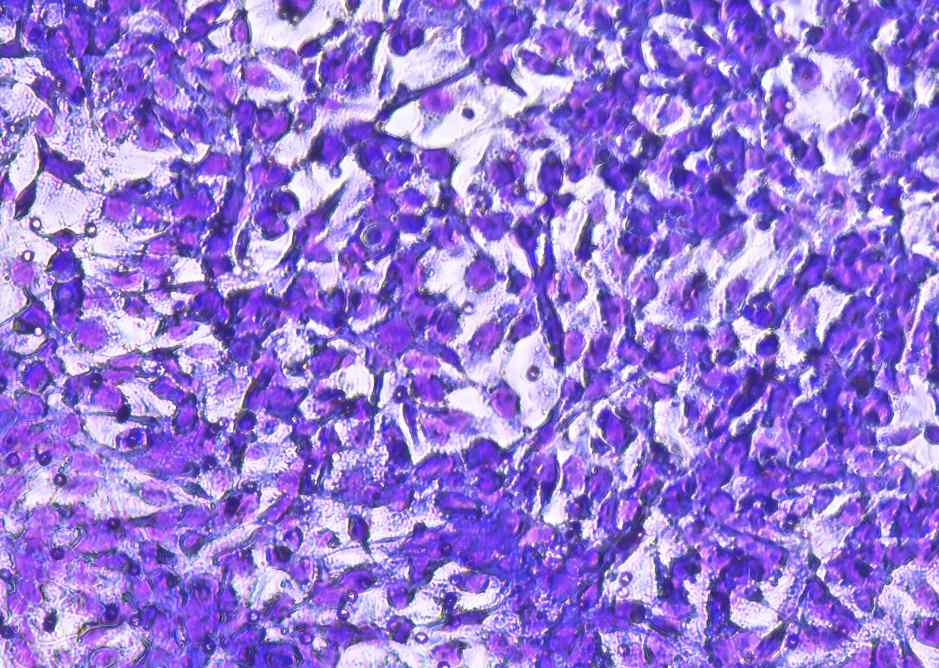

Supplement: Supplementary file 3 [file DataSheet3.zip › original data/Figure 6(Cell experiment)/RKO transwell/Migration/si#2 -3.jpg]

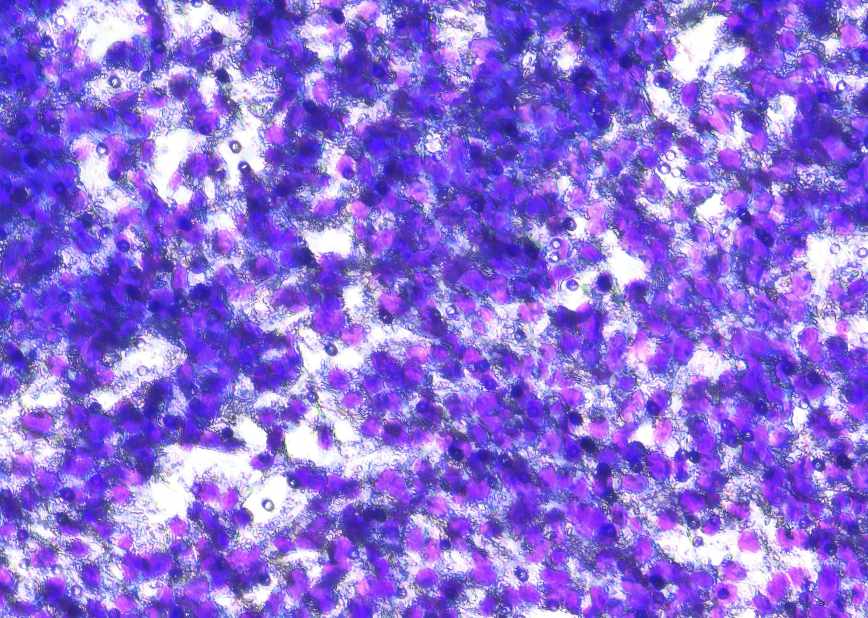

Supplement: Supplementary file 3 [file DataSheet3.zip › original data/Figure 6(Cell experiment)/RKO transwell/Migration/siCtrl -1.jpg]

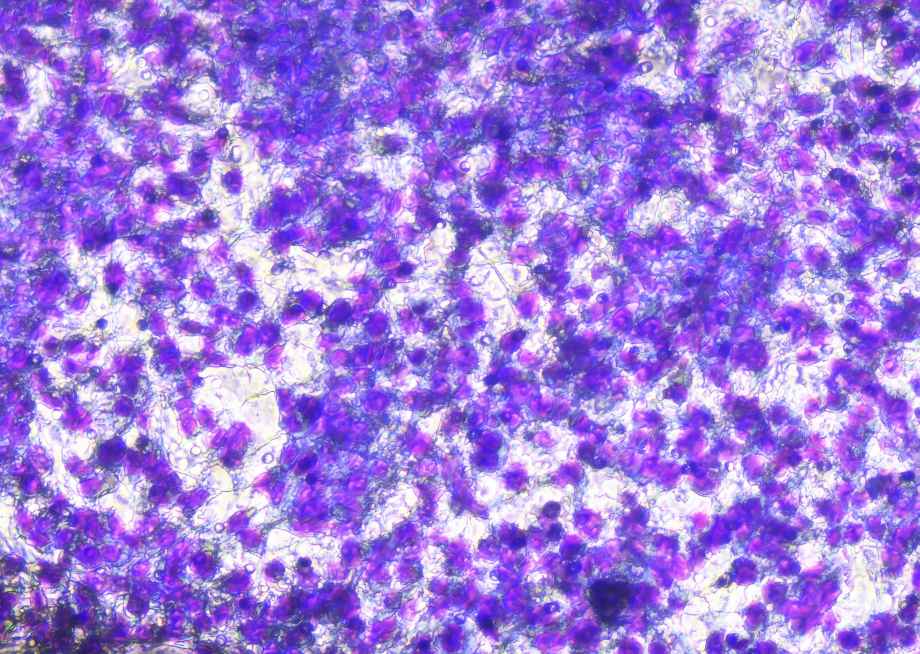

Supplement: Supplementary file 3 [file DataSheet3.zip › original data/Figure 6(Cell experiment)/RKO transwell/Migration/siCtrl -2.jpg]

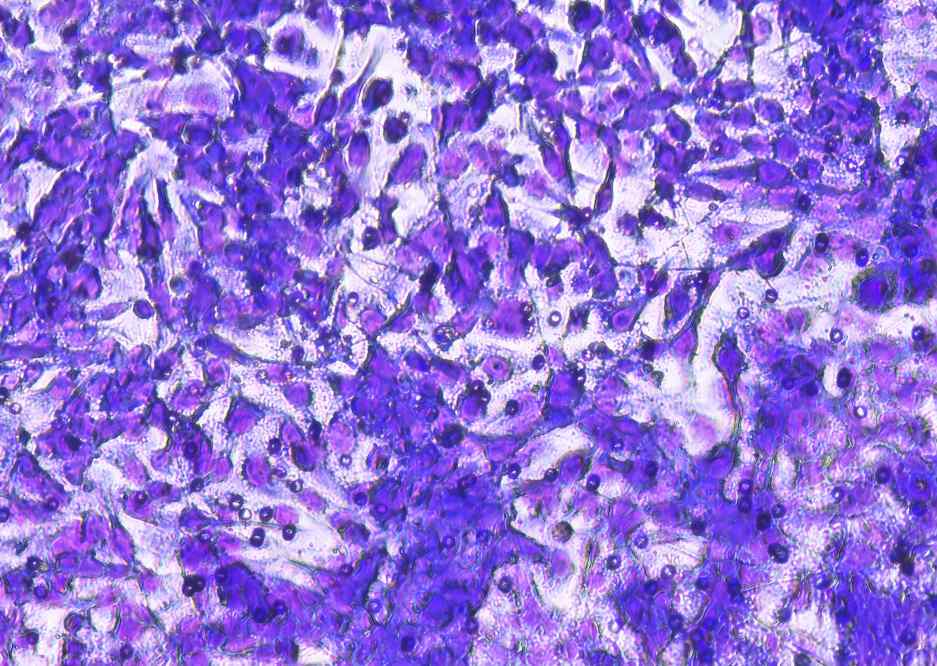

Supplement: Supplementary file 3 [file DataSheet3.zip › original data/Figure 6(Cell experiment)/RKO transwell/Migration/siCtrl -3.jpg]

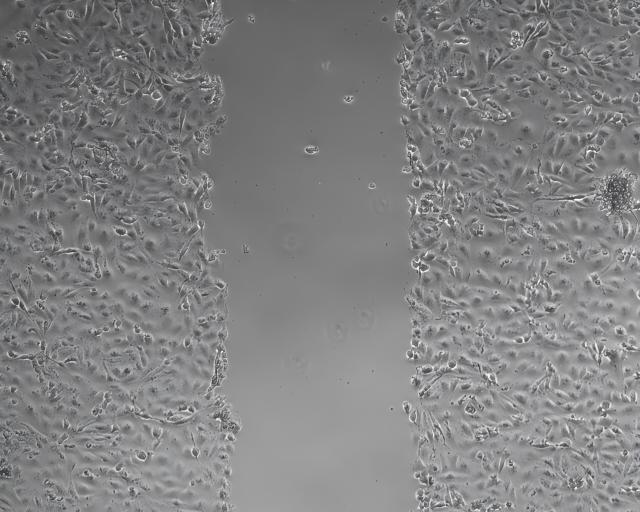

Supplement: Supplementary file 3 [file DataSheet3.zip › original data/Figure 6(Cell experiment)/RKO wound healing/0h/si#1 -1.jpg]

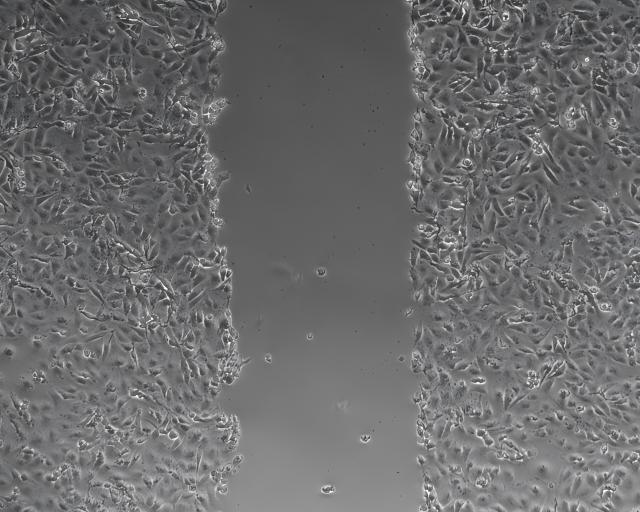

Supplement: Supplementary file 3 [file DataSheet3.zip › original data/Figure 6(Cell experiment)/RKO wound healing/0h/si#1 -2.jpg]

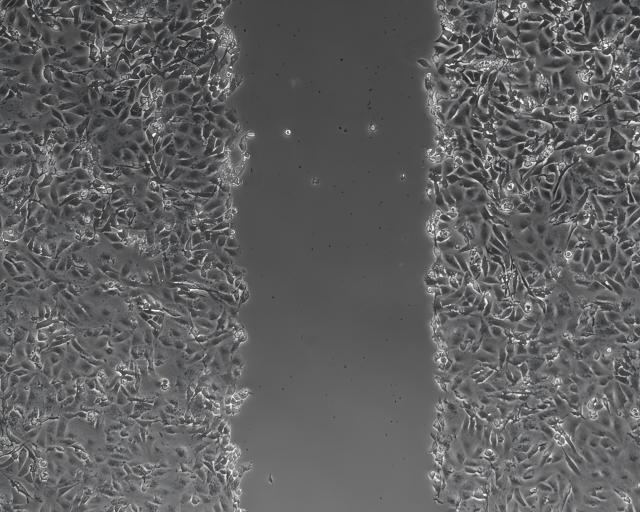

Supplement: Supplementary file 3 [file DataSheet3.zip › original data/Figure 6(Cell experiment)/RKO wound healing/0h/si#1 -3.jpg]

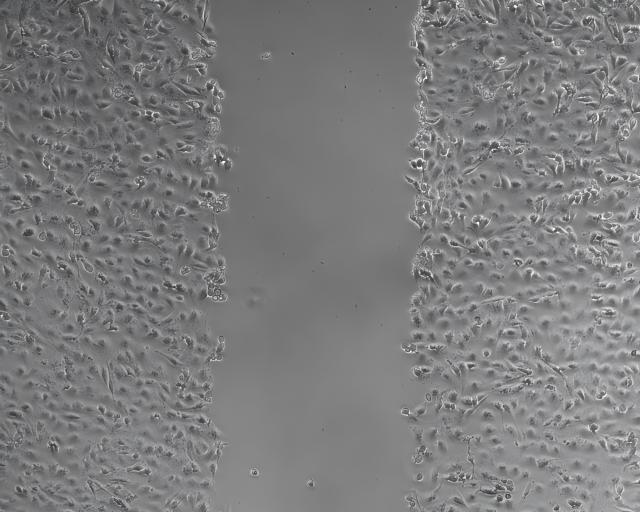

Supplement: Supplementary file 3 [file DataSheet3.zip › original data/Figure 6(Cell experiment)/RKO wound healing/0h/si#2 -1.jpg]

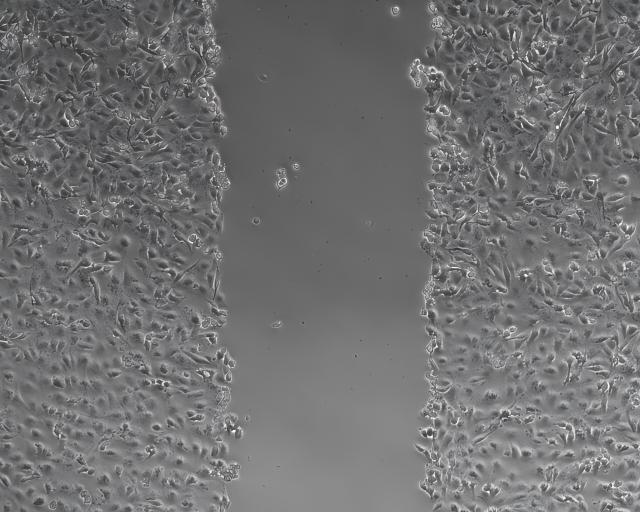

Supplement: Supplementary file 3 [file DataSheet3.zip › original data/Figure 6(Cell experiment)/RKO wound healing/0h/si#2 -2.jpg]

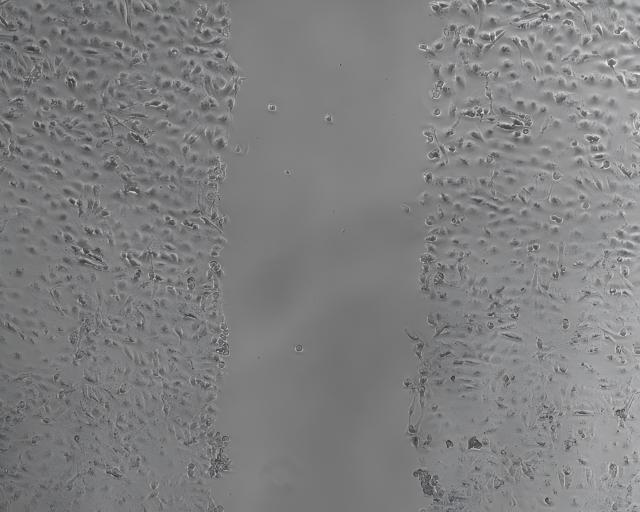

Supplement: Supplementary file 3 [file DataSheet3.zip › original data/Figure 6(Cell experiment)/RKO wound healing/0h/si#2 -3.jpg]

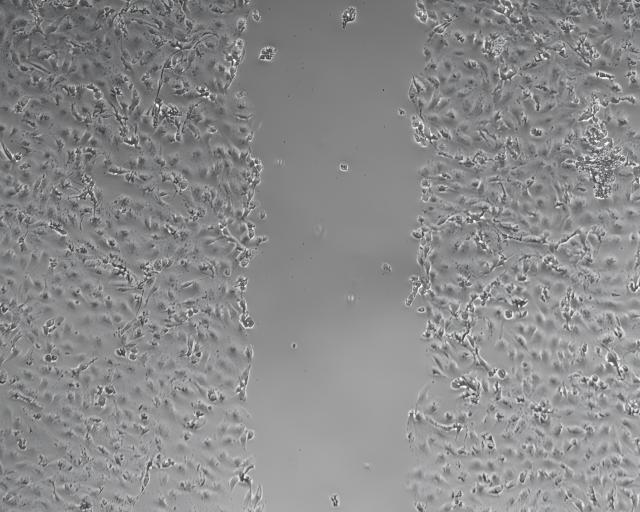

Supplement: Supplementary file 3 [file DataSheet3.zip › original data/Figure 6(Cell experiment)/RKO wound healing/0h/siCtrl -1.jpg]

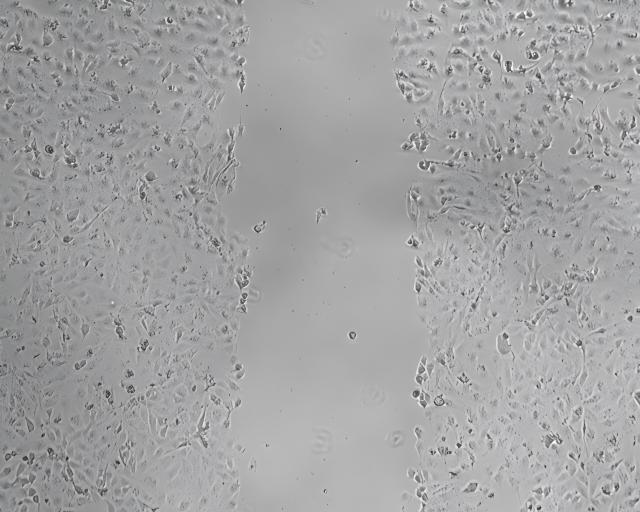

Supplement: Supplementary file 3 [file DataSheet3.zip › original data/Figure 6(Cell experiment)/RKO wound healing/0h/siCtrl -2.jpg]

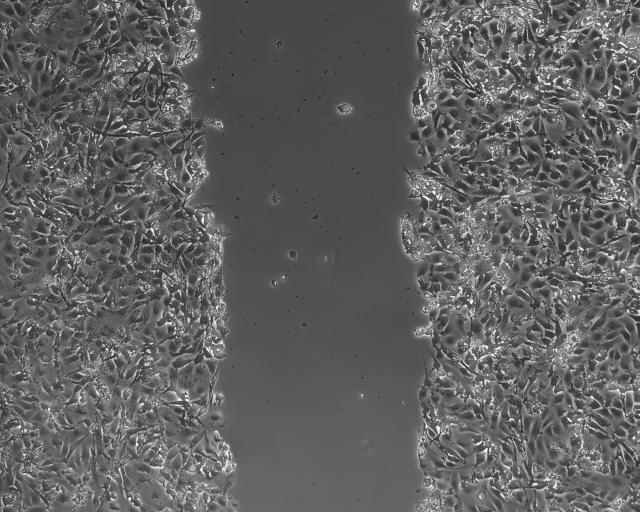

Supplement: Supplementary file 3 [file DataSheet3.zip › original data/Figure 6(Cell experiment)/RKO wound healing/0h/siCtrl -3.jpg]

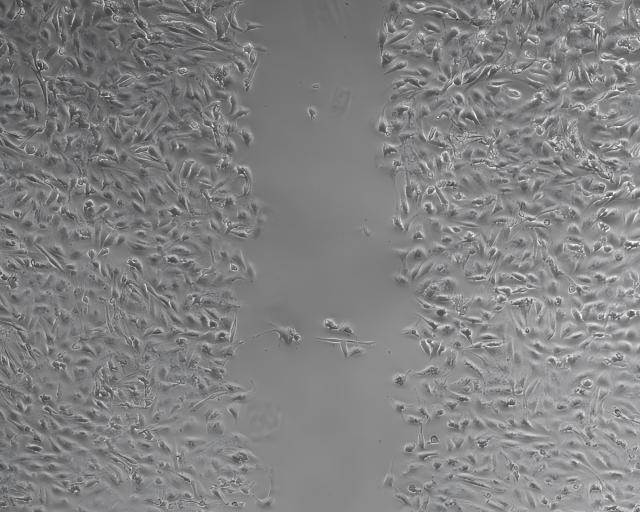

Supplement: Supplementary file 3 [file DataSheet3.zip › original data/Figure 6(Cell experiment)/RKO wound healing/24h/si#1 -1.jpg]

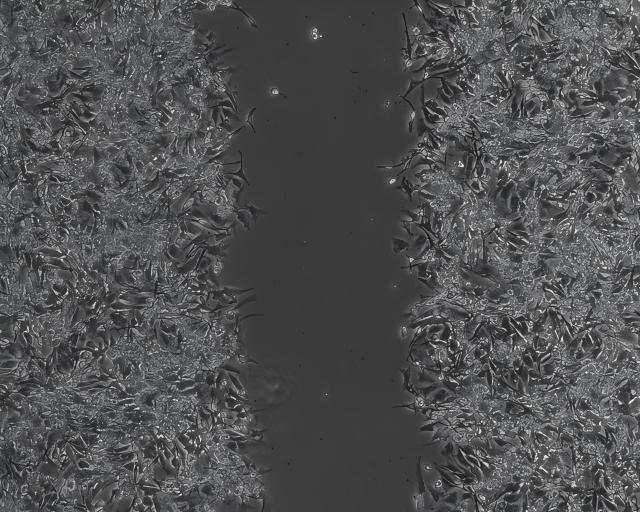

Supplement: Supplementary file 3 [file DataSheet3.zip › original data/Figure 6(Cell experiment)/RKO wound healing/24h/si#1 -2.jpg]

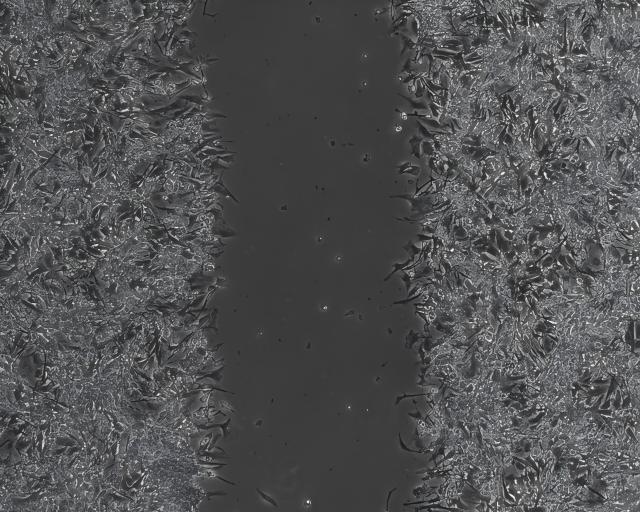

Supplement: Supplementary file 3 [file DataSheet3.zip › original data/Figure 6(Cell experiment)/RKO wound healing/24h/si#1 -3.jpg]

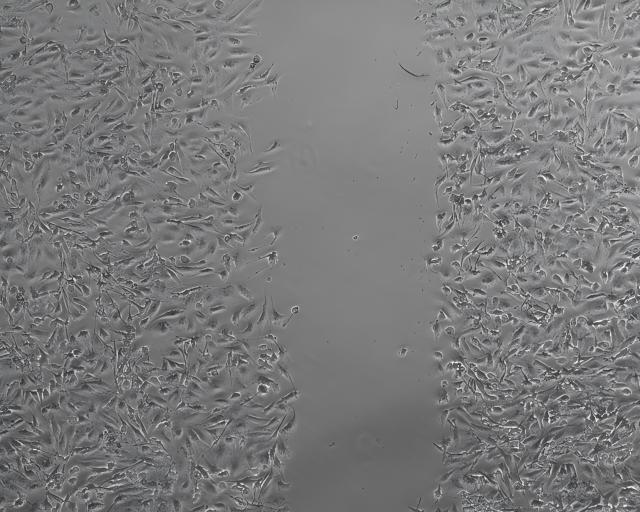

Supplement: Supplementary file 3 [file DataSheet3.zip › original data/Figure 6(Cell experiment)/RKO wound healing/24h/si#2 -1.jpg]

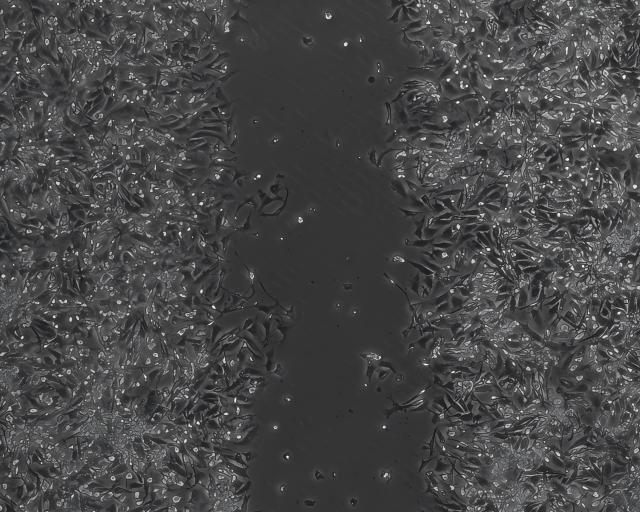

Supplement: Supplementary file 3 [file DataSheet3.zip › original data/Figure 6(Cell experiment)/RKO wound healing/24h/si#2 -2.jpg]

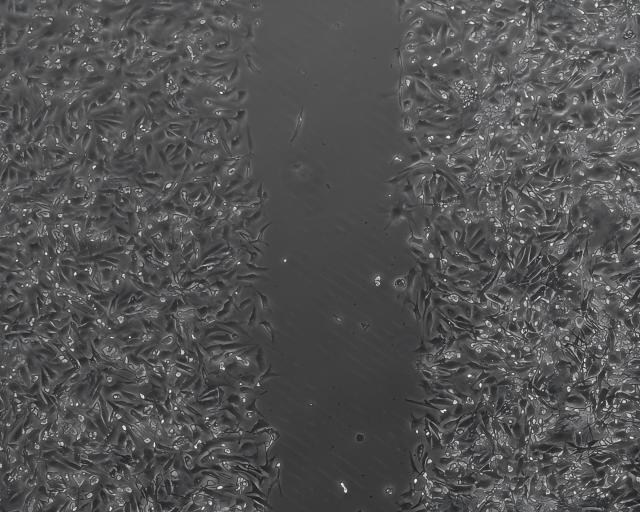

Supplement: Supplementary file 3 [file DataSheet3.zip › original data/Figure 6(Cell experiment)/RKO wound healing/24h/si#2 -3.jpg]

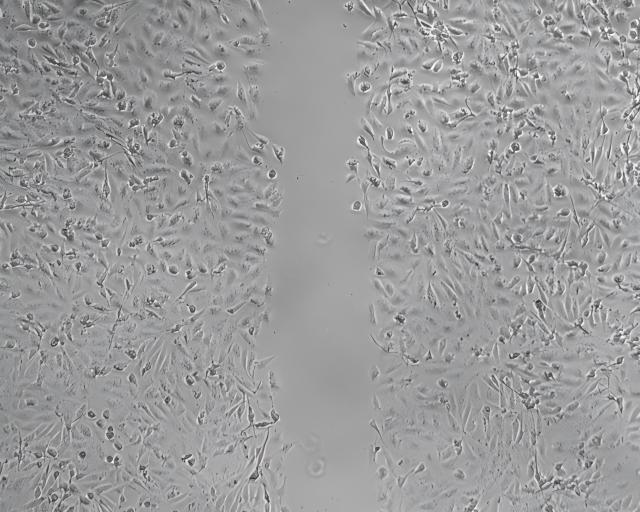

Supplement: Supplementary file 3 [file DataSheet3.zip › original data/Figure 6(Cell experiment)/RKO wound healing/24h/siCtrl -1.jpg]

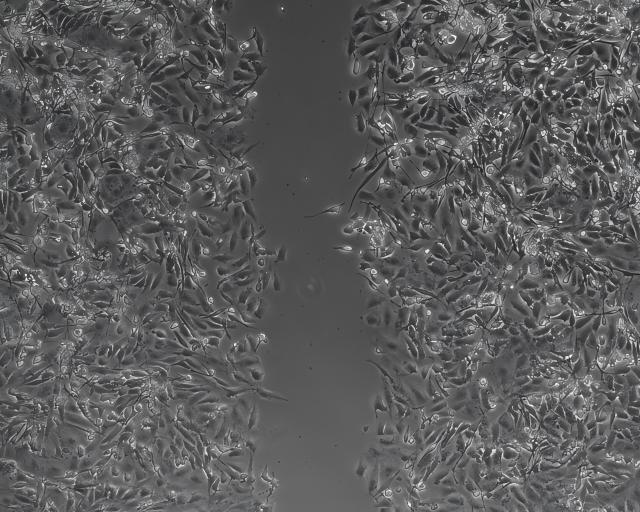

Supplement: Supplementary file 3 [file DataSheet3.zip › original data/Figure 6(Cell experiment)/RKO wound healing/24h/siCtrl -2.jpg]

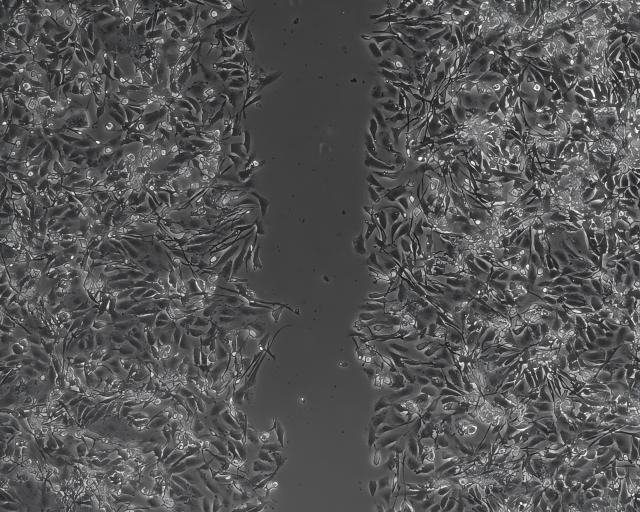

Supplement: Supplementary file 3 [file DataSheet3.zip › original data/Figure 6(Cell experiment)/RKO wound healing/24h/siCtrl -3.jpg]

β-actin and PRSS22—24 pairs of clinical samples















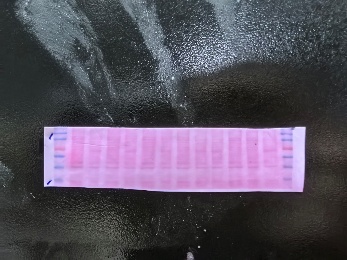

















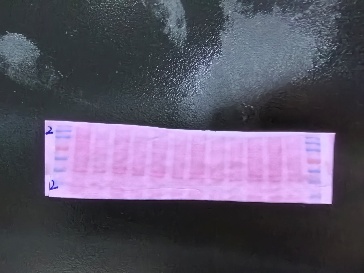






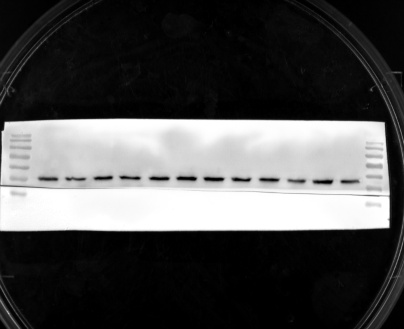









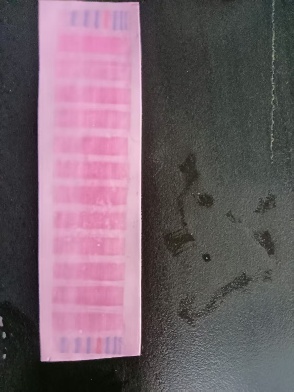
















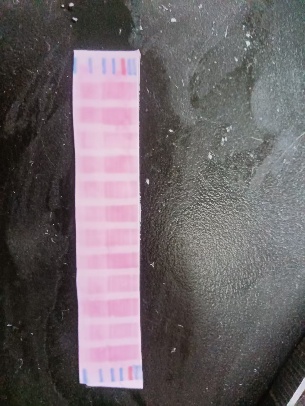


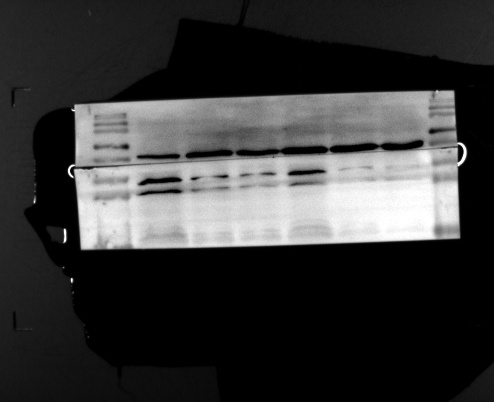
Cell line




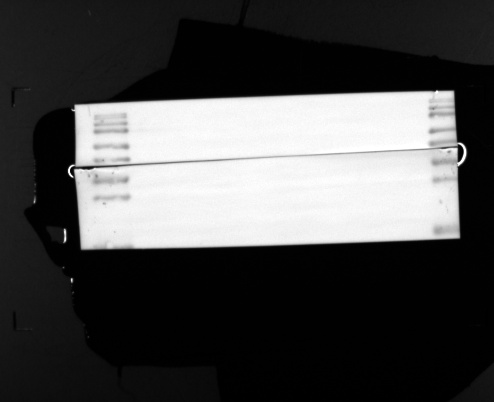

Supplement: Supplementary file 3 [file DataSheet3.zip › original data/Western blotting in Figure 5 and 6/all Western blotting original figure.docx]
