# Supplementary material for: Single-cell transcriptomics in colorectal cancer uncover the potential of metastasis and immune dysregulation of a cell cluster overexpressed PRSS22
Source: Front Immunol. 2025 May 20;16:1586428. doi: 10.3389/fimmu.2025.1586428 (PMC12130013; doi:10.3389/fimmu.2025.1586428)
Supplement: Supplementary file 4 [file Table1.docx]

Supplementary table 1: Boruta algorithm result

|  | meanImp | medianImp | minImp | maxImp | normHits | decision |
| --- | --- | --- | --- | --- | --- | --- |
| ANXA1 | 2.879407 | 2.894427 | -0.81477 | 4.957827 | 0.484848 | Tentative |
| AREG | 2.863796 | 2.856216 | 1.435147 | 4.449995 | 0.454545 | Tentative |
| ASS1 | 3.088606 | 3.124287 | 0.242041 | 5.733044 | 0.545455 | Tentative |
| BIRC3 | 2.715335 | 2.677745 | 0.551826 | 4.680249 | 0.383838 | Tentative |
| BST2 | 3.065728 | 3.048373 | 1.170099 | 4.299386 | 0.515152 | Tentative |
| CCL20 | 2.354068 | 2.318113 | 1.115288 | 3.277407 | 0.040404 | Rejected |
| CD55 | 2.87227 | 2.92699 | 1.635398 | 4.276689 | 0.454545 | Tentative |
| CD59 | 2.810363 | 2.682159 | 0.995356 | 5.882426 | 0.414141 | Tentative |
| CD74 | 4.334315 | 4.330185 | 2.832655 | 6.329225 | 0.828283 | Confirmed |
| CSTB | 3.137913 | 3.137593 | 1.603465 | 5.182285 | 0.525253 | Tentative |
| CXCL1 | 3.38674 | 3.44494 | 1.499619 | 5.177602 | 0.606061 | Tentative |
| CXCL14 | 4.3634 | 4.406707 | 1.970562 | 6.276528 | 0.757576 | Confirmed |
| CXCL2 | 2.697173 | 2.618639 | 1.019244 | 4.055944 | 0.232323 | Rejected |
| CXCL3 | 3.145156 | 3.148903 | 1.387453 | 5.258783 | 0.474747 | Tentative |
| CXCL8 | 1.42033 | 1.269243 | 0.499191 | 3.680126 | 0.020202 | Rejected |
| EDN1 | 2.251471 | 2.321506 | 0.579433 | 3.073768 | 0.030303 | Rejected |
| ERO1A | 2.000069 | 2.03895 | 0.570476 | 3.236402 | 0.030303 | Rejected |
| F3 | 2.748694 | 2.702092 | 1.089971 | 4.313449 | 0.424242 | Tentative |
| FGFBP1 | 2.659299 | 2.604691 | 0.283931 | 4.420464 | 0.373737 | Tentative |
| HLA-DRA | 5.116027 | 5.136086 | 3.311976 | 7.01637 | 0.858586 | Confirmed |
| HLA-DRB1 | 4.455538 | 4.458902 | 2.86096 | 6.105368 | 0.808081 | Confirmed |
| IFI6 | 2.310944 | 2.398898 | 0.620278 | 3.444444 | 0.050505 | Rejected |
| IL32 | 2.43871 | 2.397835 | 1.429622 | 3.590502 | 0.040404 | Rejected |
| INSIG1 | 1.275765 | 1.329731 | 0.098875 | 3.060375 | 0.010101 | Rejected |
| ISG15 | 3.549272 | 3.609116 | 1.964324 | 5.446369 | 0.636364 | Tentative |
| KLK10 | 3.921734 | 3.882818 | 2.11068 | 5.925552 | 0.69697 | Confirmed |
| KLK6 | 3.451873 | 3.52526 | 0.652431 | 4.955233 | 0.606061 | Tentative |
| KRT17 | 2.884818 | 2.91921 | 0.77166 | 3.880951 | 0.434343 | Tentative |
| KRT23 | 2.931733 | 3.014867 | 0.921559 | 4.757612 | 0.454545 | Tentative |
| KRT7 | 3.236304 | 3.230227 | 1.494754 | 5.19797 | 0.555556 | Tentative |
| LAMC2 | 3.228202 | 3.19605 | -0.19112 | 5.269014 | 0.565657 | Tentative |
| LCN2 | 4.007998 | 4.02788 | 2.284568 | 5.747175 | 0.717172 | Confirmed |
| MMP7 | 4.184534 | 4.15192 | 2.259379 | 6.320836 | 0.767677 | Confirmed |
| PDZK1IP1 | 3.386223 | 3.494473 | 1.598591 | 4.937068 | 0.565657 | Tentative |
| PI3 | 3.746248 | 3.862349 | 1.581309 | 5.092232 | 0.676768 | Tentative |
| PRSS22 | 2.556168 | 2.517123 | 0.568658 | 4.47889 | 0.383838 | Tentative |
| S100A2 | 2.832082 | 2.990825 | 0.920734 | 4.679632 | 0.262626 | Rejected |
| S100A4 | 2.992488 | 2.966809 | 0.53326 | 4.401239 | 0.515152 | Tentative |
| S100P | 4.041488 | 4.05698 | 2.012943 | 6.076426 | 0.767677 | Confirmed |
| SCD | 2.508757 | 2.577918 | 0.708818 | 4.236962 | 0.242424 | Rejected |
| SLPI | 3.28565 | 3.279427 | 1.552444 | 4.885618 | 0.575758 | Tentative |
| TACSTD2 | 2.720707 | 2.640998 | 0.544083 | 4.486847 | 0.393939 | Tentative |
| TIMP1 | 4.850538 | 4.892161 | 2.671001 | 6.247642 | 0.878788 | Confirmed |
| TRIB3 | 1.87699 | 2.118228 | -1.3448 | 3.486544 | 0.060606 | Rejected |


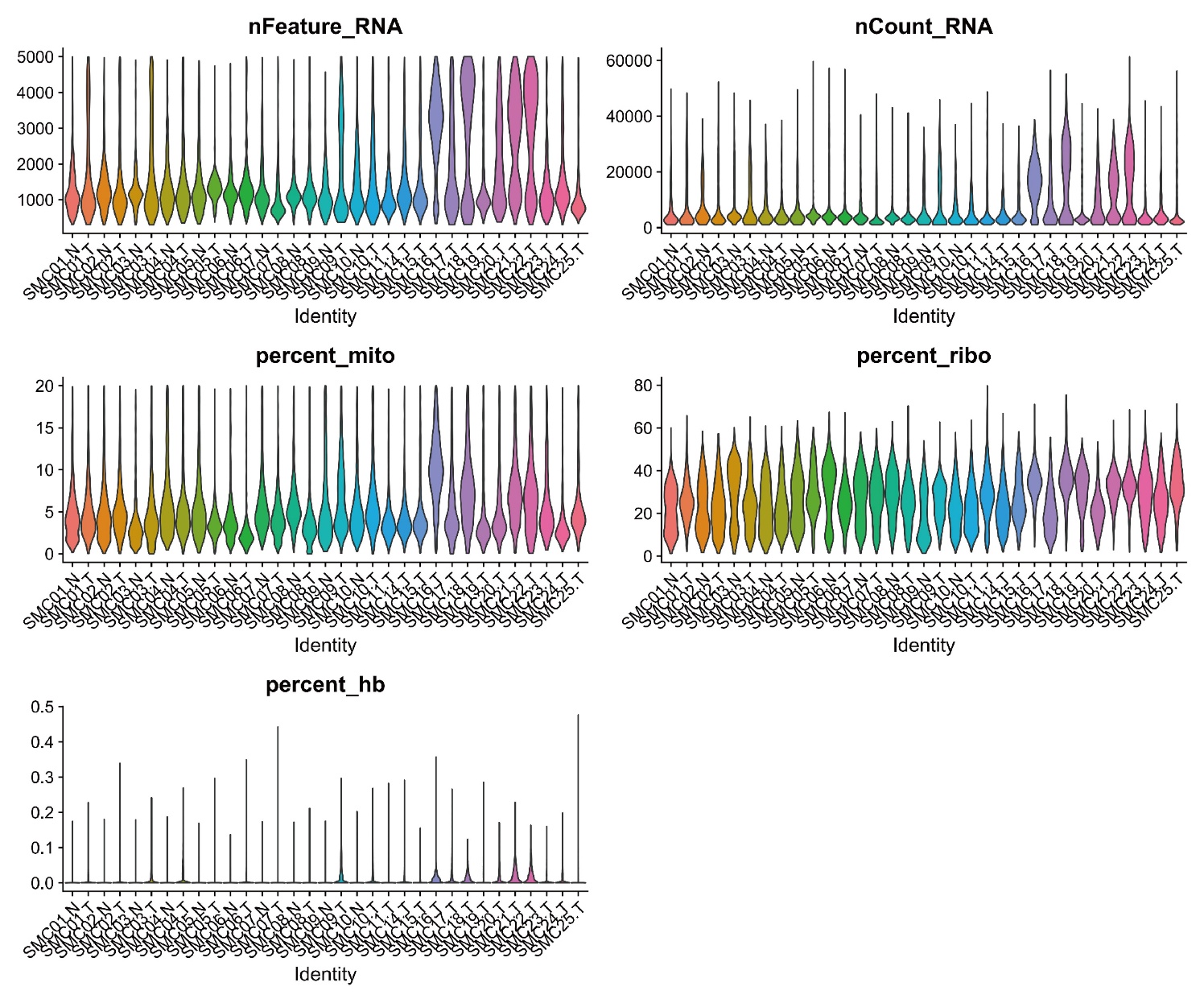


Supplementary Figure 1： Quality control


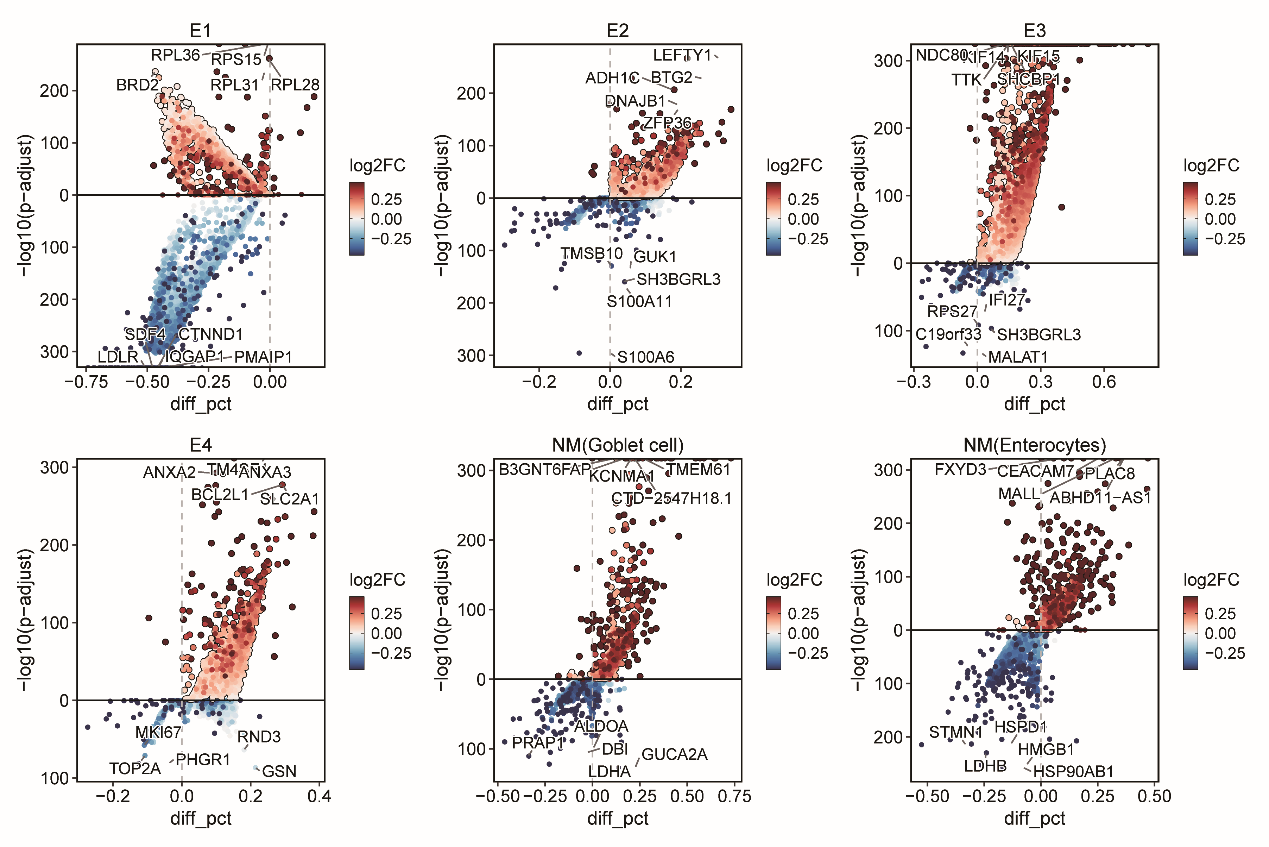
 Supplementary Figure 2：DEGs between different subtypes of epithlial cells.


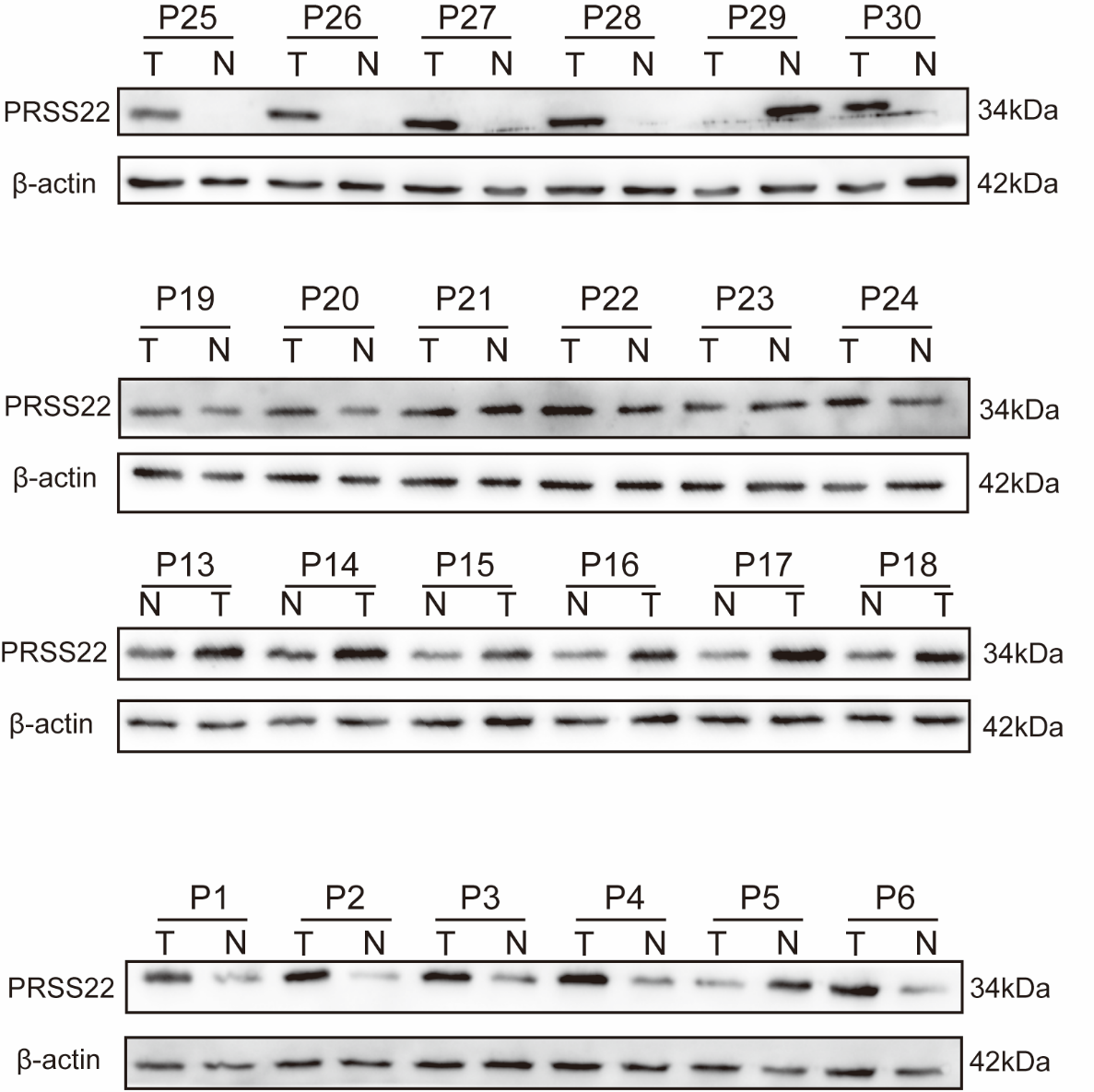


Supplementary Figure 3：Western blotting results in all 24 pairs of samples
